# On the use of local weather types classification to improve climate understanding: An application on the urban climate of Toulouse

**DOI:** 10.1371/journal.pone.0208138

**Published:** 2018-12-12

**Authors:** Julia Hidalgo, Renaud Jougla

**Affiliations:** 1 National Center of Scientific Research (CNRS), Laboratoire Interdisciplinaire Solidarités Societés Territoires (LISST), Toulouse II University. 5, Toulouse, France; 2 Université Toulouse Jean Jaurès, Laboratoire Interdisciplinaire Solidarités Societés Territoires (LISST), Toulouse II University. 5, Toulouse, France; Universidade de Aveiro, PORTUGAL

## Abstract

This paper proposes a method based on a local weather type classification approach to facilitate analysis and communication of climate information in local climate studies. Presented herein is an application to urban climatology in Toulouse, France, but the method can be used in other applied fields of climatology as well. To describe the climatic context of this urbanized area, the local weather types that explain the plurality of weather situations Toulouse faces are presented in depth. In order to show the potential for use of this approach, this information is applied to the study of changes in local weather types in terms of frequency and intensity within a series of future climate projections, a classic urban canopy and a series of atmospheric boundary layer analyses, and as a support for communication aimed to initiate urban climate awareness in urban planning practices. The proposed classification method has been coded in an R script and is provided as a supporting information file. The paper concludes that a systematic pre-study using this kind of climatic analysis is a good practice for performing climatic contextualization in local scale applied studies, both for scientific analysis and communication.

## Introduction

Urban climatology deals with the fundamental physics, chemistry and biology of urban atmosphere at the meteorological and climatological time-scales. For this purpose, researchers observe and model the exchanges of heat, humidity and pollutants between the urban surface and the atmospheric layer directly modified by it, the Urban Boundary Layer, UBL [1]. Nowadays, urban climate literature is composed of studies concerning the understanding of main urban boundary layer phenomena: the Urban Heat Island (UHI) at all heights (surface, urban canopy and boundary layer heat islands), turbulence and city-driven or slope thermal circulations, precipitation trigger, Surface Energy Balance (SEB) modification, etc., and the ways to observe and model them. Several review papers have been published compiling and analyzing scientific production in all of these aspects. See for example Stewart (2011) [2] and Arnfield (2003) [3] for UBL theoretical basis and Barlow (2014), Masson (2006), Kanda (2006) and Dabberdt (2004) [4–7] for progress on atmospheric measurements in urban areas and modelling based on both numerical and experimental approaches. A first compilation on investigations of Urban-Induced Rainfall was made by Shepherd (2005) [8] and the potential of remote sensing on urban climate studies was demonstrated by Voogt and Oke (2003)[9].

In urban climate studies, scientific analysis is sometimes based on monthly or annual indicators, but more frequently, analysis is based on a shorter period. Often the approach focuses on several days of stable weather conditions, but not exclusively: it can also focus on frontal weather, especially precipitation or strong advection conditions, depending on the study’s objectives. The thermodynamic properties and depth of the UBL directly depend on the weather situations that develop on the meso-scale, and that is why a good description and understanding of the climatological context in general and the weather situation in particular are needed when performing scientific analysis on the local scale. Systematic methodological problems in this sense were alluded to by several researchers in the past few decades [10–16]. In Stewart’s study published in 2011 [16], a systematic review and critique of urban heat island literature is presented for 190 urban heat island studies made over the period 1950–2007. Results showed that studies were both “unsuccessful in carrying out observations of sufficient duration or frequency to meet the stated aims of their investigation” (37% of the sample) and “failed to control the disturbing effects of the weather situation on the UHI magnitude evaluation through planned sampling design or post hoc data correction or selection of the weather situations” (45% of the sample).

This paper aims to propose a method based on a local weather type classification approach, to facilitate analysis in urban climate studies. A pre-study identifying the plurality of weather situations representative of a place provides insight into the climatic context of the study, such as the representativeness of the meteorological phenomena studied in terms of relative frequency instead of other weather situations, but its potential for use is wider. This approach can be also used to analyze changes in specific weather situations (such as heat and cold waves or strong wind situations) in terms of frequency and intensity within both the past observational records and the future climate projections; or even to fit the duration of field campaigns and modelling and orient the post processing data analysis to meet the objectives of the investigation. The main advantage of this approach is that it allows for shifting from a mean climate state typically driven by classic climate indicators to a discourse based on shorter meteorological time-scales. These are more intuitive and useful for scientific production and communication in impact studies related, for example, to human health, building energy consumption or urban planning guidelines, which are all currently active and dynamic themes in the research community.

A Local Weather Type (LWT) refers here to the description of the atmospheric situation directly stemming from the analysis of climatic data from the atmospheric boundary layer. Unlike the synoptic Circulation Type Classifications (CTCs) or the “classic” Weather Type Classifications (WTCs) that refer to large scale circulation patterns in altitude—which, depending on the application, can also be related to near-surface local variables [17]—the notion of weather type used in this paper refers to the series of states of the atmosphere above a location in their usual succession that define its local climate. The notion of “local” refers here to the atmospheric conditions representative of the background rural area surrounding the city. In this context, a weather type analysis allows for a transition from the notion of local- or micro-climate to the establishment of large categories where each weather type corresponds to a meteorological situation that frequently occurs with very similar constituents producing practically similar effects and that represents the plurality of weather situations representative of a place.

A weather type approach has already been applied to urban climate studies by some authors. The Cantat [18] study, for example, presents the influence of weather types on the intensity, duration, frequency, shape and extension of the Paris UHI. Cloud cover, atmospheric pressure and wind were used as explanatory variables to identify weather situations creating a maximum of UHI intensity. The author pursued this method [19] more thoroughly to include temperature and precipitation, obtaining, on an annual basis, 64 possible typologies (four classes for cloud cover, four classes for wind, two classes for temperature and two for precipitation) of weather types for the period 1991–2010. LWT definitions were based on thresholds that were defined before classifying them and the characteristics of each class were established regardless of the input dataset. They applied this method to 60 meteorological reference stations near urban sites in France. One of the advantages of this approach is that it allows comparison of typologies obtained on distant territories. One of the drawbacks is that the number of classes and the thresholds are fixed a priori and therefore do not allow for optimizing the climatic characterization on a case-by-case basis. This kind of approach was also applied by Alcoforado in 2004 [20], which highlights the convenience of the weather type approach in understanding visitor numbers in a tourist area of the Lisbon coast. In their study, on-site meteorological data were measured during 120 field surveys carried out in summertime in 1994, 1995 and 1996. LWT classification was carried out for each survey based on cloudiness (3 classes) and Physiological Equivalent Temperature indicator (3 classes). For the same time periods, two types of indicators of tourist numbers were selected: number of cars parked by the beach, and subjective classification of business by two restaurants/coffee shops proving significant correlation between weather types and turnout factors.

The weather type approach was used by [21] to determine weather situations relevant for the nocturnal UHI in Hamburg, Germany. Unlike previously cited studies, the classification was based on ERA-40 reanalysis, and large-scale weather patterns at 700 hPa were defined in a seasonal base. The objective of the study was to downscale regional climate projections on the order of 10 km of horizontal resolution, to provide urban climate results via a statistical-dynamical downscaling with low computational costs. To do so, the urban heat island pattern was obtained using numerical simulations with METRAS model (https://www.mi.uni-hamburg.de/en/arbeitsgruppen/memi/modelle/metras.html) with a 1-km resolution under present and future climate. In this study an objective weather pattern classification based on a k-means cluster analysis was applied. The advantage of this method is not to build classes a priori; the classes are thus defined progressively by using thresholds that evolve and that make it possible to distinguish the input data as clearly as possible.

Objective and automatic LWT classification was also the strategy we used [22] to describe the climate variability of two French cities, Toulouse and Paris. The main objective of the study was to obtain long term atmospheric inputs at an hourly time step to force Soil-Vegetation-Atmosphere Transfer (SVAT) models needed in impact climate studies. For that purpose, a pre-study was done based on a statistical clustering k-means method for the period 1998–2008. In this study LWTs were obtained on an annual basis and it is only subsequently that their characteristics are described at the seasonal time scale. In this study about a dozen clusters seemed to be enough to describe the local climate in terms of wind regimes, precipitation and thermal and humidity amplitude for the two cities. There are two main advantages to this approach. First, it is based on near surface atmospheric data and, even if atmospheric expertise is needed to interpret the resulting weather situations, there is no need of in-depth knowledge of large scale (horizontal length scale over 1000km) climate dynamics and large amounts of atmospheric data as is the case for WTCs; and second, when used to explore future projections, the method is able to produce extremes higher than those observed during the period used to construct the weather type classification, so it is suitable for climate change impact and adaptation studies.

Here the LWT classification approach is proposed as a systematic good practice when performing climatic contextualization and analysis in urban climate studies. This proposal is demonstrated through two different applications:

- First, a classic urban canopy and boundary layer analysis. The objective is to test the weather type classification method through a typical set of UBL analyses in order to show: first, the value of enriching the weather situation description and second, the impact of the LWT approach on the calculation of indicators and analysis of phenomena.
- Second, as a support for communication to initiate urban climate awareness in urban planning practices. This is illustrated through the example of Toulouse during the first stages of urban climatologists’ collaboration with the local Municipality and the Urban Planning agency.

## Materials and methods

The statistical clustering method proposed in Hidalgo et al. (2014) [22] is presented and commented on herein. The result of this classification is directly reused to develop subsequent sections. For the sake of simplicity, the terms local weather types and cluster appellation will be used below without distinction.

### Input data

Meteorological fields used to obtain the LWT must come from hourly data from a rural site situated under the same weather regime as the city. The data that are used may be from a single observational site, from a reanalysis or from modelled data. Observational data is available from the operational network of surface stations with wide coverage around the world (http://worldweather.wmo.int/). Modelled or reanalysis near-surface data can be useful when long series of observations are not available or, if they are, are not homogeneous, but they will require a supplementary step of verification and pre-treatment. Data must cover a sufficiently long period, so the full diversity of local weather types affecting the site will be represented, typically over about ten years. This period will be referred to here as the construction period.

The climatological period of 1998–2008 from the Blagnac weather station located 8 km NW of the city center of Toulouse presented in Hidalgo et al. (2014)[22] is chosen here to illustrate the aims of this paper. The variables involved in the classification were the daily thermal amplitude (Tmax-Tmin, °C) and the daily means of specific humidity (g kg^-1^), precipitation (mm h^-1^), wind speed (m s^-1^) and wind direction in four quadrants (1–90; 91–180; 181–270 and 271–360). The objective was to obtain from this dataset a set of clusters representing the plurality of weather situations representative of the on-site local climate. The classification was applied on an annual basis, all seasons taken together, and the seasonal characteristics in terms of diurnal cycle for each variable were interpreted and computed afterwards.

### Statistical method

The k-means clustering method was used by Hidalgo et al. (2014)[22]; nevertheless, the statistical Partitioning Around Medoids (PAM) method [23] is recommended here for future studies. Both methods are classic partitioning techniques of clustering that cluster the data set of *n* objects into *k* clusters. Both methods attempt to minimize the distance between points labelled to be in a cluster and a point designated as the centre of that cluster; but the difference is that in PAM, aggregation is done around points from the existing data set, called midpoints or centroids, and no longer around fictitious points corresponding to the average of each class, as is the case in k-means. This is useful for selecting a day representative of the local weather type instead of a mean situation that aggregates days around the centroid. At each iteration, points in the dataset change classes. Classification stops either when there is no exchange between classes, or when the assignment of the points in the dataset to each class stops changing. In these methods (k-means and PAM) the distance measurement determines how the similarity of two elements is calculated when forming clusters. In Hidalgo et al. (2014) [22], the Euclidean distance was used; and to avoid differences in scale among the variables, the standard deviation was used to normalize their values. The PAM method allows a *dissimilarity matrix* to be used, facilitating the treatment of wind direction as a qualitative variable based on Gower distance as a metric.

Both methods produce very close results in terms of number of clusters, general features and frequency distribution, but as centroids are calculated differently, the climatology of cluster number can differ slightly. For Toulouse, a relatively high number of applied studies already use the classification obtained in 2014 [22]; this explains why the analysis is based on that cluster series here. The PAM method was nevertheless applied to obtain the LWT for fifty cities in France [24] in the framework of the nationally-funded MApUCE project (http://www.umr-cnrm.fr/ville.climat/spip.php?rubrique120&lang=en) proving very good results.

The classification method outputs are:

- a cluster number attributed to each day of the construction period.
- a table of centroid characteristics. This is the daily thermal amplitude and the daily means of specific humidity, precipitation, wind speed and wind direction for the day corresponding to the centroid. This table can be used to attribute a cluster number to each day of a new climatic period through the Euclidean distance between each variable and the values from centroids.
- if the PAM method is used, the dissimilarity matrix.

Some diagnoses can be calculated from these results, such as:

- the mean diurnal cycles per local weather type. These cycles can also be detailed by season to explore the seasonal variability.
- the seasonal frequency distribution per local weather type within the construction period.
- for temporal downscaling, reconstructed hourly time-step series using the observed (or modelled) mean daily value and the shape of the cluster’s diurnal cycle for the season of this particular day. For temperature, maximum and minimum values are used to reproduce the shape amplitude of the cluster’s diurnal cycle that is modulated as follows:
  $$T{\left(h\right)}_{i}={\bar{T}}_{i}+{\left(\Delta \text{T}{\left(h\right)}_{\text{seasonmean}}\right)}_{k}*\text{Cte}$$
  $$\text{Cte}=\frac{{\left(T_{\text{max}}-T_{\text{min}}\right)}_{\text{obs}}}{{\left(T_{\text{max}}-T_{\text{min}}\right)}_{k}}$$
  Where *i* is the day number and k the cluster number. T_max_ and T_min_ are the maximum and minimum values of the observed (or modelized) field. For a cluster *k*, ΔT(*h*)_season_mean_ is the seasonal mean hourly cycle of ΔT(*h*) being $\Delta \text{T}(h)=\text{T}{\left(h\right)}_{j}-\bar{T}{\left(h\right)}_{j}$.

A definition of the LWT based on daily parameters is pertinent as UBL dynamics and features of SEB and UHI are strongly influenced by the daily cycle. This does not preclude a posteriori analysis of concatenation of local weather types that follow one another in succession and that respond to a large-scale WP on a longer time scale.

### Optimal number of local weather types

To identify how many local weather types (clusters) are needed to properly describe the local climatology of a particular place, an iterative exercise increasing the number of clusters is performed and some rules are defined to fix the optimal number of local weather types.

There are different means of evaluating the resulting clustering quality in a quantitative manner [25] and specific criteria should be defined to meet the objectives of the study. In [22], the objective was to obtain the best series reconstructed at hourly time step once a local weather type was attributed to each day of a future projection of climate at daily time step. To evaluate the optimal number of LWTs, the multivariate Root Mean Square Error (RMSE) of the reconstructed and original observed series at hourly time step was used. The optimal number of LWTs chosen corresponded to a local minimum between 4 and 25 clusters. This criterion is still valid in the present study as the objective is to obtain a set of clusters that represents the local climatology. When using the PAM method a dissimilarity matrix can be applied to compute the differences between the reconstructed and the original series and is here recommended than a multivariate RMSE. Tests effectuated in the framework of the MApUCE project show that iteration between 7 and 15 clusters is enough to optimize the classification and saves a considerable amount of time.

In Hidalgo et al. (2014) [22], a second indicator was used, the attribution frequency deviation. This second indicator was developed to fit specific needs of the study and it is not needed for standard applications. More details concerning this indicator can be found in S1 Appendix as supporting information to the paper.

### Local weather types calculation script

The clustering method including the iteration exercise to obtain the optimal cluster number has been coded in an R script and is available in the supporting information materials S1 Appendix. This code is under GPLv3 license.

The script requires at least the 3.3.3 R version. It was developed with a linux—Ubuntu (32 bit) platform. Some specific libraries are used and are needed at least for this version. In particular, this is the case of the cluster library, which contains k-means and PAM functions. Moreover, for the post-classification analysis, the plotrix library allows wind roses to be plotted. A pre-treatment routine allows application of the main script to any type of data, observed or simulated, and for any time periods.

## Results: Local weather types for Toulouse

Here the general LWT overview presented in Hidalgo et al. (2014) [22] is included; subsequently, both the detailed description of each LWT and a frequency study based on future projections not published before will be introduced.

### LWT general overview

**Fig 1:**
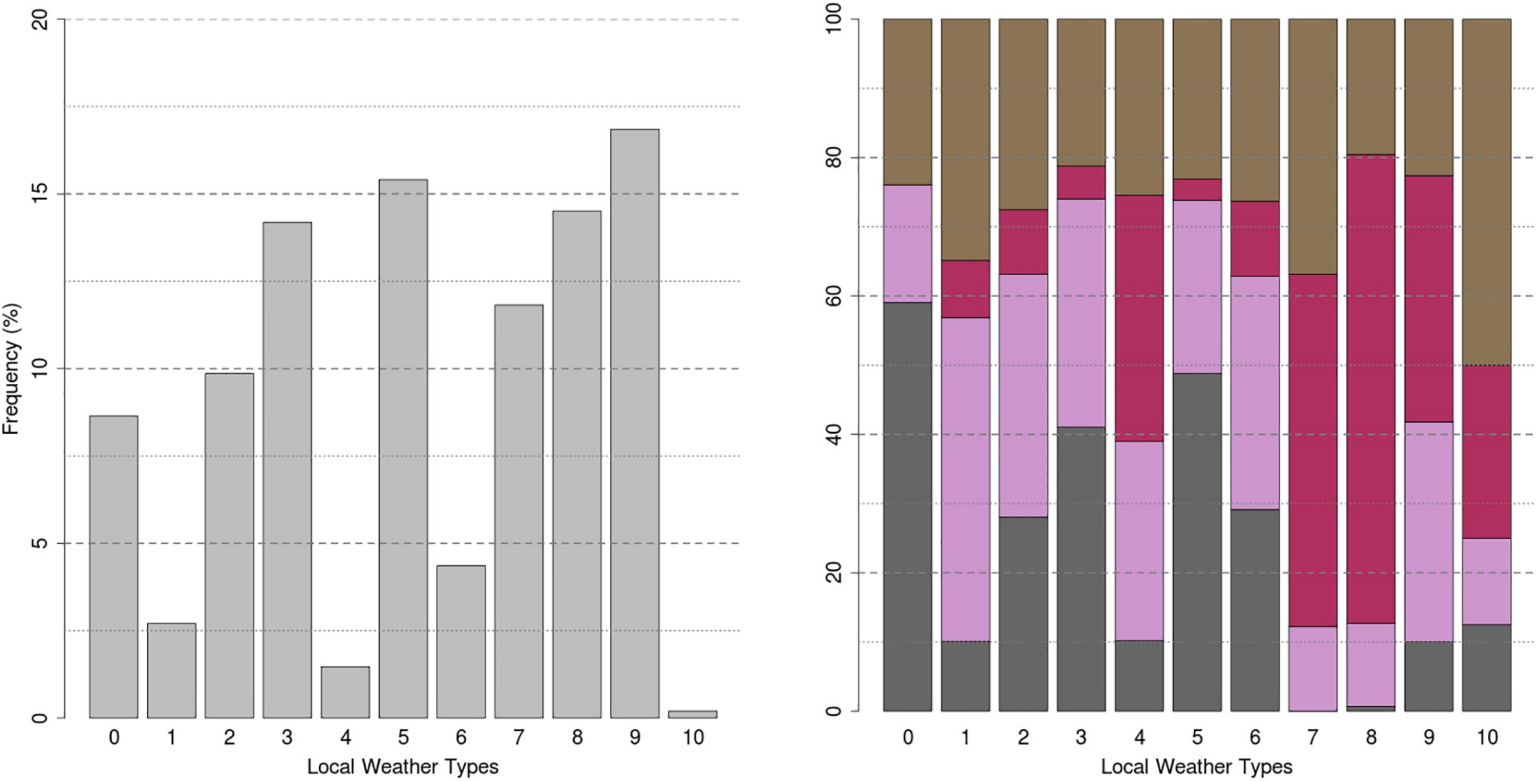
Cluster frequency occurrence in total percentage (left) and per season (right). Spring, summer, autumn and winter seasons are in brown, pink, purple and grey respectively. (adapted from [22]).

Table 1 reports the centroid characteristic of each variable and Fig 1 presents the occurrence frequency (number of days belonging to a cluster) in total percentage and per season. In Toulouse the dominant winds are, in descending order of predominance, the humid westerly wind from the Atlantic Ocean (represented by clusters 0, 3, 4, 6, 8, 9 (the most frequent) and 10), a moderate south-easterly wind (clusters 1 and 7) that can be very strong (called the Autan wind, cluster 2) and finally the southerly wind (cluster 5). Predominant winter clusters are 0, 3 and 5. Representative summertime clusters are 7, 8 and 9. The daily thermal amplitude varies between 3 and 12°C and the daily amplitude in relative humidity between 10 and 50%. Precipitation is concentrated on clusters 1, 4, 6 and 10.

**Table 1:** Variable values for the cluster centroids.

Cluster number	ΔT = T_max_-T_min_ (°C)	FF (m.s^-1^)	DD (quadrants)	PP (mm.h^-1^)	q (g.kg^-1^)
0	5.60	1.43	3.7	0.071	5.07
1	7.31	2.59	2.0	0.297	8.42
2	6.19	6.25	2.1	0.016	7.09
3	3.43	4.27	3.9	0.065	5.07
4	6.44	3.13	3.6	1.003	9.42
5	9.75	2.37	1.9	0.012	5.67
6	5.05	4.56	3.7	0.436	7.21
7	11.08	2.62	1.9	0.014	10.45
8	7.23	2.38	3.8	0.089	10.82
9	12.49	1.80	3.8	0.059	7.03
10	4.15	5.01	3.1	1.938	9.03

Diurnal amplitude of air temperature (ΔT) and daily mean values for wind speed (FF), wind direction (DD) expressed in quadrants 1 to 4 for ranges [1–90]; [91–180]; [181–270] and [271–360] respectively direction, precipitation (PP), and specific humidity (q) (adapted from [22])

### Detailed description of LWTs

In this section, the LWT situations are described as well as their seasonal intra-cluster differences. Cluster characterization is based on the meteorological variables involved in the classification (T_max_-T_min_; daily means of specific humidity, precipitation and wind) and also on fields not involved in the classification, but available from the Blagnac weather station (air pressure, relative humidity and finally global and scattered short-wave solar radiation). Figures presenting the seasonal mean hourly cycles are presented in S2 to S12 Figs in S2 File.

There are four types of clear sky days that feature prominently in the region and are characterized by situations of high atmospheric stability and strong insulation:

#### Typical sunny summer day with westerly-northwesterly wind (Cluster 8)

This is the most common day type in summer with over 50% of the days of the cluster. The temperature is relatively high, both in summer where the summer T_max_ is 30 °C (being the average T_max_ of the cluster of 25 °C), and in winter, where the winter T_min_ is 10 °C (the T_max_ average of the cluster is 13 °C) for a wind from west-northwest that can be relatively strong, especially in summer and winter with peaks in the afternoon at 4 and 6 m.s^-1^ respectively. There is no heavy precipitation but the specific humidity is quite high in summer and autumn (> 0.01 kg.kg^-1^).

#### Sunny day, very hot in summer, with northwesterly wind (Cluster 9)

This is a type of weather frequent in all seasons. In summer, the temperature can reach 40 °C, but the days are cold and sunny in winter (the average T_max_ of the cluster is 10 °C). The wind blows from the west in the morning to northwest at noon and then southwest again in the evening. Wind speed also varies from 2 m.s^-1^ in the morning to 4 m.s^-1^ in the middle of the day to 2 m.s^-1^ again at the end of the day.

#### Typical sunny summer day with weak southeasterly wind (Cluster 7)

This is a typical summer day type with more than 50% of the days of this cluster. It does not occur in winter. In summer, the temperature can reach 40 °C, characterized by a large thermal amplitude (~11 °C) and a persistent but weak southeasterly wind (2–3.5–2.5 m.s^-1^ in the morning, afternoon and evening respectively).

#### Typical sunny winter day with weak southeasterly wind (Cluster 5)

Very sunny situation typical of winter. The maximum global solar radiation, RGmean_max_, is 800W.m^-2^ in summer and 250 W.m^-2^ in winter. It is uncommon in summer with high temperatures (T_max_ = 30 °C, average cluster T_max_ = 27 °C) and cold in winter (Tmean_min_ = 0°C, average cluster Tmean_max_ = 9 °C). The wind is very weak, in mean from 1 to 2 m.s^-1^, and blows from the south in the morning with a slight turn to the southeast in the afternoon. This type of day is very common in the Toulouse region.

There are two types of day that correspond to southeast wind flows:

#### Autan wind day (Cluster 2)

This type of LWT is relatively frequent especially in spring and autumn. Wind strongly flows from the southeast (4–7–4 m.s^-1^ on average in the morning, noon and afternoon respectively). The wind is weaker in summer but this type of weather seldom occurs in this season. The anomaly in the direction observed for some days of this LWT in summer is due to the fact that the K-means method applied by Hidalgo et al. (2014)[22] does not use the dissimilarity matrix facilitating the treatment of wind direction as a qualitative variable. When using the PAM method, the classified days coincide perfectly with the predominant wind direction. There is no appreciable precipitation for this type of day. It corresponds to the so-called Autan wind, which is a south-easterly, föhn-type wind from the Mediterranean that can be strong.

#### Rainy day with southeasterly wind (Cluster 1)

This is a type of day that looks like the previous one in terms of temperature but is much less frequent. The wind is lower (2 m.s^-1^ on average) and the rainfall is relatively high in the afternoon. The wind can reach 5 or 6 m.s^-1^ on average in summer, but there are very few days allocated to this cluster in this season (9 days). It is a type of day more common in the intermediate seasons (spring and autumn). It probably corresponds to other south-easterly winds that may or may not be related to the Autan, called the “Autan noir” (black Autan), a warm wind, precursor of intermittent rain and less frequent than the Autan wind.

The most intense rainfall is concentrated on two types of days and remains relatively infrequent:

#### Rainy day (Cluster 4)

This type of day is highly precipitous in the afternoon with cloudy skies (RGmean_max_ ~300 W.m^-2^ in summer and ~150 W.m^-2^ in winter). The northwest-westerly wind is weak in the morning (2m.s^-1^ on average) and accelerates in the afternoon by the presence of thunderstorms. Rain causes the specific humidity level to be relatively high in summer and autumn (average> 0.01 kg.kg^-1^). This type of day is not very common but its distribution is balanced throughout the year.

#### Very heavy rain day (Cluster 10)

More intense rainfall than in the previous cluster, both in the morning and the afternoon. It features a strong wind from west-northwest. Only 8 days are allocated to this cluster, half of them in autumn.

Finally, three types of days with high or low NW wind and with or without precipitation are characterized by the following clusters:

#### Typical winter day with low rainfall (Cluster 0)

Weak wind that is fairly constant in intensity, around 2 m.s^-1^ which starts as a west wind in the morning, shifts to southwest at mid-day and becomes westerly in the evening. This is a typical type of winter day, with 50% of the days of the cluster, little diurnal thermal amplitude (~5 °C) and a little precipitation. It does not occur in the summer.

#### Day with strong northwesterly wind typical of intermediate seasons and winter (Cluster 3)

Very frequent in the intermediate seasons and winter. Persistent strong wind (4—6—4 m.s^-1^ on average) from the northwest.

#### Cloudy day with strong NW wind and precipitation (Cluster 6)

Strong northwesterly wind (4—6—4 m.s^-1^ average) with precipitation all day long. This is a type of day with a very low thermal amplitude (~5 °C), which results in particularly cold days for summertime (average T_max_ = 18 °C). It is typical of intermediate seasons and winter, the summer GR cycle shows a deformation in the morning, probably due to a strong cloudy presence.

### LWT in future projections of climate

It is also possible to attribute an LWT number to each day of a period outside of the *reference period*, for example to historical or past observations or future projections of climate. The application of the attribution method to climate projections is possible assuming that under future climate conditions the occurrence frequencies of clusters might be modified but their dynamic characteristics remain unchanged. It is the same hypothesis used for methodologies based on weather classes and this approach is supported by studies suggesting that anthropogenic climate change may manifest itself as a projection onto the pre-existing natural modes of variability of the climate system [26–29].

To achieve the scientific objectives in Hidalgo et al.(2014)[22] paper, an LWT was attributed to each day of a series of future climate projections produced by several Regional Climate Models (RCM). The Climate Service Center of the Max Planck Institute for Meteorology (MPI) provided the time-series at the nearest grid point from REMO (https://www.remo-rcm.de/059966/index.php.en#tab-1) simulations under A1B, B1, and E1 emission scenarios [30]. Six model outputs under the A1B emission scenario from the EU-FP6 ENSEMBLES were also used: ECHAM5-r3–RCA, ECHAM5-r3–RACMO, ECHAM5-r3–REMO, HadCM3Q0–HadRM3Q0; BCM—RCA and BCM-DMI–HIRHAM5 (the first name corresponds to the forcing Global Climate model, the second name to the Regional Climate model).

Figures are presented in S13 and S14 Figs in S2 File Results shows that, when comparing the MPI or ENSEMBLES scenarios between them separately, the frequency distribution is very close between scenarios. The distribution is also quite close, along general lines, to the distribution on the present climate (Fig 1). This means that the LWTs defined for Toulouse using the reference period are relevant in the future projections, backing up the hypothesis that in principle there is no new LWT in the future. What will change is the intensity and internal variability of the LWTs, but the distribution will not be too far from that of the prevailing climate. For MPI projections, rainy LWT (1 and 4) and LWT 5 will be more common in the future than in the present climate. Cool LWT number 3 will decrease due to a rise in the daily average temperature. For the ENSEMBLES projections, the distribution of clusters is closer to the observed clusters in the present climate than that of the MPI projections. This may be due to the fact that, in the MPI projections, precipitation was not available and then not used for the cluster attribution. In the ENSEMBLES projections the cool LWT, number 0 and number 1, presents the greatest variability.

As we will see in section 4.3, analysis of LWT in future projections is useful for letting urban stakeholders know what changes can potentially be expected due to climate change in a manner other than mean trends conveyed by classic climatic indicators.

## Applications

### Enriching the general description of Toulouse’s local climate

**Fig 2:**
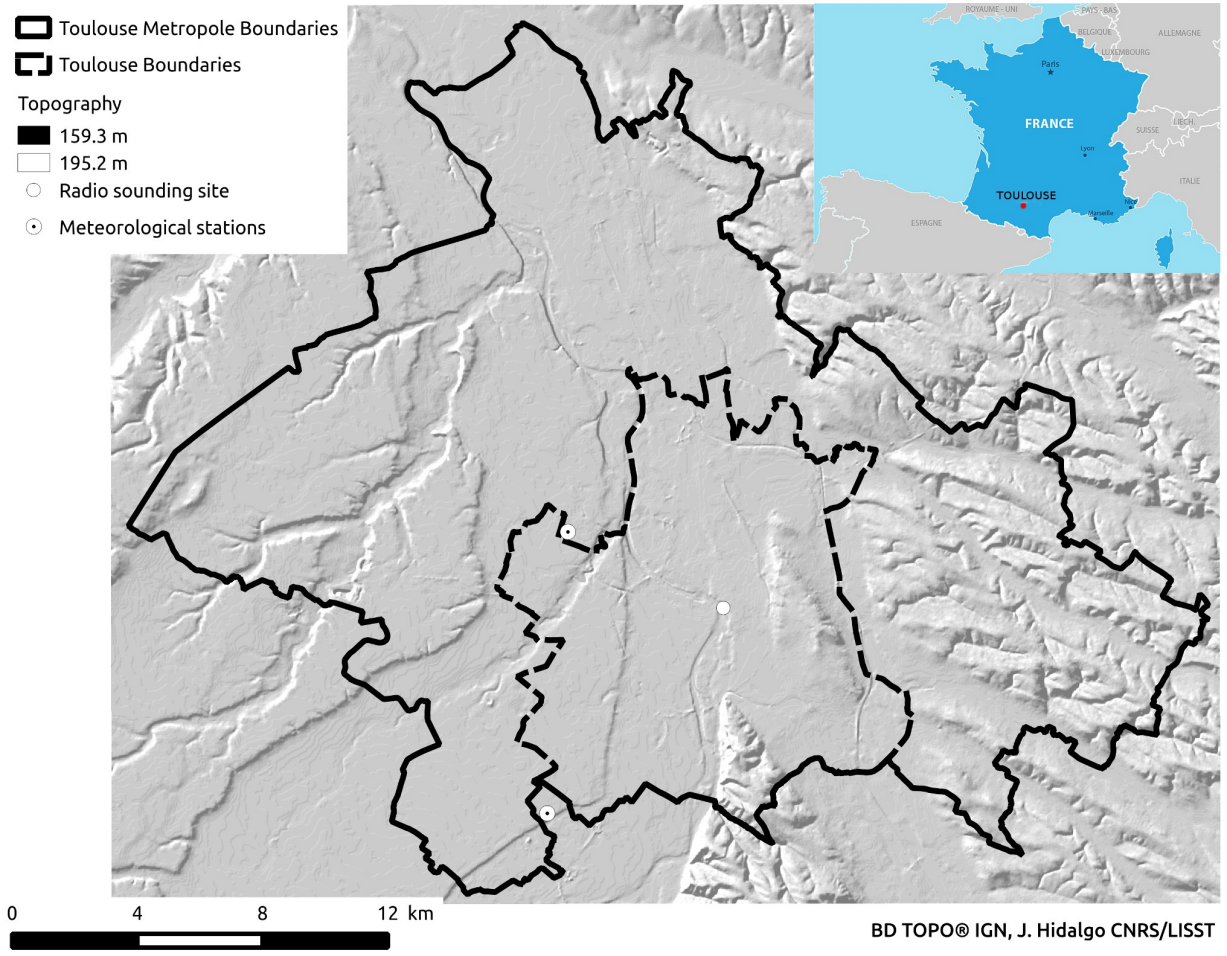
Reference meteorological stations for Toulouse. Toulouse-Blagnac (43.621N, 1.38W) station is situated at an elevation of 151 m above sea level. Toulouse-Francazal (43.540N,1.372W) station is situated at an elevation of 164 m. Radio-sounding site during the CAPITOUL campaign was situated near Capitol place.

**Fig 3:**
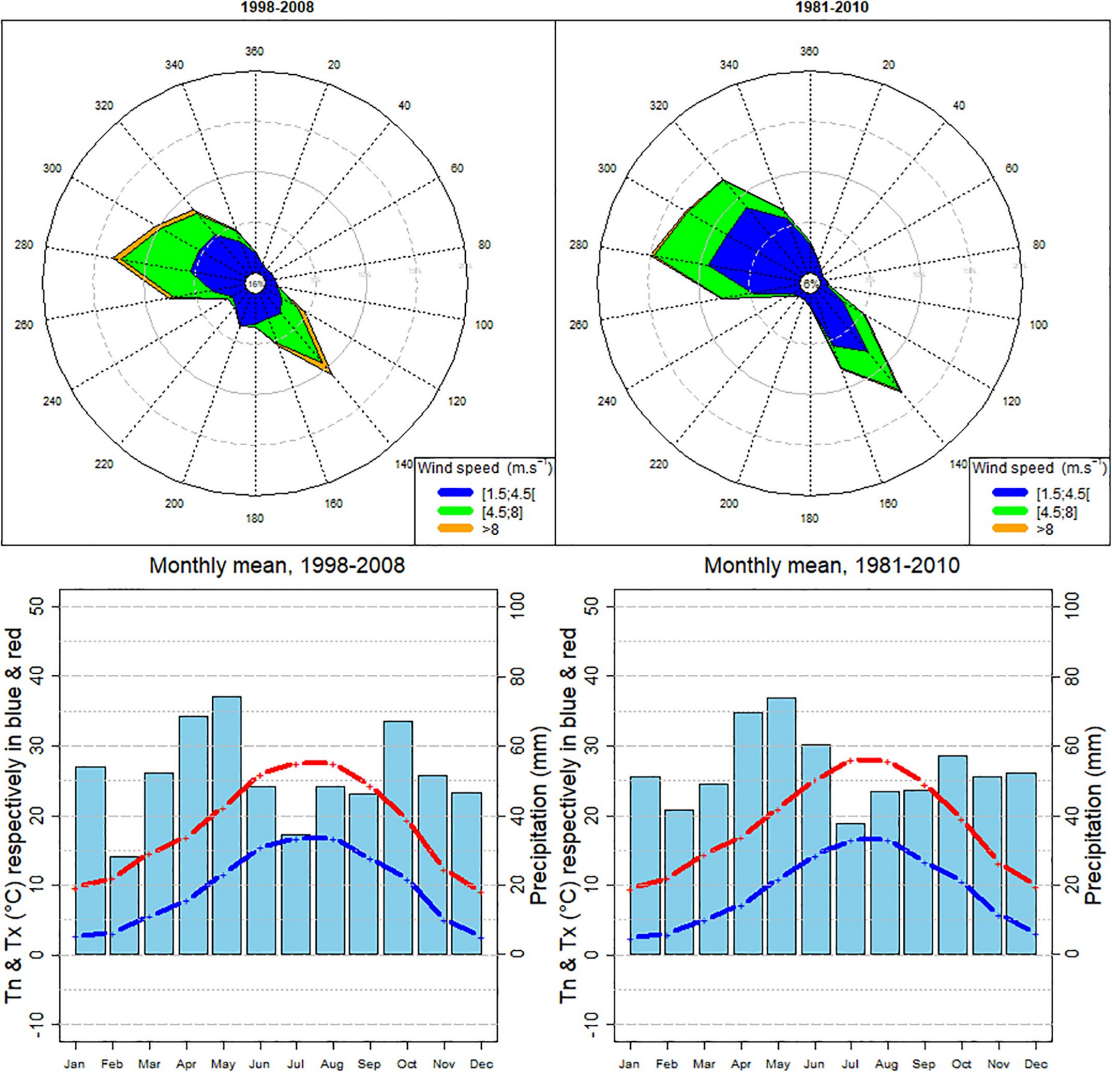
Climatological data at the Toulouse-Blagnac station (Data source, Météo-France). Up: Wind rose for the Reference period (left) and current WMO Climatological Normal Period (1981–2010) (right). Down: Monthly average precipitation and air temperature (maximum, Tx, and minimum, Tn, values) for the same periods respectively.

Information used in Toulouse to describe general climate features was usually based on a combination of regional climate descriptions based on the geographical description of the city and its environment and on classic climatic indicators from Météo-France, the French National Weather and Climate Service. Here is an example of the information that we typically used to contextualize our studies [31]:

“Toulouse is situated in the south-west of France 80km north of the Pyrenees mountain range, 150km west of the Mediterranean Sea and 250 km east of the Atlantic Ocean. The relief is marked by the convergence of the Garonne affluent valleys. The minimum elevation is 102 m and the maximum is 273m. It is open to the Atlantic and the Mediterranean. The Ariege river to the south is dominated by the sloping hills of Vielle-Toulouse village that overlooks the city over the Pech David promontory. The Hers-Mort river that flows to the Garonne north of Toulouse forms a huge plane called “Lalande”. On the East, there is a separation line formed by the Montaudran and Jolimont hills. On the west, six or seven kilometres from the city centre, there are three terraces that reach the Gascogne hills. Toulouse is situated in a geographical space qualified by a “degraded Oceanic Climate”. This area has the particularity of being at the crossroads of three types of temperate climates: an oceanic climate with Mediterranean and continental influence. Seasons are quite marked with relatively chilly springs with strong precipitation and violent thunderstorms, dry, hot summers, a sunny and mild autumn, and quite cold and relatively rainy winters. Unlike coastal or mountainous cities, Toulouse cannot benefit from the cooling brought by the sea and valley breezes respectively. Two meteorological stations are situated within the metropolitan area (Fig 2):

The normal value of the annual mean temperature is 13.8 °C (Fig 3 top). The mean annual precipitation is about 620 mm and the duration of sunny periods is 2,047 hours. Concerning the long-term trends of the climatic indicators, it could be said that, in annual mean, over the period 1951–2012 (the present normal climate conditions), an increase of 0.23°C/decade is observed in the minimum temperature and 0.27 °C/decade in the maximum temperature. There has been an acceleration of the warming from the mid1990s. The warming is more marked in the spring, summer and autumn. There is little change in the precipitation rate but the ground dryness is increasing [32]”.

Fig 3 bottom, shows the same classic climatic indicators but for the Reference period. It can be observed that the Reference period has identical monthly mean thermal fields. Precipitation shows similar behavior with slightly less rainy winters. Wind-rose compares also quite well but south-westerly winds are over-represented. This is not really a problem as wind frequency for this quadrant is very weak < 3% in the reference period and the clustering method does not identify an LWT with persistent winds from this direction.

These classic climatic indicators are useful for describing the general context and long-term trends but, as the local scale allows us to experience atmospheric conditions at the weather time-scale, it seems pertinent to take advantage of this fact to enrich the climatic description and orientate research.

### Applying the local weather type approach to a classic urban boundary layer analysis

Through the LWT approach, UBL understanding can be better detailed and results significantly modified with respect to a monthly, seasonal or annual approach. Illustration is done here through a general UBL analysis based on the Surface Energy Balance (SEB) and Urban Heat Island (UHI) intensity evaluation based on urban atmospheric measurements. For that purpose, a dataset from the CAPITOUL field campaign held in Toulouse for a year in 2004 and 2005 was used. Finally, the 3D consistency of the LWT classification is tested through radio-soundings from the same observed database and the diurnal change in the atmospheric boundary layer properties is discussed.

#### The CAPITOUL campaign

The Canopy and Aerosol Particles Interaction in TOulouse Urban Layer (CAPITOUL) experiment was a multi-objective and multi-scale urban climate field campaign developed on the scale of the city of Toulouse as a whole between February 2004 and March 2005. An extensive description of the observational network can be found in [33].

For this study, ground station and radio sounding data are used:

- Ground station network description

The ground station network consisted of:

- 21 temporal light stations situated in the city centre and in the suburban areas of Toulouse. They recorded temperature and relative humidity with 12-minute sampling.
- 5 synoptic ground stations of Météo-France that measured the horizontal wind velocities at a height of 10 m. The temperature and relative humidity levels were recorded at a height of 2 m in a 10-km radius from the city centre. Two of these towers, one 15 km south-west (Le Fauga, situated in a grassland area) and the second one 40 km north-west of Toulouse (Saint-Sardos, situated in an irrigated maize field), measured the solar radiation components and turbulent fluxes with a 30-minute sampling.
- A pneumatic tower situated in the old heart of the city was considered the reference station for describing the properties of the city centre of Toulouse. This central 30-m tower was situated on the roof of a building situated 20m above the ground. Temperature, relative humidity, wind speed, pressure, upward and downward global short- and long-wave radiation, and the turbulent flux measurements were recorded at three different heights (47.5m, 38.5m, 26m above the ground). Depending on expected wind dusts, the mast height was modified, and four positions are thus defined in the dataset (positions 1 to 4 from low to heavy winds respectively). Here we will focus only on data from position 1, corresponding to 163 days of measurements.

- Description of radio soundings

Radio soundings were launched during the twenty Intensive Observational Periods (IOPs) corresponding to around thirty days: ten IOPs during winter, three in spring, five during summertime and two during autumn. They were launched four times a day at 03.30, 08.00, 13.00 and 18.00 UTC from two sites: from Valade’s site situated in Toulouse’s city centre (Fig 2) and, depending on the predominant large-scale wind direction, from Merville, a rural zone situated approximately 15 km north-west of the city centre, or Le Fauga, a rural zone situated approximately 15 km south-west. The analysis is focused here on the Valade site’s radio-sounding.

- The CAPITOUL experiment and the Toulouse LWT

**Fig 4:**
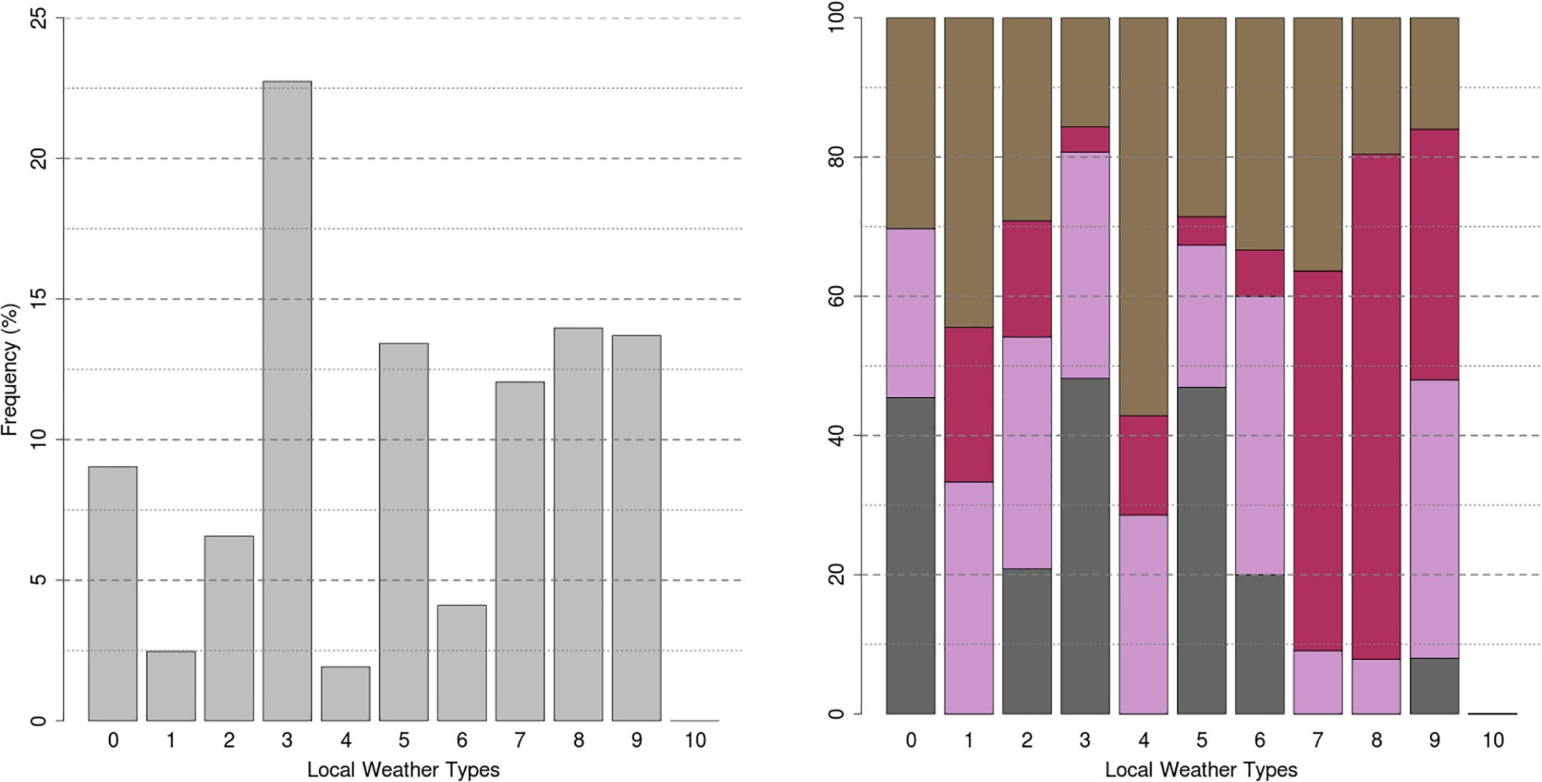
Frequency distribution of CAPITOUL days over the LWT defined for Toulouse (left side) and breakdown per season (right side). Spring, summer, autumn and winter seasons are in brown, pink, purple and grey respectively.

The statistical distribution of the CAPITOUL campaign data is presented here in terms of LWT frequency Fig 4.

On an annual basis, the CAPITOUL campaign period resembles the 1998–2008 climatology presented in section 4.1, although it can be observed that persistent strong north-westerly wind situations (LWT 3) are over-represented during the campaign period. We can therefore consider that the period covered by the campaign is close to the normal in terms of the distribution of local weather types. Differences are a little more marked when the analysis is done by season. Clusters 1, 4 and 8 are not represented in winter, while they were about 10% on the reference climatology. LWT 4, in addition to not being represented in winter, is over-presented in autumn and under-presented in summer. It is the same for 9 with a balance between spring and summer, which are over-represented, resulting in an under-representation of autumn.

#### The Surface Energy Balance analysis from an LWT approach

The energy budget is evaluated here on the basis of the pneumatic tower measurements situated in the city centre. The turbulent fluxes were computed on 30-minute periods with eddy covariance techniques. Calculations were performed by Pigeon et al. (2007)[34] and SEB was evaluated at the top of the street canyon as described by Oke (1988)[35]:

$$Q*+{\text{Q}}_{F}={\text{Q}}_{\text{LE}}+{\text{Q}}_{H}{+\Delta \text{Q}}_{s}{+\Delta \text{Q}}_{A}$$

The flux densities of net all-wave radiation (Q*), upward sensible heat $Q_{H}{=\rho \text{C}}_{p}\bar{\text{w}\prime \theta \prime }$ and upward latent heat $Q_{\text{LE}}{=\rho \text{L}}_{v}\bar{\text{w}\prime \text{q}\prime }$ at the surface were directly measured. The latter two were calculated using the eddy-correlation method. Thanks to the homogeneity of the Toulouse city centre in terms of construction density, building type and materials, the measurements performed during CAPITOUL around the study site have shown that the temperature and moisture horizontal gradients are weak at the local scale [36] and ΔQ_*A*_ was therefore neglected considering the order of magnitude of the other fluxes.

Finally the sum of the heat storage (ΔQ_*S*_, positive for warming) and the anthropogenic heat flux (*Q*_*F*_) was estimated from the residual of the budget as:

$${\text{R}=\Delta \text{Q}}_{s}-Q_{F}=\text{Q}*-(Q_{\text{LE}}{\text{+Q}}_{H})$$

S15 Fig in S2 File shows the net all-wave radiation Q* for all seasons. At this latitude, a well marked daily cycle is observed with a maximum of intensity around mid-day varying from 150 W m^-2^ in winter to 500 W m^-2^ in summer. This energy is distributed in the different terms of the energy balance. In the morning the heat is mainly stored in the urban fabric and is released as turbulent sensible heat into the atmosphere in the late afternoon and evening. During the night it is normal that the sensible flux density presents small positive values. In Toulouse, night-time *Q*_*H*_ <50 W m^-2^ and is comparable in magnitude for winter (the origin is mainly heating) and summertime (the origin is the recovery of stored heat). During the afternoon, the sensible heat flux reaches a mean value of 250 W m^-2^ in summer but only 100 W m^-2^ in winter. Toulouse is a mid-latitude city with very little vegetation and irrigation in the city centre, the evapotranspiration remains lower and *Q*_LE_ shows weak values in winter, spring and autumn (<25 W m^-2^) and it reaches 100 W m^-2^ at noon in summer. This reduced daytime evapotranspiration in the urban area results in increased magnitudes of sensible heat (*Q*_*H*_) and heat storage in construction materials (ΔQ_*S*_) over the entire day. It can be appreciated that high dispersion is associated with these seasonal means and sometimes the dispersion magnitude is higher than the mean absolute value, as it is clearly shown in autumn. Using an LWT approach S16 to S25 Figs in S2 File, this dispersion can be understood, and single weather situations identified and characterised. Focusing again on autumn, it can be seen in Fig 4 that during the CAPITOUL campaign all the LWTs are present and that the fact of working with seasonal means will mix the physical characteristics of very diverse weather situations.

#### Urban heat island evaluation

The UHI intensity is computed here from the ground station network according to four classes of urbanization developed in Hidalgo et al. (2008) [37]: urban areas (4 stations), residential suburban areas (15 stations), non-residential suburban areas (6 stations) and rural areas (2 stations).

**Fig 5:**
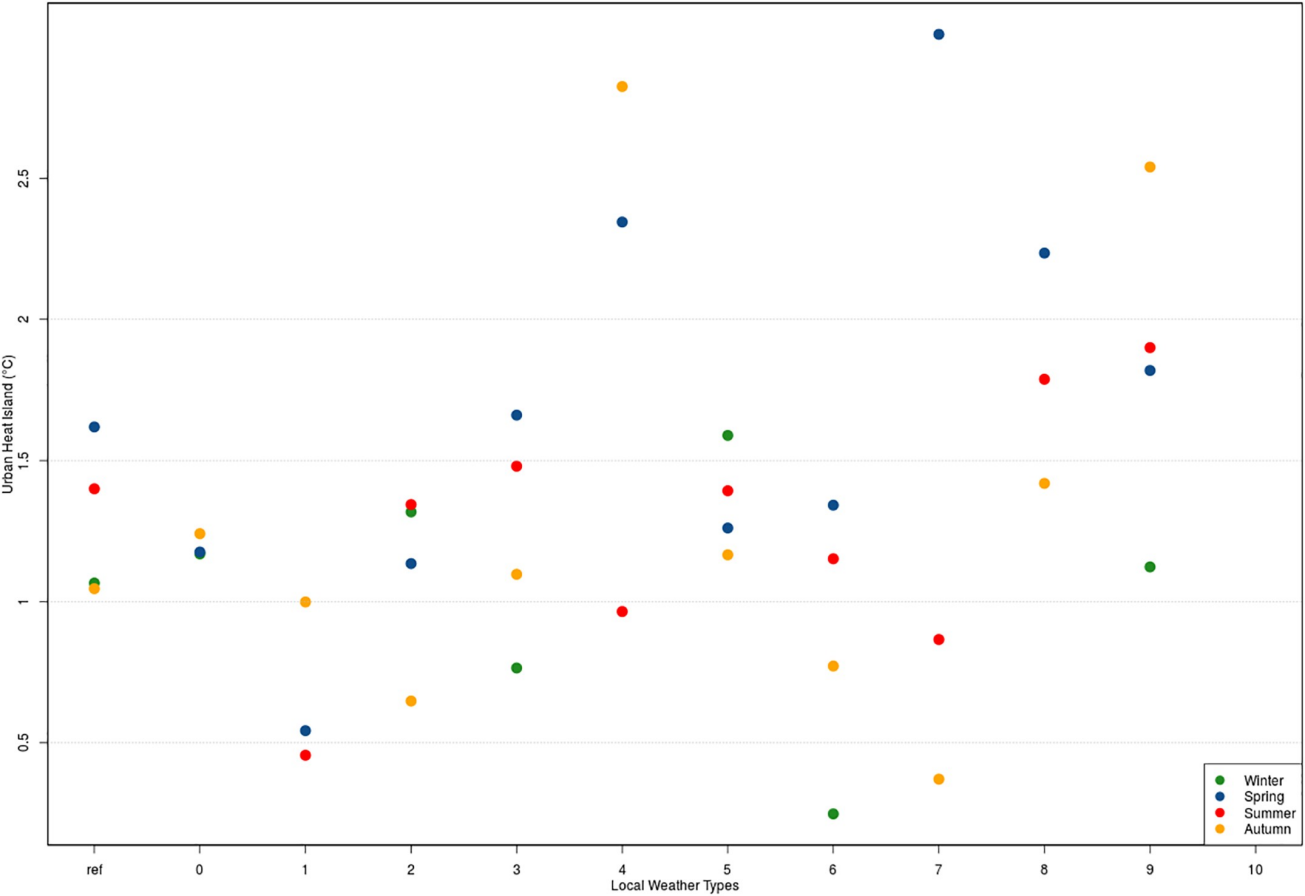
Mean urban heat island intensity from 21h00 to 23h00 UTC. Ref refers to the seasonal mean and 0 to 10 indicates the LWTs number.

Fig 5 shows how the UHI intensity variability is completely masked when the analysis is done seasonally and valuable information is lost, such as which situations are favourable to a strong UHI development or which situations have a stronger UHI in summer or winter and not in spring as indicated by the seasonal mean during the CAPITOUL campaign. It can be observed that for Toulouse the typical nocturnal UHI intensity varies from 0 °C to 2.5 °C and can even reach 6 °C depending on the local weather situation (S26 to S30 Figs in S2 File). The UHIs can develop in Toulouse under meteorological situations that have very different behaviours and for that reason, the time their formation takes, their duration and intensity will vary.

#### Urban boundary layer structure

The vertical structure of the UBL is explored here using vertical profiles of air temperature, specific humidity and wind from radio-soundings. Data are available at high temporal resolution, at 2s time-step. We are interested in surface-atmosphere interactions so only the first 3km are presented, corresponding to around 300 data.

Table 2 presents the availability of radio-soundings for each LWT number. Numbers 1 to 4 make reference to the following time slots: morning (06h00 to 009h00 UTC), midday (10h00 to 15h00 UTC), afternoon (16h00 to 18h00) and night (from19h00) respectively.

**Table 2:** Availability of radio-soundings from CAPITOUL.

	winter					spring					summer					autumn				
		1	2	3	4		1	2	3	4		1	2	3	4		1	2	3	4
**LWT 0**	2005-01-13			X	X											2004-11-24	X	X		
	2005-02-04	X	X		X											2004-11-26	X	X		
	2005-02-10	X																		
	2005-02-24	X	X		X															
**LWT 1**																				
**LWT 2**																2004-10-04		X		X
**LWT 3**	2005-02-03			X	X	2004-03-02	X	X	X	X	2004-07-11	X	X							
**LWT 4**																				
**LWT 5**	2004-12-14	X	X		X											2004-09-29	X	X		
	2005-01-04			X	X															
	2005-01-06			X	X															
	2005-01-07	X	X																	
	2005-02-09			X	X															
	2005-02-25	X	X		X															
**LWT 6**																				
**LWT 7**											2004-06-26	X	X	X	X					
											2004-07-04	X	X	X	X					
**LWT 8**											2004-06-27	X	X	X	X					
**LWT 9**	2005-01-05	X	X		X	2004-03-03	X	X	X	X	2004-06-30	X	X	X	X					
						2004-03-09	X	X	X	X	2004-07-03	X	X	X	X					
						2004-03-10	X	X	X	X	2004-07-15	X	X							
						2004-04-26		X												
**LWT 10**																				

The objective here is to verify if LWTs, which were characterized from near-surface atmospheric parameters, also present coherent 3D structure of the UBL for the days belonging to their group. From the radio-soundings availability table this can be tested only for LWT 0 in winter and autumn (S31 and S32 Figs in S2 File), LWT 5 in winter (S37 Fig in S2 File), LWT 7 in summer (S39 Fig in S2 File) and LWT 9 in spring and summer (S42 and S43 Figs in S2 File). It may be observed that wind profile structure is robust for all those LWTs in the afternoon and night when the local weather situation is well installed. In this sense situations are particularly consistent during summertime from the early morning (LWT 7 and LWT 9). However some time slots show clear differences within the days belonging to the same group, such as, for example, LWT 0 in autumn at 14h00 where wind profiles clearly differ, between the two dates involved (24^th^ and 26^th^ of November 2004). LWT 0 is a southwesterly situation which is well captured in low layers by the radio-sounding on the 26^th^ of November. Differences in wind direction near the surface between the 24^th^ and the 26^th^ of November could perhaps be explained by the turbulent nature of the urban boundary layer but in the upper levels, the difference in wind direction remains clear. Analyzing the LWT chronology, it is possible to say that differences are perhaps due to a residual layer from the previous days because even if the 23^th^ November also belonged to an LWT number 0, a persistent situation of six days with LWT 3 (persistent strong wind from the northwest S5 Fig in S2 File) prevailed the previous week. This impact of chronology in LWT sequence may also explain differences found in LWT 5 (S37 Fig in S2 File), where 14^th^ December and 7^th^ January have LWT 5 as the previous day and 14^th^ January and 25^th^ February have LWT 0 as the previous day.

### The LWT approach as support for communication and decision making

The LWT approach potential for communication and decision making was tested during the French national funded research project MApUCE (Applied Modeling and Urban regulations: Climate & Energy, 2014–2019) during a task related to operational case studies. In this framework, the project team participated from 2015 to 2018 in the working group “Climate and Energy” during the development of the inter-municipal urban plan (Plan Local d’Urbanisme Intercommunal, PLUi in French) by greater Toulouse and its local government. This working group was made up of the municipal technical staff of the environment and urban regulation departments as well as the urban planning agency.

The detailed development of the 11 LWTs for Toulouse in seasons produces too large a number of meteorological situations to be explained and analyzed. After discussion with the local authorities, this number was considered too large to be effectively appropriated by practitioners in the context of operational planning actions. In order to reduce the number of cases, it was decided to set a threshold of representativeness. For the Toulouse region, over 70% of days are represented by seven LWTs:

- Spring: LWT numbers 2, 3, 5 and 9 represent 68% of the days for this season.
- Summer: LWTs 7, 8, 9 with a representativeness of 86.7% this season.
- Autumn: LWTs 0, 3, 5, 7 and 9 with 58.4% of days.
- **Winter**: LWTs 0, 3 and 5 with 67.19% of the days.

The weather patterns are more stable during summer-time and winter-time than in the intermediate seasons, spring and autumn. It is for this reason that in summer and winter, for the same degree of representativeness, the synthesis of the seasonal climate relies on fewer meteorological situations.

The municipal technical staff expressed the desire to further reduce the number of situations to be studied and focus only on four LWT types considered as “situations at stake” in present and future climate for their impact on energy demand, human health or damage due to wind gusts. It was finally decided to focus on the following four LWTs:

- Two situations with strong and persistent wind: the “Autan wind” day (LWT 2), characterized by strong flow from the SE, and LWT 3, corresponding to a NW situation.
- Two stable situations with low wind: one typical of wintertime (LWT5) and one typical of summertime (LWT9).

From this point all the analyses related to UHI, thermal comfort and energy demand are being developed for one or more of these LWTs, as for example the exposition of vulnerable establishments such as hospitals, schools and retirement homes to heat and cold stress, or the development of several Urban Climate Analysis Maps to inform the Recommendation map in the Urban Climatic map [38, 39].

## Conclusions

Like other applied fields of meteorology and climatology, urban climate studies require a good description and understanding of the climatological context of the urbanized area. Authors sometimes decide to base scientific analysis on monthly or annual based indicators, but in urban climatology, studies most often focus on a specific weather situation and the analysis is based on shorter periods. Systematic methodological problems have been alluded to by several researchers in the last decades and the choice of the weather situation, its duration and frequency characterization are mentioned as the most problematic points.

In this paper a Local Weather Type classification approach is proposed as a good practice to be developed when a climatic contextualization is sought for local climate studies applications. The statistical classification method proposed in Hidalgo et al. (2014)[22] is widely presented and some methodological improvements are commented upon. The code source is made available for free use and as recommended by Añel (2011) [40] also for review.

Different types of applications serve to show how this approach is used in urban climate studies in Toulouse, France. We present the study of changing local weather types in a series of future climate projections and that of a classic urban canopy and a series of atmospheric boundary layer analyses, as well as an illustration of how LWTs are used as a support for communication to work with the local municipality and the local Urban Planning Agency.

These analyses allow us to comment on the usefulness of evolving from a mean climate state typically driven by classic climate indicators, to a more intuitive discourse based on shorter meteorological time-scales, both for the description of local climate context and for the impact of the LWT approach on the calculation of indicators and analysis of phenomena.

This method is relatively straightforward, as it mobilizes easily available atmospheric data and statistical methods. Despite this simplicity, meteorological situations are well identified when near-ground atmospheric parameters are analyzed. The LWT presents consistent three-dimensional features when the meteorological situation is well-established, but the results cannot be generalized to every LWT and/or time-slot. The LWT chronology seems to be important, as LWTs impact the whole UBL and the residual layer in upper levels of the atmosphere a good part of the day. To improve the consistency of 3D characterization, the classification method could be enriched including indicators that characterize the UBL height or the chronological sequence, but this would make for a loss of the method’s universality due to the difficulty of obtaining these data.

Some future work is planned concerning other applications of this LWT classification. For example several Urban Climate Analysis Maps are being developed from numerical simulations to inform the Urban Climatic map of Toulouse; in addition, the method can be applied to the short range weather forecast to predict UHI intensity and heat stress conditions in order to improve the local climate and risk management plan.

## Supporting information

S1 Appendix Complementary information about the indicators to determine the optimal number of clusters. (DOC)

S1 File Software and test case. (ZIP)

S2 File S2 to S12 Figs LWT from 0 to 10 (a) Mean diurnal cycles of temperature, precipitation, relative humidity, wind force, wind-rose. (b) Mean diurnal cycles of global solar radiation, downward atmospheric radiation and air pressure for Toulouse. Seasonal intra-clusters differences for winter, spring, summer and autumn are presented in grey, light pink, dark pink and brown respectively. S13 and S14 Figs Cluster frequency occurrence in total percentage (left) and per season (right) for the future projections presented in section 3.3. Spring, summer, autumn and winter seasons are in brown, pink, violet and grey respectively. S15 to S25 Figs Surface energy balance per season and per Local Weather Type from the CAPITOUL campaign. S15 corresponds to the seasonal mean. S16 to S25 corresponds to the LWT 0 to the LWT 10 respectively. S26 to S30 Figs Mean Urban Heat Island intensity per season and per Local Weather Type from the CAPITOUL campaign. S26 corresponds to LWT 0 (up) and 1 (bottom); S27 corresponds to LWT 2 (up) and 3 (bottom); S28 corresponds to LWT 4 (up) and 5 (bottom); S29 corresponds to LWT 6 (up) and 7 (bottom); S30 corresponds to LWT 8 (up) and 9 (bottom). S31 to S43 Figs Radio-soundings per Local Weather Type from the CAPITOUL campaign. S31 and S32 corresponds to LWT 0 in winter and autumn; S22 corresponds to LWT 2 in autumn; S34 to S36 corresponds to LWT3 in winter, spring and summer; S37 and S38 corresponds to LWT5 in winter and autumn; S39 corresponds to LWT 7 in summer; S40 corresponds to LWT 8 in summer, S41 to S43 corresponds to LWT 9 in winter, spring and summer respectively.
(ZIP)
