# Supplementary material for: On the use of local weather types classification to improve climate understanding: An application on the urban climate of Toulouse
Source: PLoS One. 2018 Dec 12;13(12):e0208138. doi: 10.1371/journal.pone.0208138 (PMC6291111; doi:10.1371/journal.pone.0208138)
Supplement: S1 Appendix — (DOC) [file pone.0208138.s001.doc]

**S1 Appendix. Complementary information about the indicators to determine the optimal number of clusters.**

The method used to determine the optimal number of clusters is crucial. This suppelemnt material aims to give complementary information about the indicators that can be used and justify choices done in this paper.

In [22], in addition to the *multivariate Root Mean Square Error* a second indicator was used to fit specific needs of the study, *the attribution frequency deviation*. In one part of the future projections of climate used by [22] the specific humidity (*q*) was not available and the relative humidity (*Hu*) was used in the cluster attribution giving rise to an imperfect attribution. This indicator was used to evaluate the resulting reconstructed series and represented how well the resulting *table of centroids characteristics,* using *Hu* instead *q*, served to identify and attribute the proper cluster number for a particular day of a data series, in this case for the *construction period*. In others words, for the *construction period*, the frequency distribution of clusters issue from this imperfect re attribution process was evaluated against those obtained directly from the k-means clustering method.

For standar application, the original *table of centroids* (k-means) or *dissimilarity matrix (for PAM)* is used in the cluster attribution so this attribution perfectly fits the original and *the attribution frequency deviation* indicator does not make sense.

Complementary work was recently done to explore the pertinence to include other criteria allowing to verify that the intra-class variance is small, i.e. the data belonging to the same class are very close to one another and that the inter-class variance is strong, i.e. the data belonging to different classes are as different as possible. The first criteria is defined as the smallest difference between a point and the barycenter of its cluster and the same point and the barycenter of another cluster. A value is obtained by cluster and this criterion is therefore directly related to the inter-class variance. The second criteria, called *internal diameter*, corresponds to the minimum value of the cluster diameter. The diameter of a cluster is defined as the maximum distance between two observations of the same cluster. This criterion is therefore directly related to the intra-class variance. Unsuccessful results were obtained including or even using those two indicators in an isolated way. We conclude therefore that the RMSE criteria proposed here is good enough to fix an optimal number of clusters in view of this kind of application and is here recommended.
