# Supplementary figures and images for: On the use of local weather types classification to improve climate understanding: An application on the urban climate of Toulouse
Source: PLoS One. 2018 Dec 12;13(12):e0208138. doi: 10.1371/journal.pone.0208138 (PMC6291111; doi:10.1371/journal.pone.0208138)

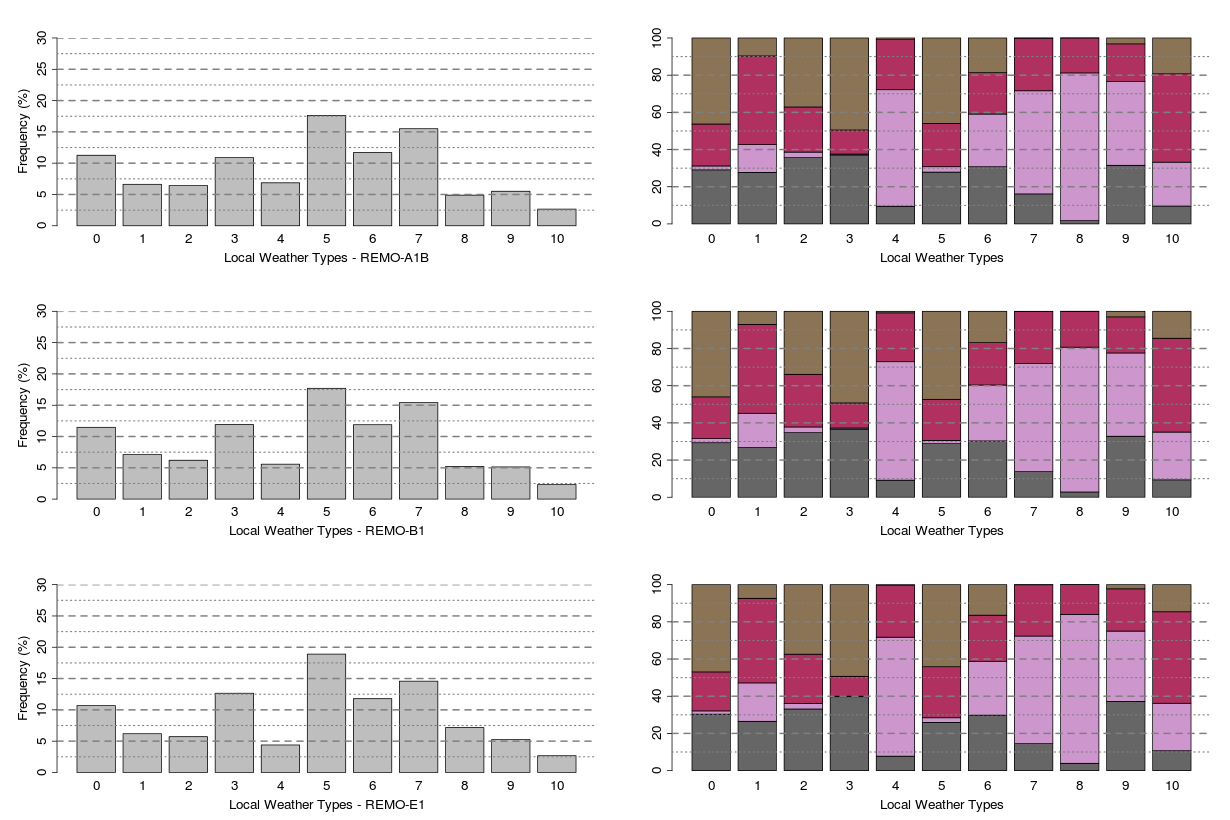

Supplement: S2 File — S2 to S12 Figs LWT from 0 to 10 (a) Mean diurnal cycles of temperature, precipitation, relative humidity, wind force, wind-rose. (b) Mean diurnal cycles of global solar radiation, downward atmospheric radiation and air pressure for Toulouse. Seasonal intra-clusters differences for winter, spring, summer and autumn are presented in grey, light pink, dark pink and brown respectively. S13 and S14 Figs Cluster frequency occurrence in total percentage (left) and per season (right) for the future projections presented in section 3.3. Spring, summer, autumn and winter seasons are in brown, pink, violet and grey respectively. S15 to S25 Figs Surface energy balance per season and per Local Weather Type from the CAPITOUL campaign. S15 corresponds to the seasonal mean. S16 to S25 corresponds to the LWT 0 to the LWT 10 respectively. S26 to S30 Figs Mean Urban Heat Island intensity per season and per Local Weather Type from the CAPITOUL campaign. S26 corresponds to LWT 0 (up) and 1 (bottom); S27 corresponds to LWT 2 (up) and 3 (bottom); S28 corresponds to LWT 4 (up) and 5 (bottom); S29 corresponds to LWT 6 (up) and 7 (bottom); S30 corresponds to LWT 8 (up) and 9 (bottom). S31 to S43 Figs Radio-soundings per Local Weather Type from the CAPITOUL campaign. S31 and S32 corresponds to LWT 0 in winter and autumn; S22 corresponds to LWT 2 in autumn; S34 to S36 corresponds to LWT3 in winter, spring and summer; S37 and S38 corresponds to LWT5 in winter and autumn; S39 corresponds to LWT 7 in summer; S40 corresponds to LWT 8 in summer, S41 to S43 corresponds to LWT 9 in winter, spring and summer respectively. (ZIP) [file pone.0208138.s003.zip › Supporting_Information/S13_Fig.tiff]

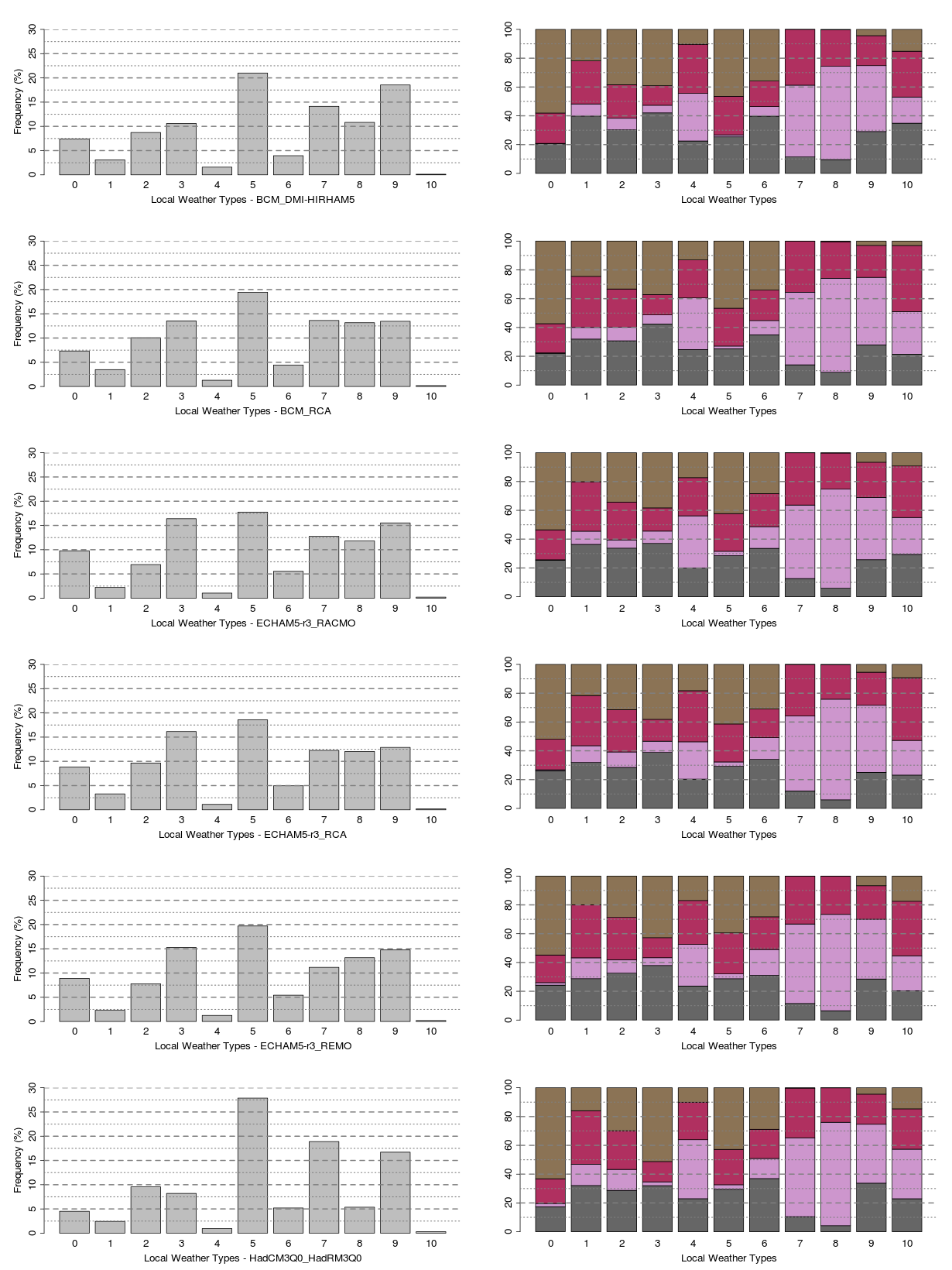

Supplement: S2 File — S2 to S12 Figs LWT from 0 to 10 (a) Mean diurnal cycles of temperature, precipitation, relative humidity, wind force, wind-rose. (b) Mean diurnal cycles of global solar radiation, downward atmospheric radiation and air pressure for Toulouse. Seasonal intra-clusters differences for winter, spring, summer and autumn are presented in grey, light pink, dark pink and brown respectively. S13 and S14 Figs Cluster frequency occurrence in total percentage (left) and per season (right) for the future projections presented in section 3.3. Spring, summer, autumn and winter seasons are in brown, pink, violet and grey respectively. S15 to S25 Figs Surface energy balance per season and per Local Weather Type from the CAPITOUL campaign. S15 corresponds to the seasonal mean. S16 to S25 corresponds to the LWT 0 to the LWT 10 respectively. S26 to S30 Figs Mean Urban Heat Island intensity per season and per Local Weather Type from the CAPITOUL campaign. S26 corresponds to LWT 0 (up) and 1 (bottom); S27 corresponds to LWT 2 (up) and 3 (bottom); S28 corresponds to LWT 4 (up) and 5 (bottom); S29 corresponds to LWT 6 (up) and 7 (bottom); S30 corresponds to LWT 8 (up) and 9 (bottom). S31 to S43 Figs Radio-soundings per Local Weather Type from the CAPITOUL campaign. S31 and S32 corresponds to LWT 0 in winter and autumn; S22 corresponds to LWT 2 in autumn; S34 to S36 corresponds to LWT3 in winter, spring and summer; S37 and S38 corresponds to LWT5 in winter and autumn; S39 corresponds to LWT 7 in summer; S40 corresponds to LWT 8 in summer, S41 to S43 corresponds to LWT 9 in winter, spring and summer respectively. (ZIP) [file pone.0208138.s003.zip › Supporting_Information/S14_Fig.tiff]

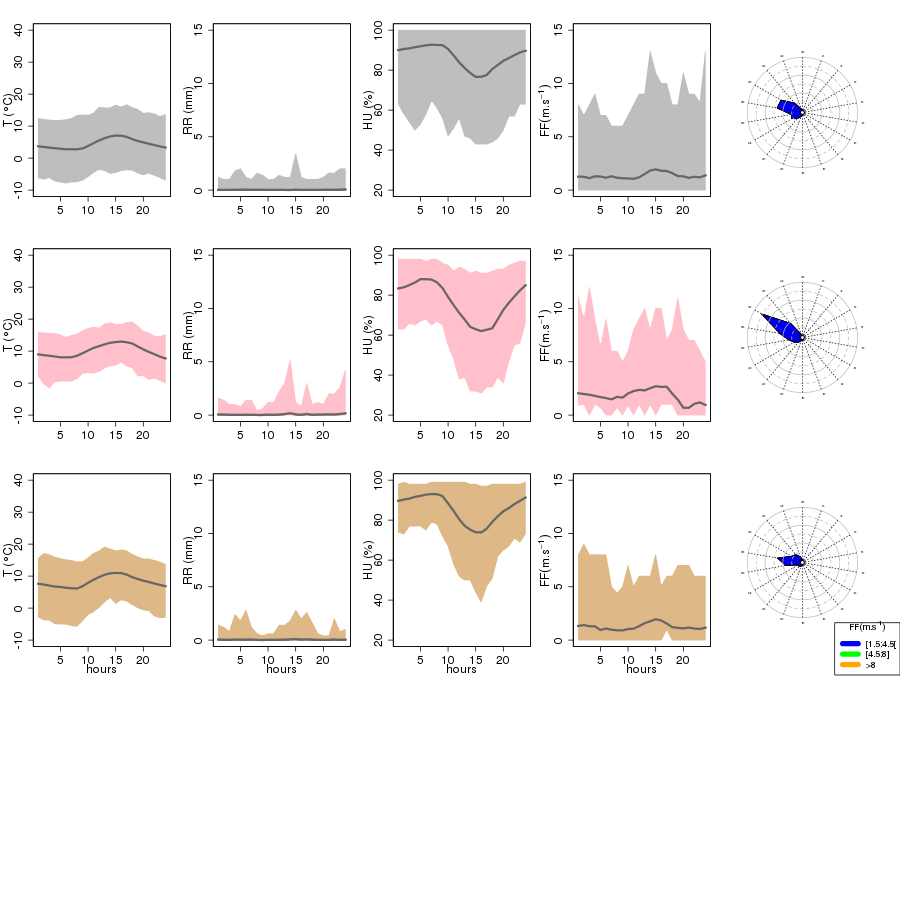

Supplement: S2 File — S2 to S12 Figs LWT from 0 to 10 (a) Mean diurnal cycles of temperature, precipitation, relative humidity, wind force, wind-rose. (b) Mean diurnal cycles of global solar radiation, downward atmospheric radiation and air pressure for Toulouse. Seasonal intra-clusters differences for winter, spring, summer and autumn are presented in grey, light pink, dark pink and brown respectively. S13 and S14 Figs Cluster frequency occurrence in total percentage (left) and per season (right) for the future projections presented in section 3.3. Spring, summer, autumn and winter seasons are in brown, pink, violet and grey respectively. S15 to S25 Figs Surface energy balance per season and per Local Weather Type from the CAPITOUL campaign. S15 corresponds to the seasonal mean. S16 to S25 corresponds to the LWT 0 to the LWT 10 respectively. S26 to S30 Figs Mean Urban Heat Island intensity per season and per Local Weather Type from the CAPITOUL campaign. S26 corresponds to LWT 0 (up) and 1 (bottom); S27 corresponds to LWT 2 (up) and 3 (bottom); S28 corresponds to LWT 4 (up) and 5 (bottom); S29 corresponds to LWT 6 (up) and 7 (bottom); S30 corresponds to LWT 8 (up) and 9 (bottom). S31 to S43 Figs Radio-soundings per Local Weather Type from the CAPITOUL campaign. S31 and S32 corresponds to LWT 0 in winter and autumn; S22 corresponds to LWT 2 in autumn; S34 to S36 corresponds to LWT3 in winter, spring and summer; S37 and S38 corresponds to LWT5 in winter and autumn; S39 corresponds to LWT 7 in summer; S40 corresponds to LWT 8 in summer, S41 to S43 corresponds to LWT 9 in winter, spring and summer respectively. (ZIP) [file pone.0208138.s003.zip › Supporting_Information/S2_Fig_a.tiff]

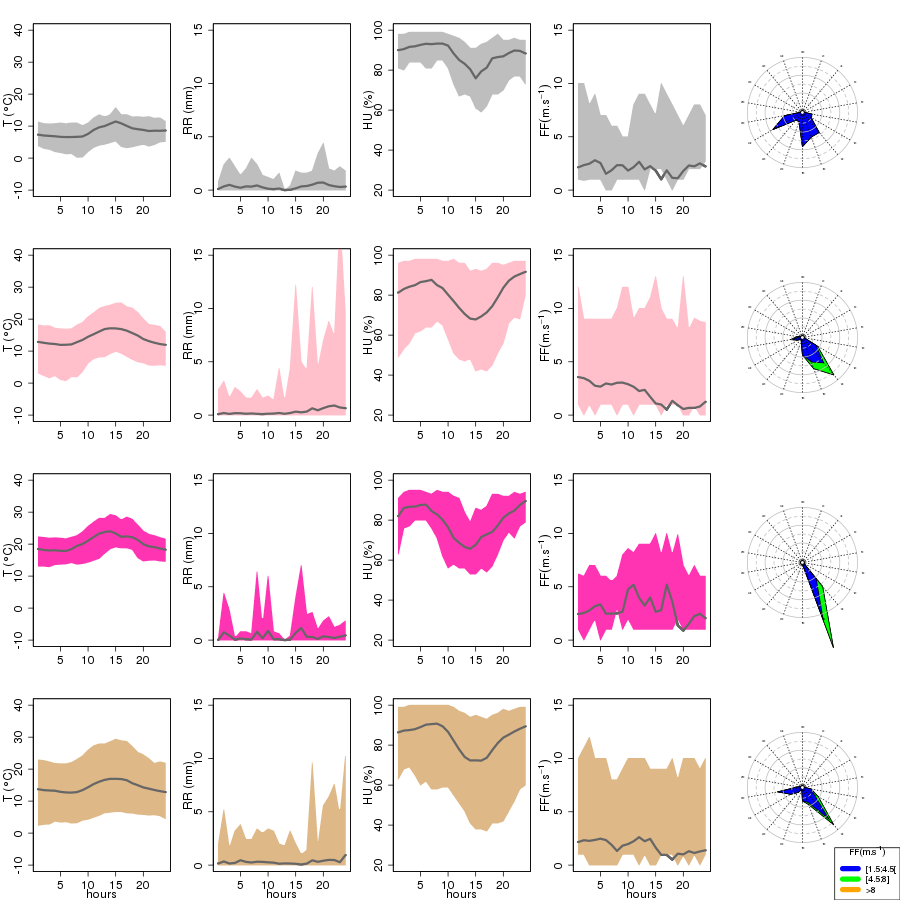

Supplement: S2 File — S2 to S12 Figs LWT from 0 to 10 (a) Mean diurnal cycles of temperature, precipitation, relative humidity, wind force, wind-rose. (b) Mean diurnal cycles of global solar radiation, downward atmospheric radiation and air pressure for Toulouse. Seasonal intra-clusters differences for winter, spring, summer and autumn are presented in grey, light pink, dark pink and brown respectively. S13 and S14 Figs Cluster frequency occurrence in total percentage (left) and per season (right) for the future projections presented in section 3.3. Spring, summer, autumn and winter seasons are in brown, pink, violet and grey respectively. S15 to S25 Figs Surface energy balance per season and per Local Weather Type from the CAPITOUL campaign. S15 corresponds to the seasonal mean. S16 to S25 corresponds to the LWT 0 to the LWT 10 respectively. S26 to S30 Figs Mean Urban Heat Island intensity per season and per Local Weather Type from the CAPITOUL campaign. S26 corresponds to LWT 0 (up) and 1 (bottom); S27 corresponds to LWT 2 (up) and 3 (bottom); S28 corresponds to LWT 4 (up) and 5 (bottom); S29 corresponds to LWT 6 (up) and 7 (bottom); S30 corresponds to LWT 8 (up) and 9 (bottom). S31 to S43 Figs Radio-soundings per Local Weather Type from the CAPITOUL campaign. S31 and S32 corresponds to LWT 0 in winter and autumn; S22 corresponds to LWT 2 in autumn; S34 to S36 corresponds to LWT3 in winter, spring and summer; S37 and S38 corresponds to LWT5 in winter and autumn; S39 corresponds to LWT 7 in summer; S40 corresponds to LWT 8 in summer, S41 to S43 corresponds to LWT 9 in winter, spring and summer respectively. (ZIP) [file pone.0208138.s003.zip › Supporting_Information/S3_Fig_a.tiff]

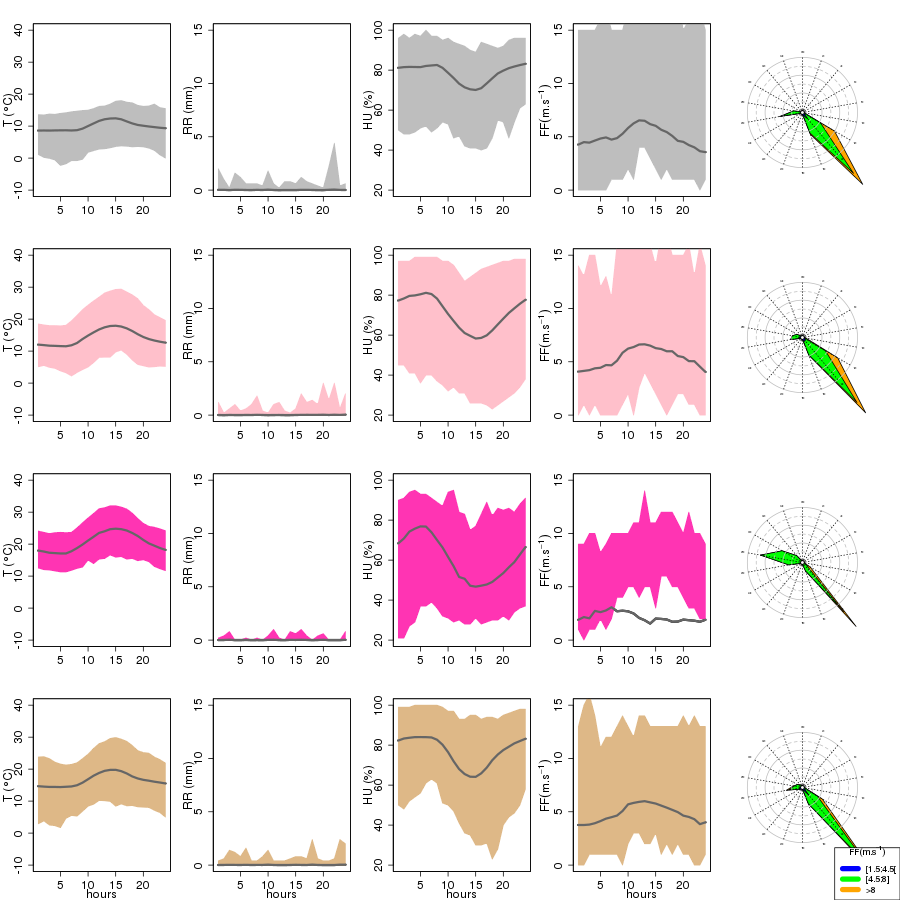

Supplement: S2 File — S2 to S12 Figs LWT from 0 to 10 (a) Mean diurnal cycles of temperature, precipitation, relative humidity, wind force, wind-rose. (b) Mean diurnal cycles of global solar radiation, downward atmospheric radiation and air pressure for Toulouse. Seasonal intra-clusters differences for winter, spring, summer and autumn are presented in grey, light pink, dark pink and brown respectively. S13 and S14 Figs Cluster frequency occurrence in total percentage (left) and per season (right) for the future projections presented in section 3.3. Spring, summer, autumn and winter seasons are in brown, pink, violet and grey respectively. S15 to S25 Figs Surface energy balance per season and per Local Weather Type from the CAPITOUL campaign. S15 corresponds to the seasonal mean. S16 to S25 corresponds to the LWT 0 to the LWT 10 respectively. S26 to S30 Figs Mean Urban Heat Island intensity per season and per Local Weather Type from the CAPITOUL campaign. S26 corresponds to LWT 0 (up) and 1 (bottom); S27 corresponds to LWT 2 (up) and 3 (bottom); S28 corresponds to LWT 4 (up) and 5 (bottom); S29 corresponds to LWT 6 (up) and 7 (bottom); S30 corresponds to LWT 8 (up) and 9 (bottom). S31 to S43 Figs Radio-soundings per Local Weather Type from the CAPITOUL campaign. S31 and S32 corresponds to LWT 0 in winter and autumn; S22 corresponds to LWT 2 in autumn; S34 to S36 corresponds to LWT3 in winter, spring and summer; S37 and S38 corresponds to LWT5 in winter and autumn; S39 corresponds to LWT 7 in summer; S40 corresponds to LWT 8 in summer, S41 to S43 corresponds to LWT 9 in winter, spring and summer respectively. (ZIP) [file pone.0208138.s003.zip › Supporting_Information/S4_Fig_a.tiff]

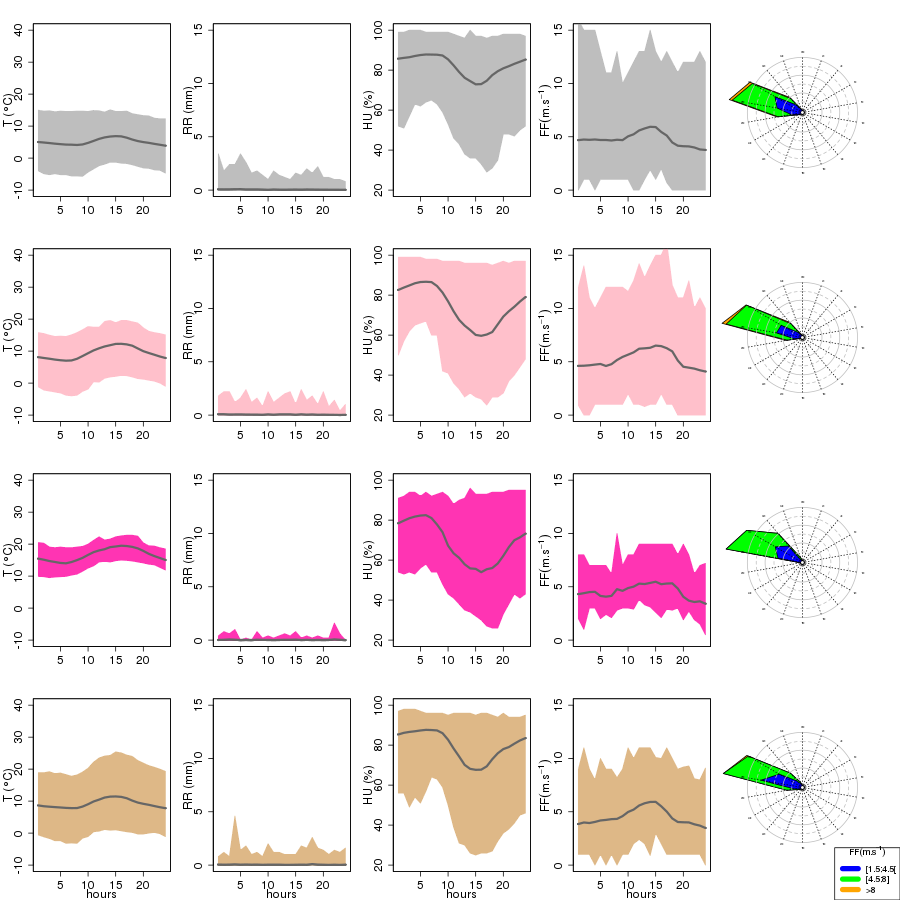

Supplement: S2 File — S2 to S12 Figs LWT from 0 to 10 (a) Mean diurnal cycles of temperature, precipitation, relative humidity, wind force, wind-rose. (b) Mean diurnal cycles of global solar radiation, downward atmospheric radiation and air pressure for Toulouse. Seasonal intra-clusters differences for winter, spring, summer and autumn are presented in grey, light pink, dark pink and brown respectively. S13 and S14 Figs Cluster frequency occurrence in total percentage (left) and per season (right) for the future projections presented in section 3.3. Spring, summer, autumn and winter seasons are in brown, pink, violet and grey respectively. S15 to S25 Figs Surface energy balance per season and per Local Weather Type from the CAPITOUL campaign. S15 corresponds to the seasonal mean. S16 to S25 corresponds to the LWT 0 to the LWT 10 respectively. S26 to S30 Figs Mean Urban Heat Island intensity per season and per Local Weather Type from the CAPITOUL campaign. S26 corresponds to LWT 0 (up) and 1 (bottom); S27 corresponds to LWT 2 (up) and 3 (bottom); S28 corresponds to LWT 4 (up) and 5 (bottom); S29 corresponds to LWT 6 (up) and 7 (bottom); S30 corresponds to LWT 8 (up) and 9 (bottom). S31 to S43 Figs Radio-soundings per Local Weather Type from the CAPITOUL campaign. S31 and S32 corresponds to LWT 0 in winter and autumn; S22 corresponds to LWT 2 in autumn; S34 to S36 corresponds to LWT3 in winter, spring and summer; S37 and S38 corresponds to LWT5 in winter and autumn; S39 corresponds to LWT 7 in summer; S40 corresponds to LWT 8 in summer, S41 to S43 corresponds to LWT 9 in winter, spring and summer respectively. (ZIP) [file pone.0208138.s003.zip › Supporting_Information/S5_Fig_a.tiff]

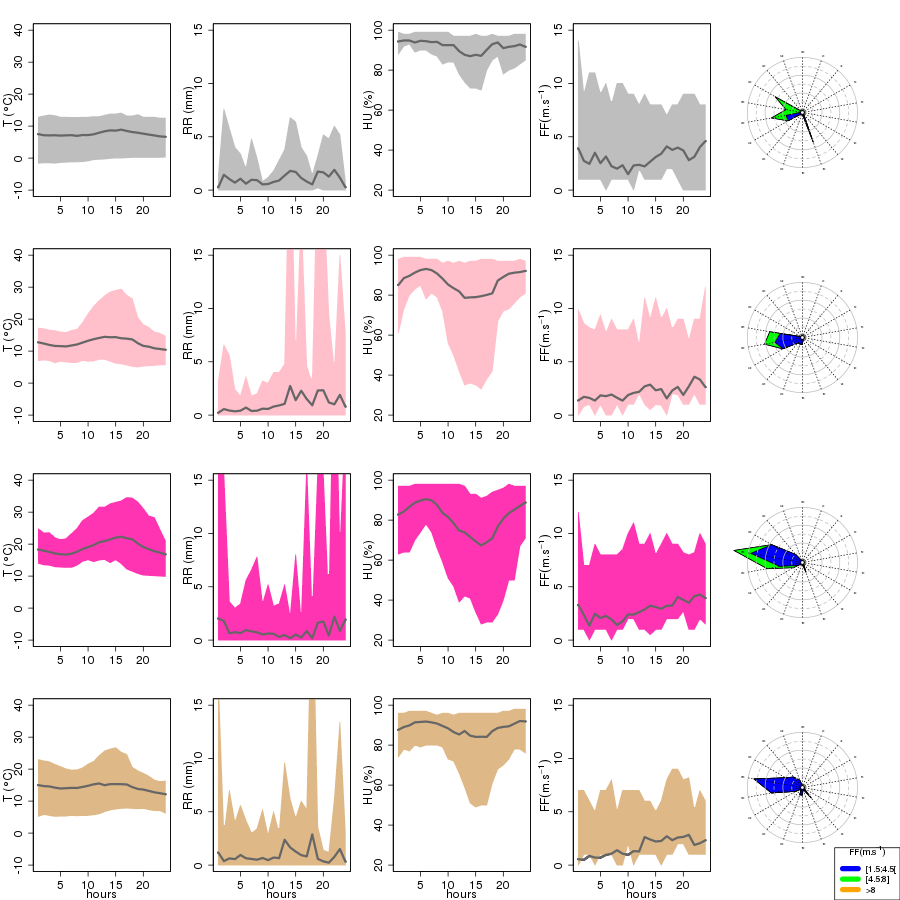

Supplement: S2 File — S2 to S12 Figs LWT from 0 to 10 (a) Mean diurnal cycles of temperature, precipitation, relative humidity, wind force, wind-rose. (b) Mean diurnal cycles of global solar radiation, downward atmospheric radiation and air pressure for Toulouse. Seasonal intra-clusters differences for winter, spring, summer and autumn are presented in grey, light pink, dark pink and brown respectively. S13 and S14 Figs Cluster frequency occurrence in total percentage (left) and per season (right) for the future projections presented in section 3.3. Spring, summer, autumn and winter seasons are in brown, pink, violet and grey respectively. S15 to S25 Figs Surface energy balance per season and per Local Weather Type from the CAPITOUL campaign. S15 corresponds to the seasonal mean. S16 to S25 corresponds to the LWT 0 to the LWT 10 respectively. S26 to S30 Figs Mean Urban Heat Island intensity per season and per Local Weather Type from the CAPITOUL campaign. S26 corresponds to LWT 0 (up) and 1 (bottom); S27 corresponds to LWT 2 (up) and 3 (bottom); S28 corresponds to LWT 4 (up) and 5 (bottom); S29 corresponds to LWT 6 (up) and 7 (bottom); S30 corresponds to LWT 8 (up) and 9 (bottom). S31 to S43 Figs Radio-soundings per Local Weather Type from the CAPITOUL campaign. S31 and S32 corresponds to LWT 0 in winter and autumn; S22 corresponds to LWT 2 in autumn; S34 to S36 corresponds to LWT3 in winter, spring and summer; S37 and S38 corresponds to LWT5 in winter and autumn; S39 corresponds to LWT 7 in summer; S40 corresponds to LWT 8 in summer, S41 to S43 corresponds to LWT 9 in winter, spring and summer respectively. (ZIP) [file pone.0208138.s003.zip › Supporting_Information/S6_Fig_a.tiff]

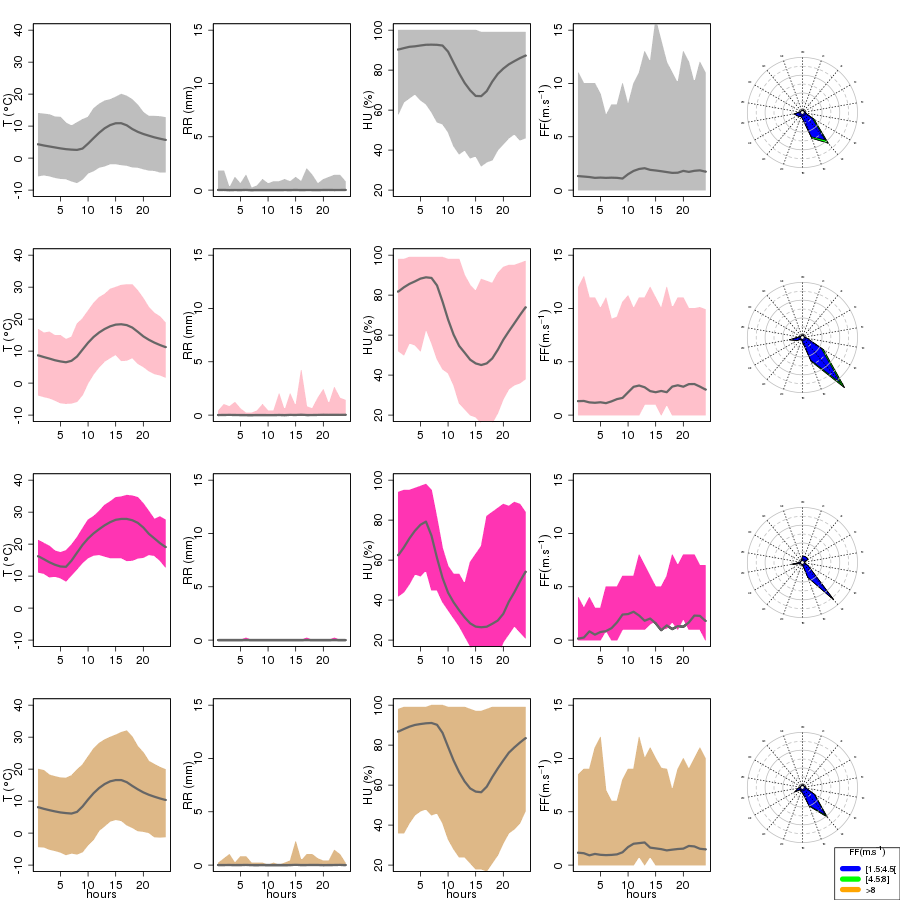

Supplement: S2 File — S2 to S12 Figs LWT from 0 to 10 (a) Mean diurnal cycles of temperature, precipitation, relative humidity, wind force, wind-rose. (b) Mean diurnal cycles of global solar radiation, downward atmospheric radiation and air pressure for Toulouse. Seasonal intra-clusters differences for winter, spring, summer and autumn are presented in grey, light pink, dark pink and brown respectively. S13 and S14 Figs Cluster frequency occurrence in total percentage (left) and per season (right) for the future projections presented in section 3.3. Spring, summer, autumn and winter seasons are in brown, pink, violet and grey respectively. S15 to S25 Figs Surface energy balance per season and per Local Weather Type from the CAPITOUL campaign. S15 corresponds to the seasonal mean. S16 to S25 corresponds to the LWT 0 to the LWT 10 respectively. S26 to S30 Figs Mean Urban Heat Island intensity per season and per Local Weather Type from the CAPITOUL campaign. S26 corresponds to LWT 0 (up) and 1 (bottom); S27 corresponds to LWT 2 (up) and 3 (bottom); S28 corresponds to LWT 4 (up) and 5 (bottom); S29 corresponds to LWT 6 (up) and 7 (bottom); S30 corresponds to LWT 8 (up) and 9 (bottom). S31 to S43 Figs Radio-soundings per Local Weather Type from the CAPITOUL campaign. S31 and S32 corresponds to LWT 0 in winter and autumn; S22 corresponds to LWT 2 in autumn; S34 to S36 corresponds to LWT3 in winter, spring and summer; S37 and S38 corresponds to LWT5 in winter and autumn; S39 corresponds to LWT 7 in summer; S40 corresponds to LWT 8 in summer, S41 to S43 corresponds to LWT 9 in winter, spring and summer respectively. (ZIP) [file pone.0208138.s003.zip › Supporting_Information/S7_Fig_a.tiff]

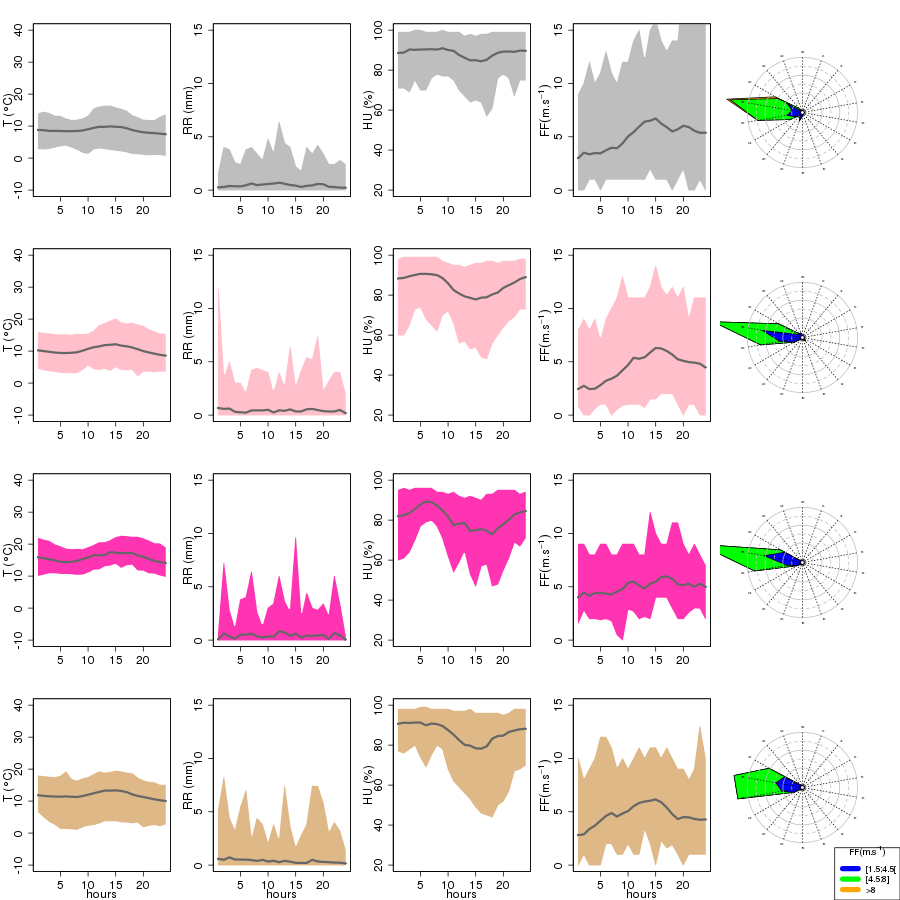

Supplement: S2 File — S2 to S12 Figs LWT from 0 to 10 (a) Mean diurnal cycles of temperature, precipitation, relative humidity, wind force, wind-rose. (b) Mean diurnal cycles of global solar radiation, downward atmospheric radiation and air pressure for Toulouse. Seasonal intra-clusters differences for winter, spring, summer and autumn are presented in grey, light pink, dark pink and brown respectively. S13 and S14 Figs Cluster frequency occurrence in total percentage (left) and per season (right) for the future projections presented in section 3.3. Spring, summer, autumn and winter seasons are in brown, pink, violet and grey respectively. S15 to S25 Figs Surface energy balance per season and per Local Weather Type from the CAPITOUL campaign. S15 corresponds to the seasonal mean. S16 to S25 corresponds to the LWT 0 to the LWT 10 respectively. S26 to S30 Figs Mean Urban Heat Island intensity per season and per Local Weather Type from the CAPITOUL campaign. S26 corresponds to LWT 0 (up) and 1 (bottom); S27 corresponds to LWT 2 (up) and 3 (bottom); S28 corresponds to LWT 4 (up) and 5 (bottom); S29 corresponds to LWT 6 (up) and 7 (bottom); S30 corresponds to LWT 8 (up) and 9 (bottom). S31 to S43 Figs Radio-soundings per Local Weather Type from the CAPITOUL campaign. S31 and S32 corresponds to LWT 0 in winter and autumn; S22 corresponds to LWT 2 in autumn; S34 to S36 corresponds to LWT3 in winter, spring and summer; S37 and S38 corresponds to LWT5 in winter and autumn; S39 corresponds to LWT 7 in summer; S40 corresponds to LWT 8 in summer, S41 to S43 corresponds to LWT 9 in winter, spring and summer respectively. (ZIP) [file pone.0208138.s003.zip › Supporting_Information/S8_Fig_a.tiff]

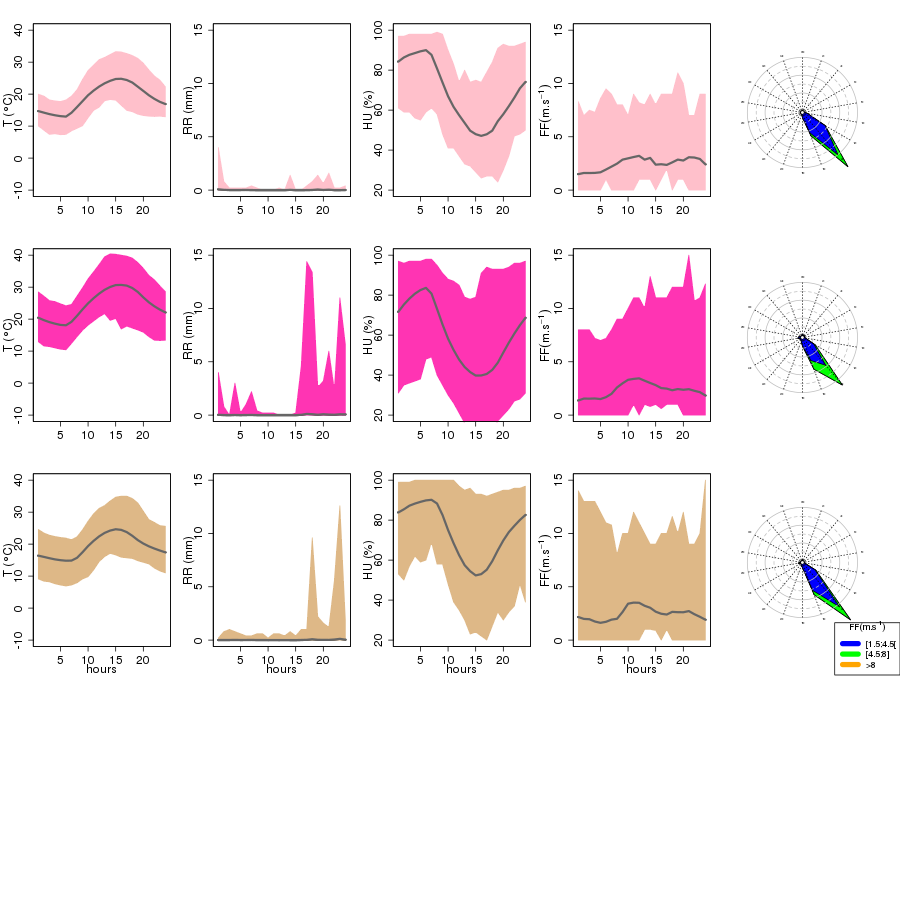

Supplement: S2 File — S2 to S12 Figs LWT from 0 to 10 (a) Mean diurnal cycles of temperature, precipitation, relative humidity, wind force, wind-rose. (b) Mean diurnal cycles of global solar radiation, downward atmospheric radiation and air pressure for Toulouse. Seasonal intra-clusters differences for winter, spring, summer and autumn are presented in grey, light pink, dark pink and brown respectively. S13 and S14 Figs Cluster frequency occurrence in total percentage (left) and per season (right) for the future projections presented in section 3.3. Spring, summer, autumn and winter seasons are in brown, pink, violet and grey respectively. S15 to S25 Figs Surface energy balance per season and per Local Weather Type from the CAPITOUL campaign. S15 corresponds to the seasonal mean. S16 to S25 corresponds to the LWT 0 to the LWT 10 respectively. S26 to S30 Figs Mean Urban Heat Island intensity per season and per Local Weather Type from the CAPITOUL campaign. S26 corresponds to LWT 0 (up) and 1 (bottom); S27 corresponds to LWT 2 (up) and 3 (bottom); S28 corresponds to LWT 4 (up) and 5 (bottom); S29 corresponds to LWT 6 (up) and 7 (bottom); S30 corresponds to LWT 8 (up) and 9 (bottom). S31 to S43 Figs Radio-soundings per Local Weather Type from the CAPITOUL campaign. S31 and S32 corresponds to LWT 0 in winter and autumn; S22 corresponds to LWT 2 in autumn; S34 to S36 corresponds to LWT3 in winter, spring and summer; S37 and S38 corresponds to LWT5 in winter and autumn; S39 corresponds to LWT 7 in summer; S40 corresponds to LWT 8 in summer, S41 to S43 corresponds to LWT 9 in winter, spring and summer respectively. (ZIP) [file pone.0208138.s003.zip › Supporting_Information/S9_Fig_a.tiff]

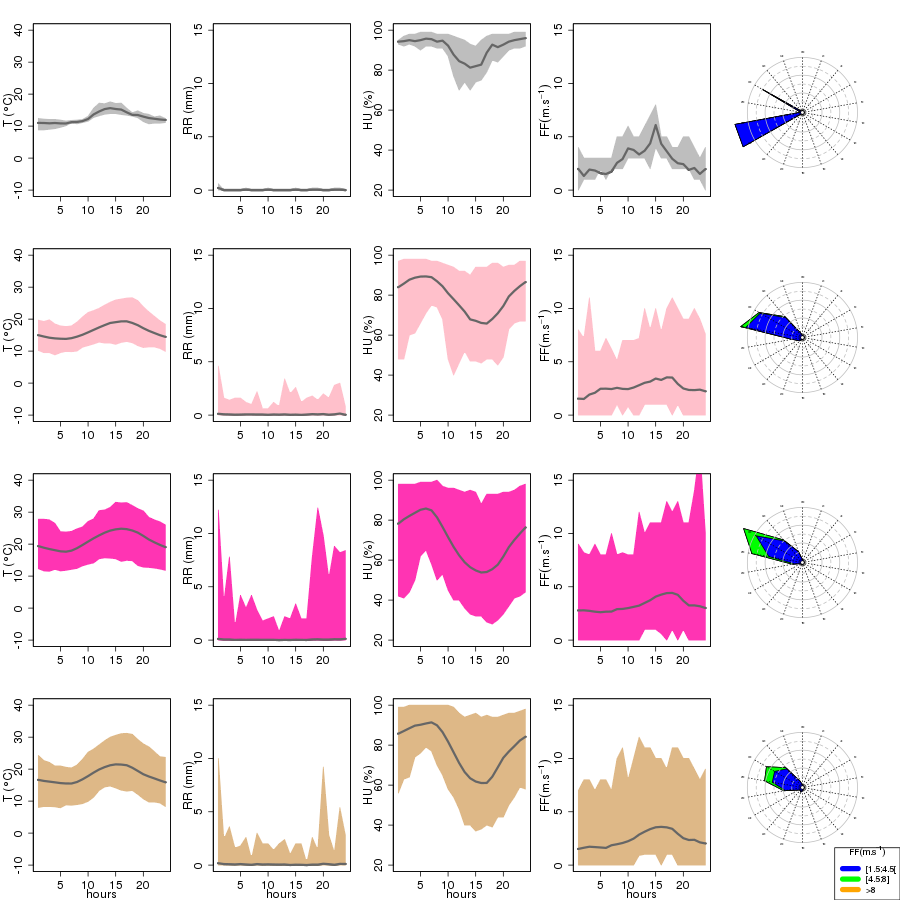

Supplement: S2 File — S2 to S12 Figs LWT from 0 to 10 (a) Mean diurnal cycles of temperature, precipitation, relative humidity, wind force, wind-rose. (b) Mean diurnal cycles of global solar radiation, downward atmospheric radiation and air pressure for Toulouse. Seasonal intra-clusters differences for winter, spring, summer and autumn are presented in grey, light pink, dark pink and brown respectively. S13 and S14 Figs Cluster frequency occurrence in total percentage (left) and per season (right) for the future projections presented in section 3.3. Spring, summer, autumn and winter seasons are in brown, pink, violet and grey respectively. S15 to S25 Figs Surface energy balance per season and per Local Weather Type from the CAPITOUL campaign. S15 corresponds to the seasonal mean. S16 to S25 corresponds to the LWT 0 to the LWT 10 respectively. S26 to S30 Figs Mean Urban Heat Island intensity per season and per Local Weather Type from the CAPITOUL campaign. S26 corresponds to LWT 0 (up) and 1 (bottom); S27 corresponds to LWT 2 (up) and 3 (bottom); S28 corresponds to LWT 4 (up) and 5 (bottom); S29 corresponds to LWT 6 (up) and 7 (bottom); S30 corresponds to LWT 8 (up) and 9 (bottom). S31 to S43 Figs Radio-soundings per Local Weather Type from the CAPITOUL campaign. S31 and S32 corresponds to LWT 0 in winter and autumn; S22 corresponds to LWT 2 in autumn; S34 to S36 corresponds to LWT3 in winter, spring and summer; S37 and S38 corresponds to LWT5 in winter and autumn; S39 corresponds to LWT 7 in summer; S40 corresponds to LWT 8 in summer, S41 to S43 corresponds to LWT 9 in winter, spring and summer respectively. (ZIP) [file pone.0208138.s003.zip › Supporting_Information/S10_Fig_a.tiff]

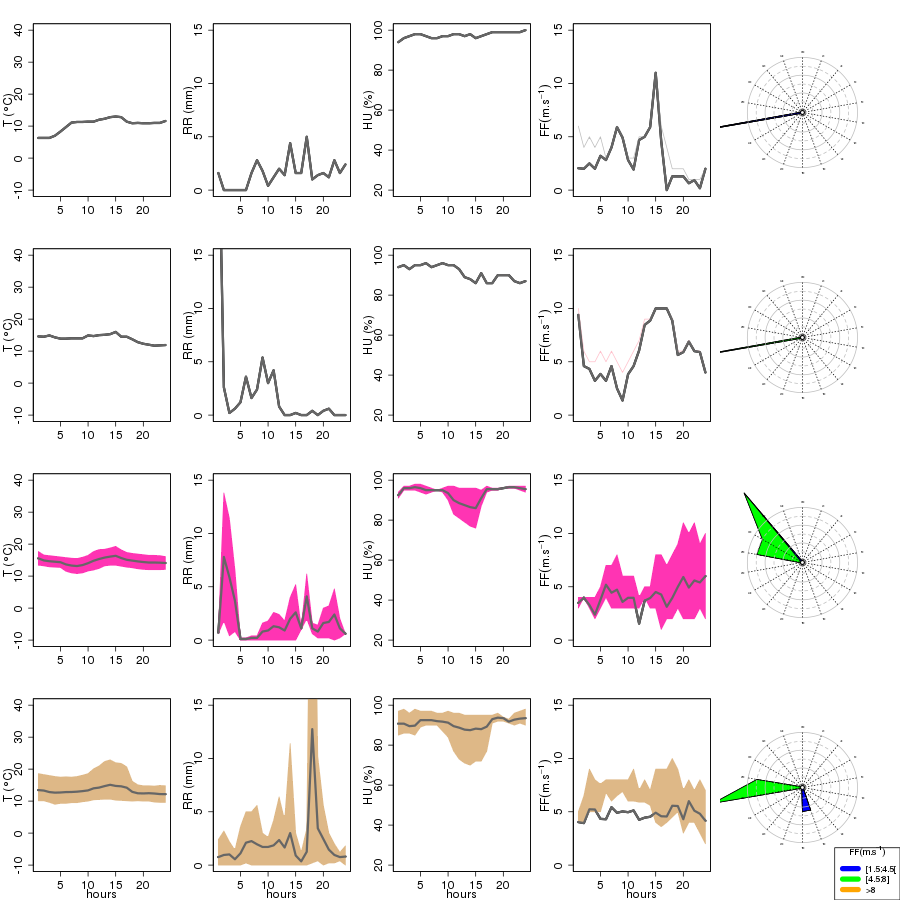

Supplement: S2 File — S2 to S12 Figs LWT from 0 to 10 (a) Mean diurnal cycles of temperature, precipitation, relative humidity, wind force, wind-rose. (b) Mean diurnal cycles of global solar radiation, downward atmospheric radiation and air pressure for Toulouse. Seasonal intra-clusters differences for winter, spring, summer and autumn are presented in grey, light pink, dark pink and brown respectively. S13 and S14 Figs Cluster frequency occurrence in total percentage (left) and per season (right) for the future projections presented in section 3.3. Spring, summer, autumn and winter seasons are in brown, pink, violet and grey respectively. S15 to S25 Figs Surface energy balance per season and per Local Weather Type from the CAPITOUL campaign. S15 corresponds to the seasonal mean. S16 to S25 corresponds to the LWT 0 to the LWT 10 respectively. S26 to S30 Figs Mean Urban Heat Island intensity per season and per Local Weather Type from the CAPITOUL campaign. S26 corresponds to LWT 0 (up) and 1 (bottom); S27 corresponds to LWT 2 (up) and 3 (bottom); S28 corresponds to LWT 4 (up) and 5 (bottom); S29 corresponds to LWT 6 (up) and 7 (bottom); S30 corresponds to LWT 8 (up) and 9 (bottom). S31 to S43 Figs Radio-soundings per Local Weather Type from the CAPITOUL campaign. S31 and S32 corresponds to LWT 0 in winter and autumn; S22 corresponds to LWT 2 in autumn; S34 to S36 corresponds to LWT3 in winter, spring and summer; S37 and S38 corresponds to LWT5 in winter and autumn; S39 corresponds to LWT 7 in summer; S40 corresponds to LWT 8 in summer, S41 to S43 corresponds to LWT 9 in winter, spring and summer respectively. (ZIP) [file pone.0208138.s003.zip › Supporting_Information/S12_Fig_a.tiff]

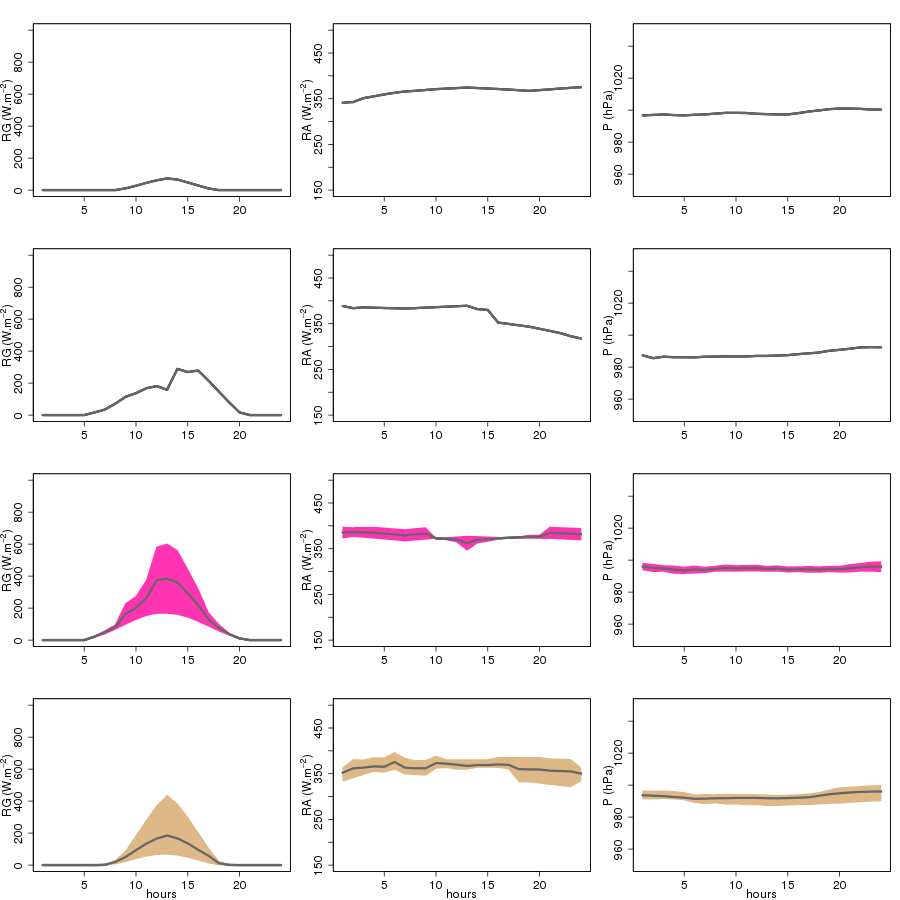

Supplement: S2 File — S2 to S12 Figs LWT from 0 to 10 (a) Mean diurnal cycles of temperature, precipitation, relative humidity, wind force, wind-rose. (b) Mean diurnal cycles of global solar radiation, downward atmospheric radiation and air pressure for Toulouse. Seasonal intra-clusters differences for winter, spring, summer and autumn are presented in grey, light pink, dark pink and brown respectively. S13 and S14 Figs Cluster frequency occurrence in total percentage (left) and per season (right) for the future projections presented in section 3.3. Spring, summer, autumn and winter seasons are in brown, pink, violet and grey respectively. S15 to S25 Figs Surface energy balance per season and per Local Weather Type from the CAPITOUL campaign. S15 corresponds to the seasonal mean. S16 to S25 corresponds to the LWT 0 to the LWT 10 respectively. S26 to S30 Figs Mean Urban Heat Island intensity per season and per Local Weather Type from the CAPITOUL campaign. S26 corresponds to LWT 0 (up) and 1 (bottom); S27 corresponds to LWT 2 (up) and 3 (bottom); S28 corresponds to LWT 4 (up) and 5 (bottom); S29 corresponds to LWT 6 (up) and 7 (bottom); S30 corresponds to LWT 8 (up) and 9 (bottom). S31 to S43 Figs Radio-soundings per Local Weather Type from the CAPITOUL campaign. S31 and S32 corresponds to LWT 0 in winter and autumn; S22 corresponds to LWT 2 in autumn; S34 to S36 corresponds to LWT3 in winter, spring and summer; S37 and S38 corresponds to LWT5 in winter and autumn; S39 corresponds to LWT 7 in summer; S40 corresponds to LWT 8 in summer, S41 to S43 corresponds to LWT 9 in winter, spring and summer respectively. (ZIP) [file pone.0208138.s003.zip › Supporting_Information/S12_Fig_b.tiff]

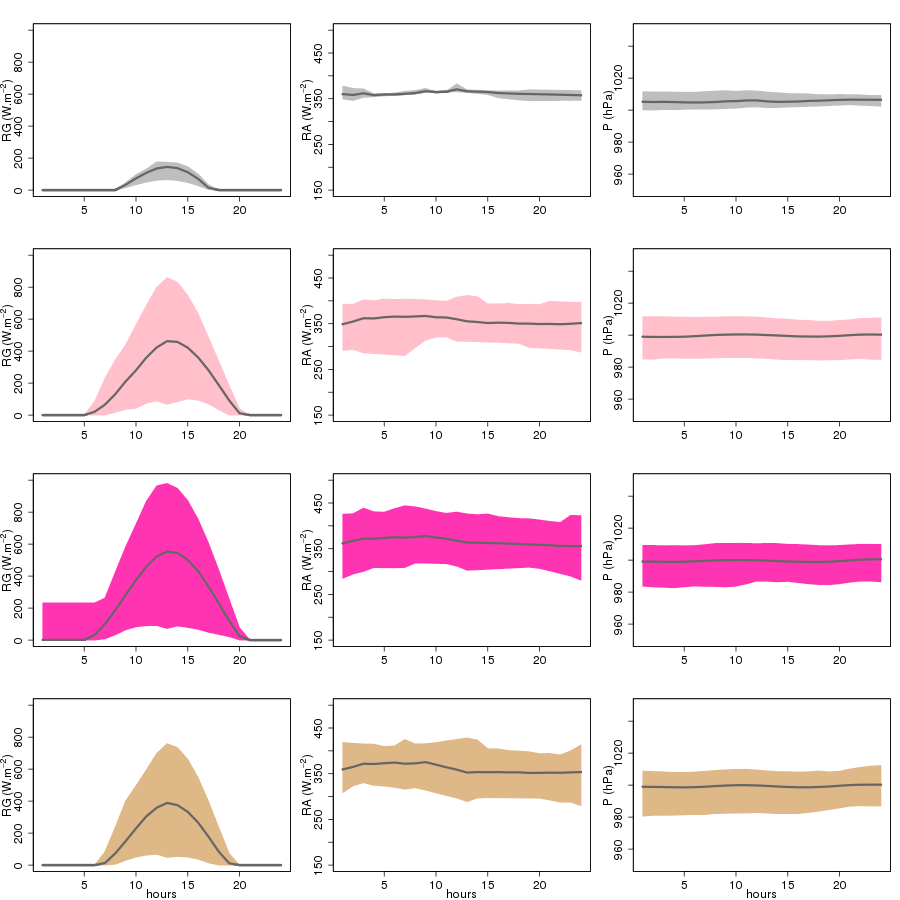

Supplement: S2 File — S2 to S12 Figs LWT from 0 to 10 (a) Mean diurnal cycles of temperature, precipitation, relative humidity, wind force, wind-rose. (b) Mean diurnal cycles of global solar radiation, downward atmospheric radiation and air pressure for Toulouse. Seasonal intra-clusters differences for winter, spring, summer and autumn are presented in grey, light pink, dark pink and brown respectively. S13 and S14 Figs Cluster frequency occurrence in total percentage (left) and per season (right) for the future projections presented in section 3.3. Spring, summer, autumn and winter seasons are in brown, pink, violet and grey respectively. S15 to S25 Figs Surface energy balance per season and per Local Weather Type from the CAPITOUL campaign. S15 corresponds to the seasonal mean. S16 to S25 corresponds to the LWT 0 to the LWT 10 respectively. S26 to S30 Figs Mean Urban Heat Island intensity per season and per Local Weather Type from the CAPITOUL campaign. S26 corresponds to LWT 0 (up) and 1 (bottom); S27 corresponds to LWT 2 (up) and 3 (bottom); S28 corresponds to LWT 4 (up) and 5 (bottom); S29 corresponds to LWT 6 (up) and 7 (bottom); S30 corresponds to LWT 8 (up) and 9 (bottom). S31 to S43 Figs Radio-soundings per Local Weather Type from the CAPITOUL campaign. S31 and S32 corresponds to LWT 0 in winter and autumn; S22 corresponds to LWT 2 in autumn; S34 to S36 corresponds to LWT3 in winter, spring and summer; S37 and S38 corresponds to LWT5 in winter and autumn; S39 corresponds to LWT 7 in summer; S40 corresponds to LWT 8 in summer, S41 to S43 corresponds to LWT 9 in winter, spring and summer respectively. (ZIP) [file pone.0208138.s003.zip › Supporting_Information/S10_Fig_b.tiff]

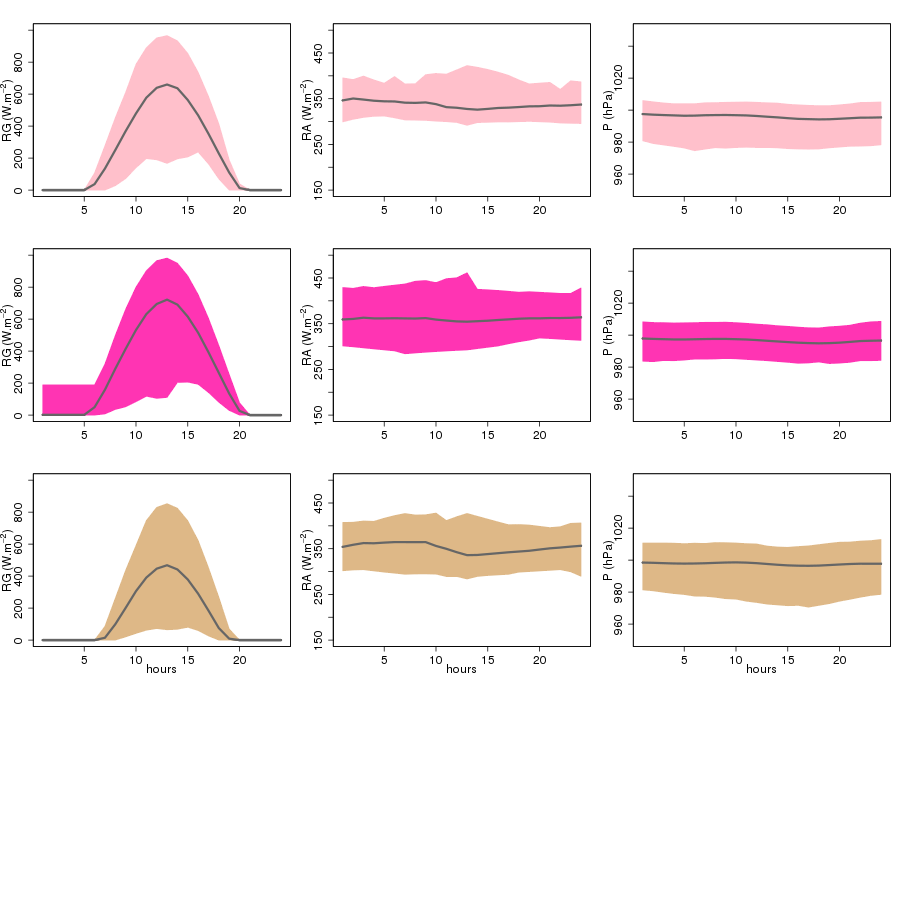

Supplement: S2 File — S2 to S12 Figs LWT from 0 to 10 (a) Mean diurnal cycles of temperature, precipitation, relative humidity, wind force, wind-rose. (b) Mean diurnal cycles of global solar radiation, downward atmospheric radiation and air pressure for Toulouse. Seasonal intra-clusters differences for winter, spring, summer and autumn are presented in grey, light pink, dark pink and brown respectively. S13 and S14 Figs Cluster frequency occurrence in total percentage (left) and per season (right) for the future projections presented in section 3.3. Spring, summer, autumn and winter seasons are in brown, pink, violet and grey respectively. S15 to S25 Figs Surface energy balance per season and per Local Weather Type from the CAPITOUL campaign. S15 corresponds to the seasonal mean. S16 to S25 corresponds to the LWT 0 to the LWT 10 respectively. S26 to S30 Figs Mean Urban Heat Island intensity per season and per Local Weather Type from the CAPITOUL campaign. S26 corresponds to LWT 0 (up) and 1 (bottom); S27 corresponds to LWT 2 (up) and 3 (bottom); S28 corresponds to LWT 4 (up) and 5 (bottom); S29 corresponds to LWT 6 (up) and 7 (bottom); S30 corresponds to LWT 8 (up) and 9 (bottom). S31 to S43 Figs Radio-soundings per Local Weather Type from the CAPITOUL campaign. S31 and S32 corresponds to LWT 0 in winter and autumn; S22 corresponds to LWT 2 in autumn; S34 to S36 corresponds to LWT3 in winter, spring and summer; S37 and S38 corresponds to LWT5 in winter and autumn; S39 corresponds to LWT 7 in summer; S40 corresponds to LWT 8 in summer, S41 to S43 corresponds to LWT 9 in winter, spring and summer respectively. (ZIP) [file pone.0208138.s003.zip › Supporting_Information/S9_Fig_b.tiff]

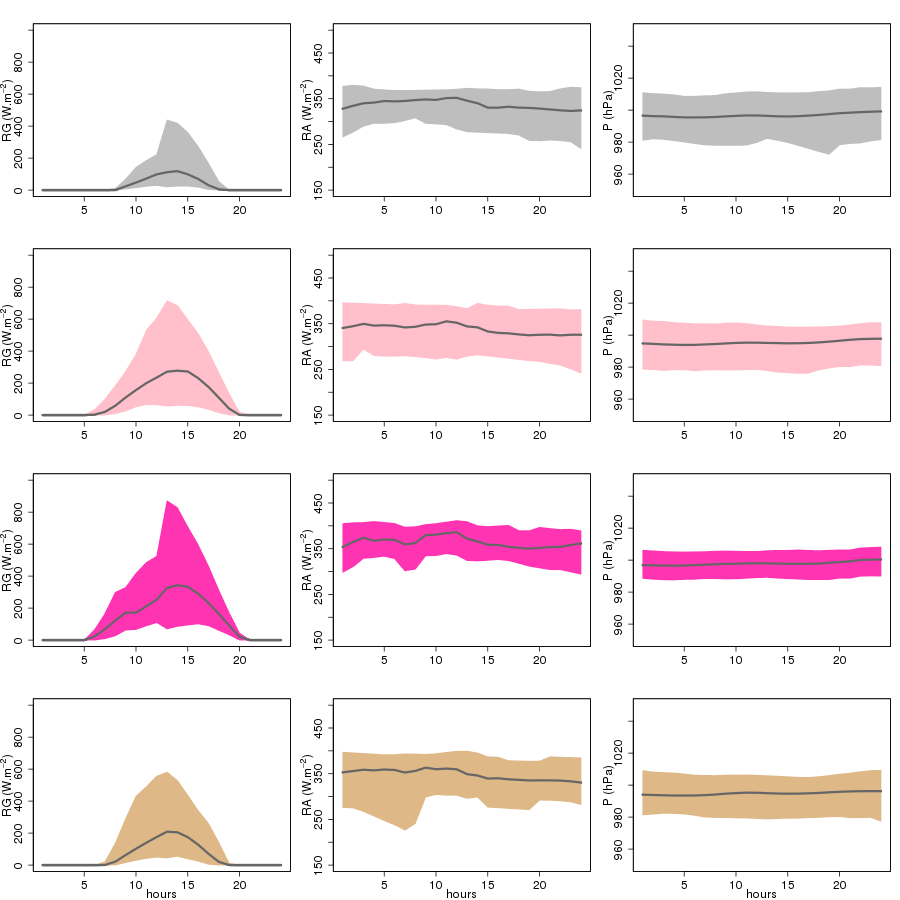

Supplement: S2 File — S2 to S12 Figs LWT from 0 to 10 (a) Mean diurnal cycles of temperature, precipitation, relative humidity, wind force, wind-rose. (b) Mean diurnal cycles of global solar radiation, downward atmospheric radiation and air pressure for Toulouse. Seasonal intra-clusters differences for winter, spring, summer and autumn are presented in grey, light pink, dark pink and brown respectively. S13 and S14 Figs Cluster frequency occurrence in total percentage (left) and per season (right) for the future projections presented in section 3.3. Spring, summer, autumn and winter seasons are in brown, pink, violet and grey respectively. S15 to S25 Figs Surface energy balance per season and per Local Weather Type from the CAPITOUL campaign. S15 corresponds to the seasonal mean. S16 to S25 corresponds to the LWT 0 to the LWT 10 respectively. S26 to S30 Figs Mean Urban Heat Island intensity per season and per Local Weather Type from the CAPITOUL campaign. S26 corresponds to LWT 0 (up) and 1 (bottom); S27 corresponds to LWT 2 (up) and 3 (bottom); S28 corresponds to LWT 4 (up) and 5 (bottom); S29 corresponds to LWT 6 (up) and 7 (bottom); S30 corresponds to LWT 8 (up) and 9 (bottom). S31 to S43 Figs Radio-soundings per Local Weather Type from the CAPITOUL campaign. S31 and S32 corresponds to LWT 0 in winter and autumn; S22 corresponds to LWT 2 in autumn; S34 to S36 corresponds to LWT3 in winter, spring and summer; S37 and S38 corresponds to LWT5 in winter and autumn; S39 corresponds to LWT 7 in summer; S40 corresponds to LWT 8 in summer, S41 to S43 corresponds to LWT 9 in winter, spring and summer respectively. (ZIP) [file pone.0208138.s003.zip › Supporting_Information/S8_Fig_b.tiff]

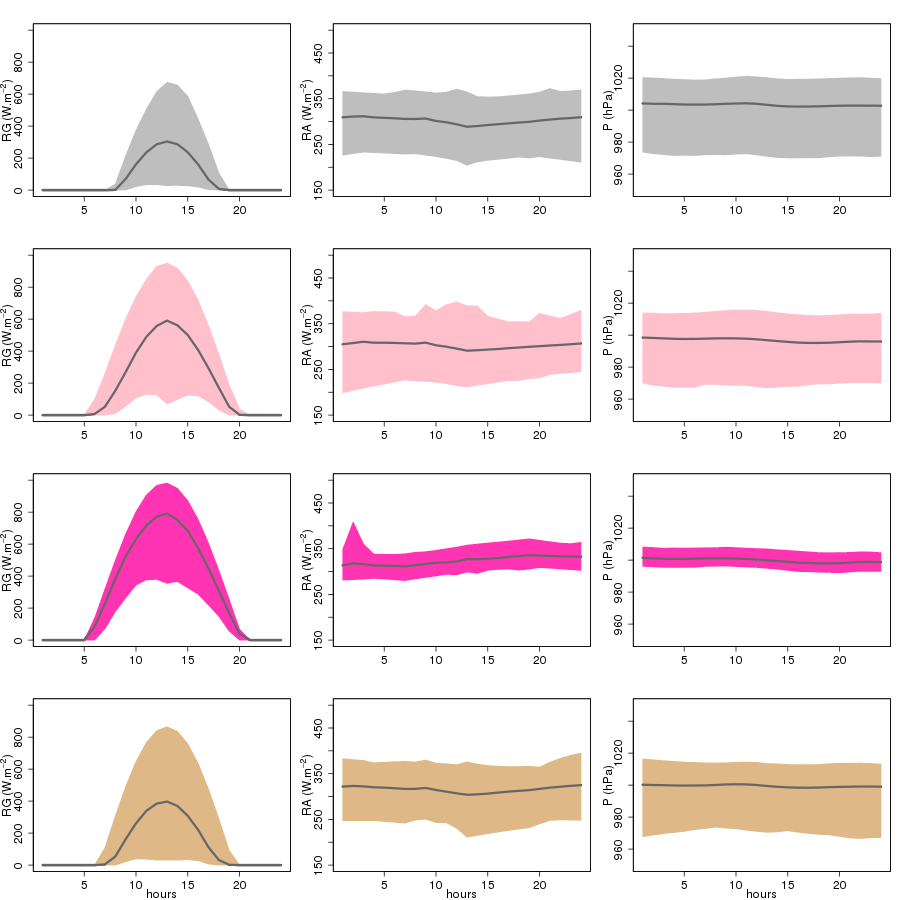

Supplement: S2 File — S2 to S12 Figs LWT from 0 to 10 (a) Mean diurnal cycles of temperature, precipitation, relative humidity, wind force, wind-rose. (b) Mean diurnal cycles of global solar radiation, downward atmospheric radiation and air pressure for Toulouse. Seasonal intra-clusters differences for winter, spring, summer and autumn are presented in grey, light pink, dark pink and brown respectively. S13 and S14 Figs Cluster frequency occurrence in total percentage (left) and per season (right) for the future projections presented in section 3.3. Spring, summer, autumn and winter seasons are in brown, pink, violet and grey respectively. S15 to S25 Figs Surface energy balance per season and per Local Weather Type from the CAPITOUL campaign. S15 corresponds to the seasonal mean. S16 to S25 corresponds to the LWT 0 to the LWT 10 respectively. S26 to S30 Figs Mean Urban Heat Island intensity per season and per Local Weather Type from the CAPITOUL campaign. S26 corresponds to LWT 0 (up) and 1 (bottom); S27 corresponds to LWT 2 (up) and 3 (bottom); S28 corresponds to LWT 4 (up) and 5 (bottom); S29 corresponds to LWT 6 (up) and 7 (bottom); S30 corresponds to LWT 8 (up) and 9 (bottom). S31 to S43 Figs Radio-soundings per Local Weather Type from the CAPITOUL campaign. S31 and S32 corresponds to LWT 0 in winter and autumn; S22 corresponds to LWT 2 in autumn; S34 to S36 corresponds to LWT3 in winter, spring and summer; S37 and S38 corresponds to LWT5 in winter and autumn; S39 corresponds to LWT 7 in summer; S40 corresponds to LWT 8 in summer, S41 to S43 corresponds to LWT 9 in winter, spring and summer respectively. (ZIP) [file pone.0208138.s003.zip › Supporting_Information/S7_Fig_b.tiff]

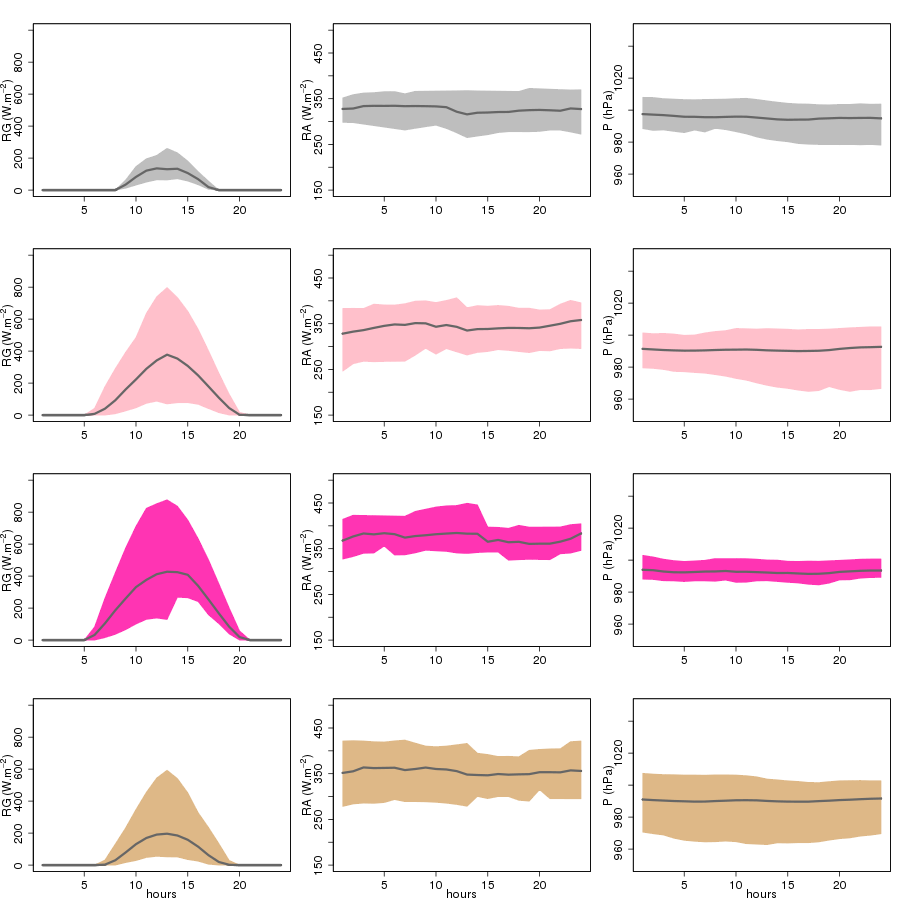

Supplement: S2 File — S2 to S12 Figs LWT from 0 to 10 (a) Mean diurnal cycles of temperature, precipitation, relative humidity, wind force, wind-rose. (b) Mean diurnal cycles of global solar radiation, downward atmospheric radiation and air pressure for Toulouse. Seasonal intra-clusters differences for winter, spring, summer and autumn are presented in grey, light pink, dark pink and brown respectively. S13 and S14 Figs Cluster frequency occurrence in total percentage (left) and per season (right) for the future projections presented in section 3.3. Spring, summer, autumn and winter seasons are in brown, pink, violet and grey respectively. S15 to S25 Figs Surface energy balance per season and per Local Weather Type from the CAPITOUL campaign. S15 corresponds to the seasonal mean. S16 to S25 corresponds to the LWT 0 to the LWT 10 respectively. S26 to S30 Figs Mean Urban Heat Island intensity per season and per Local Weather Type from the CAPITOUL campaign. S26 corresponds to LWT 0 (up) and 1 (bottom); S27 corresponds to LWT 2 (up) and 3 (bottom); S28 corresponds to LWT 4 (up) and 5 (bottom); S29 corresponds to LWT 6 (up) and 7 (bottom); S30 corresponds to LWT 8 (up) and 9 (bottom). S31 to S43 Figs Radio-soundings per Local Weather Type from the CAPITOUL campaign. S31 and S32 corresponds to LWT 0 in winter and autumn; S22 corresponds to LWT 2 in autumn; S34 to S36 corresponds to LWT3 in winter, spring and summer; S37 and S38 corresponds to LWT5 in winter and autumn; S39 corresponds to LWT 7 in summer; S40 corresponds to LWT 8 in summer, S41 to S43 corresponds to LWT 9 in winter, spring and summer respectively. (ZIP) [file pone.0208138.s003.zip › Supporting_Information/S3_Fig_b.tiff]

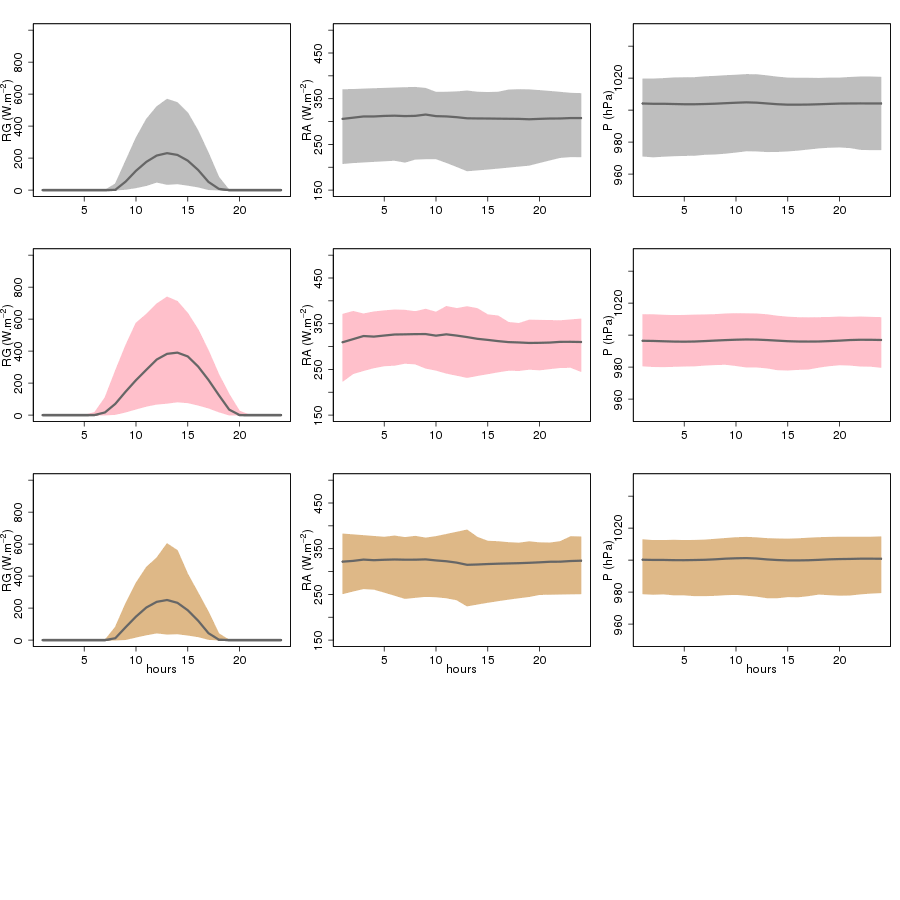

Supplement: S2 File — S2 to S12 Figs LWT from 0 to 10 (a) Mean diurnal cycles of temperature, precipitation, relative humidity, wind force, wind-rose. (b) Mean diurnal cycles of global solar radiation, downward atmospheric radiation and air pressure for Toulouse. Seasonal intra-clusters differences for winter, spring, summer and autumn are presented in grey, light pink, dark pink and brown respectively. S13 and S14 Figs Cluster frequency occurrence in total percentage (left) and per season (right) for the future projections presented in section 3.3. Spring, summer, autumn and winter seasons are in brown, pink, violet and grey respectively. S15 to S25 Figs Surface energy balance per season and per Local Weather Type from the CAPITOUL campaign. S15 corresponds to the seasonal mean. S16 to S25 corresponds to the LWT 0 to the LWT 10 respectively. S26 to S30 Figs Mean Urban Heat Island intensity per season and per Local Weather Type from the CAPITOUL campaign. S26 corresponds to LWT 0 (up) and 1 (bottom); S27 corresponds to LWT 2 (up) and 3 (bottom); S28 corresponds to LWT 4 (up) and 5 (bottom); S29 corresponds to LWT 6 (up) and 7 (bottom); S30 corresponds to LWT 8 (up) and 9 (bottom). S31 to S43 Figs Radio-soundings per Local Weather Type from the CAPITOUL campaign. S31 and S32 corresponds to LWT 0 in winter and autumn; S22 corresponds to LWT 2 in autumn; S34 to S36 corresponds to LWT3 in winter, spring and summer; S37 and S38 corresponds to LWT5 in winter and autumn; S39 corresponds to LWT 7 in summer; S40 corresponds to LWT 8 in summer, S41 to S43 corresponds to LWT 9 in winter, spring and summer respectively. (ZIP) [file pone.0208138.s003.zip › Supporting_Information/S2_Fig_b.tiff]

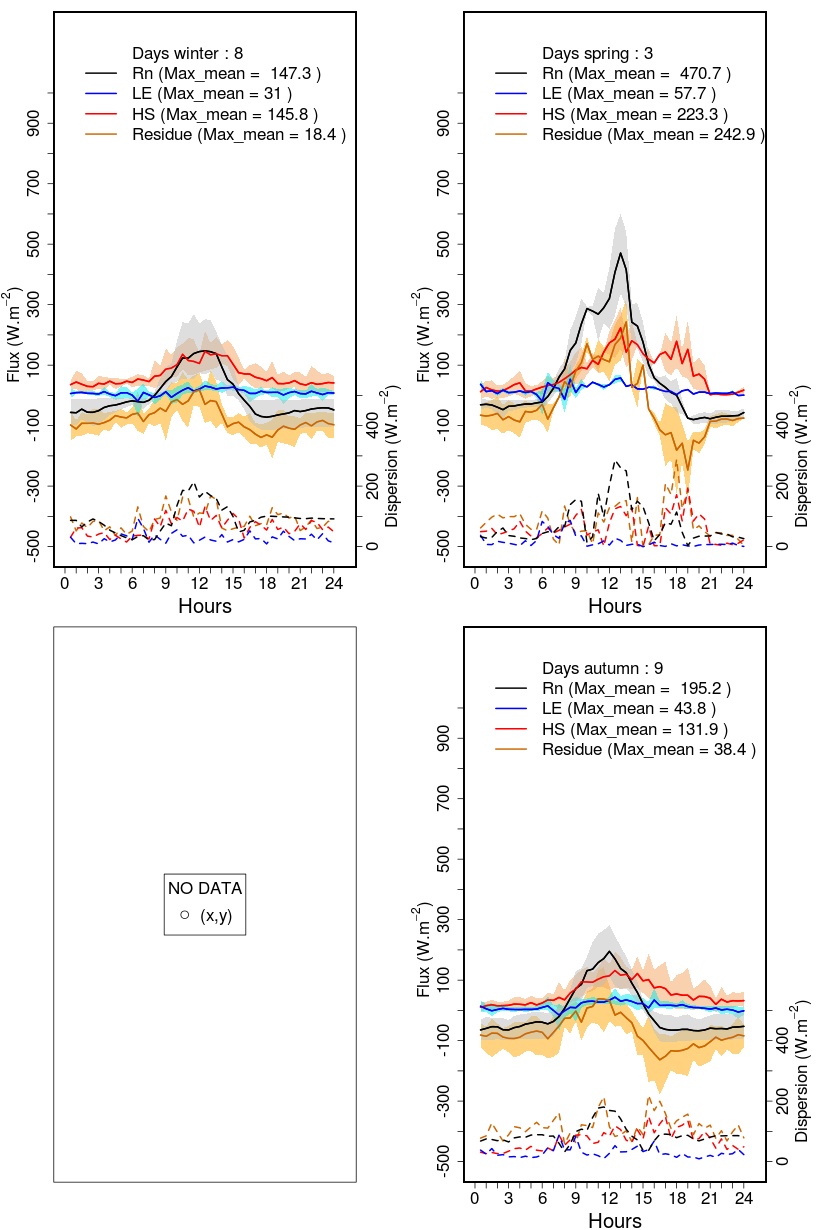

Supplement: S2 File — S2 to S12 Figs LWT from 0 to 10 (a) Mean diurnal cycles of temperature, precipitation, relative humidity, wind force, wind-rose. (b) Mean diurnal cycles of global solar radiation, downward atmospheric radiation and air pressure for Toulouse. Seasonal intra-clusters differences for winter, spring, summer and autumn are presented in grey, light pink, dark pink and brown respectively. S13 and S14 Figs Cluster frequency occurrence in total percentage (left) and per season (right) for the future projections presented in section 3.3. Spring, summer, autumn and winter seasons are in brown, pink, violet and grey respectively. S15 to S25 Figs Surface energy balance per season and per Local Weather Type from the CAPITOUL campaign. S15 corresponds to the seasonal mean. S16 to S25 corresponds to the LWT 0 to the LWT 10 respectively. S26 to S30 Figs Mean Urban Heat Island intensity per season and per Local Weather Type from the CAPITOUL campaign. S26 corresponds to LWT 0 (up) and 1 (bottom); S27 corresponds to LWT 2 (up) and 3 (bottom); S28 corresponds to LWT 4 (up) and 5 (bottom); S29 corresponds to LWT 6 (up) and 7 (bottom); S30 corresponds to LWT 8 (up) and 9 (bottom). S31 to S43 Figs Radio-soundings per Local Weather Type from the CAPITOUL campaign. S31 and S32 corresponds to LWT 0 in winter and autumn; S22 corresponds to LWT 2 in autumn; S34 to S36 corresponds to LWT3 in winter, spring and summer; S37 and S38 corresponds to LWT5 in winter and autumn; S39 corresponds to LWT 7 in summer; S40 corresponds to LWT 8 in summer, S41 to S43 corresponds to LWT 9 in winter, spring and summer respectively. (ZIP) [file pone.0208138.s003.zip › Supporting_Information/S16.tiff]

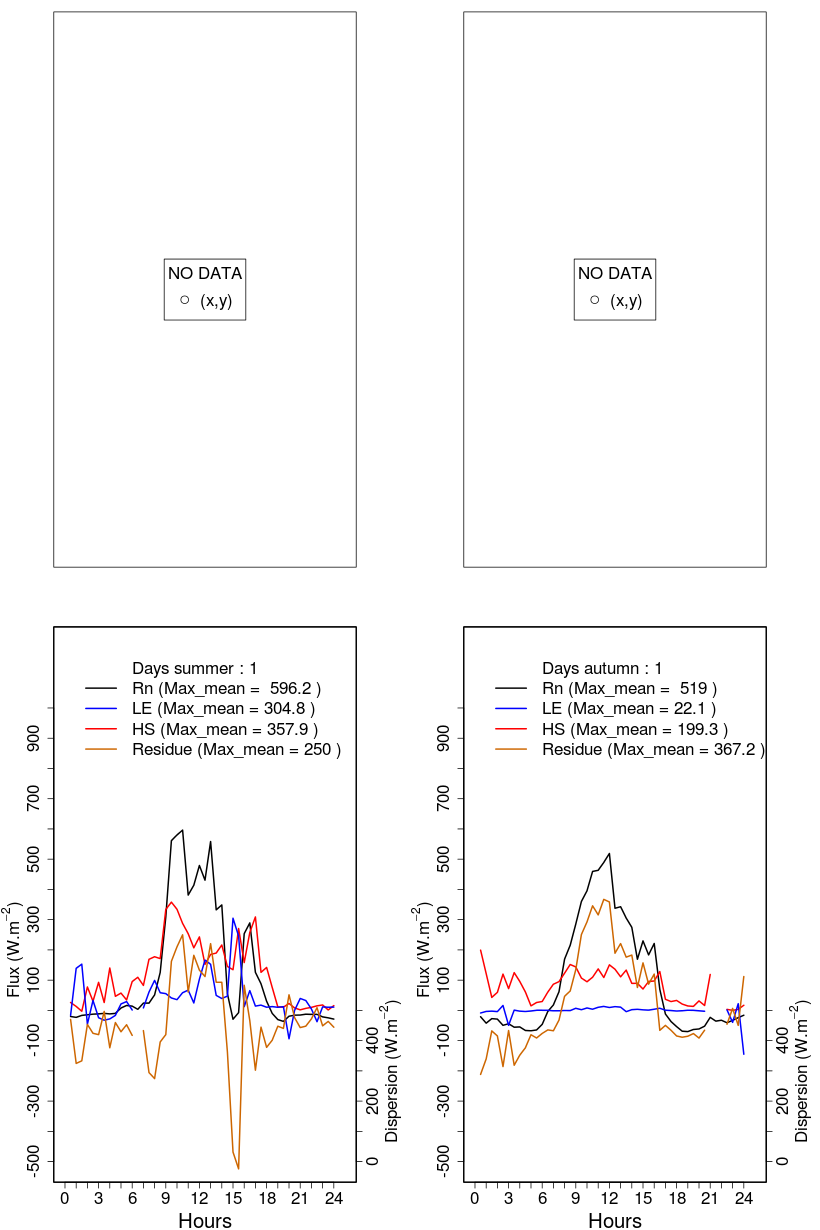

Supplement: S2 File — S2 to S12 Figs LWT from 0 to 10 (a) Mean diurnal cycles of temperature, precipitation, relative humidity, wind force, wind-rose. (b) Mean diurnal cycles of global solar radiation, downward atmospheric radiation and air pressure for Toulouse. Seasonal intra-clusters differences for winter, spring, summer and autumn are presented in grey, light pink, dark pink and brown respectively. S13 and S14 Figs Cluster frequency occurrence in total percentage (left) and per season (right) for the future projections presented in section 3.3. Spring, summer, autumn and winter seasons are in brown, pink, violet and grey respectively. S15 to S25 Figs Surface energy balance per season and per Local Weather Type from the CAPITOUL campaign. S15 corresponds to the seasonal mean. S16 to S25 corresponds to the LWT 0 to the LWT 10 respectively. S26 to S30 Figs Mean Urban Heat Island intensity per season and per Local Weather Type from the CAPITOUL campaign. S26 corresponds to LWT 0 (up) and 1 (bottom); S27 corresponds to LWT 2 (up) and 3 (bottom); S28 corresponds to LWT 4 (up) and 5 (bottom); S29 corresponds to LWT 6 (up) and 7 (bottom); S30 corresponds to LWT 8 (up) and 9 (bottom). S31 to S43 Figs Radio-soundings per Local Weather Type from the CAPITOUL campaign. S31 and S32 corresponds to LWT 0 in winter and autumn; S22 corresponds to LWT 2 in autumn; S34 to S36 corresponds to LWT3 in winter, spring and summer; S37 and S38 corresponds to LWT5 in winter and autumn; S39 corresponds to LWT 7 in summer; S40 corresponds to LWT 8 in summer, S41 to S43 corresponds to LWT 9 in winter, spring and summer respectively. (ZIP) [file pone.0208138.s003.zip › Supporting_Information/S17.tiff]

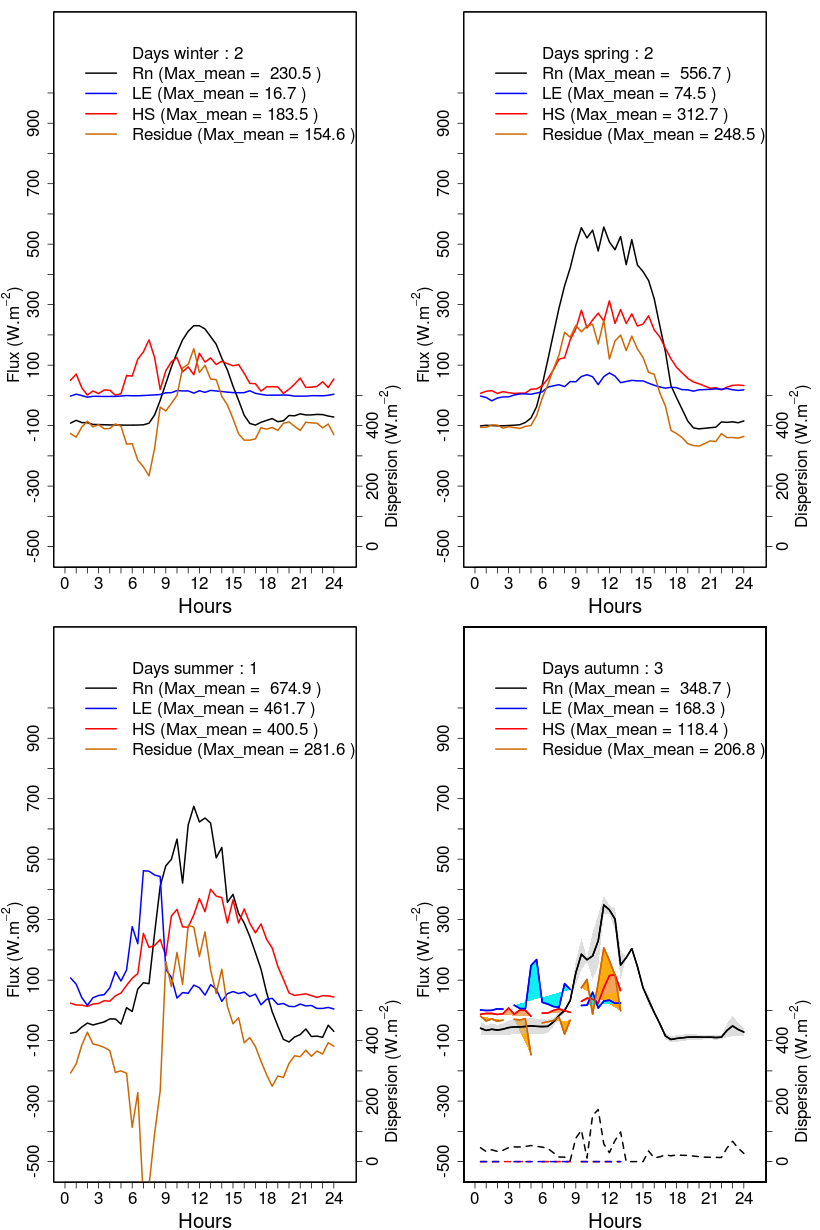

Supplement: S2 File — S2 to S12 Figs LWT from 0 to 10 (a) Mean diurnal cycles of temperature, precipitation, relative humidity, wind force, wind-rose. (b) Mean diurnal cycles of global solar radiation, downward atmospheric radiation and air pressure for Toulouse. Seasonal intra-clusters differences for winter, spring, summer and autumn are presented in grey, light pink, dark pink and brown respectively. S13 and S14 Figs Cluster frequency occurrence in total percentage (left) and per season (right) for the future projections presented in section 3.3. Spring, summer, autumn and winter seasons are in brown, pink, violet and grey respectively. S15 to S25 Figs Surface energy balance per season and per Local Weather Type from the CAPITOUL campaign. S15 corresponds to the seasonal mean. S16 to S25 corresponds to the LWT 0 to the LWT 10 respectively. S26 to S30 Figs Mean Urban Heat Island intensity per season and per Local Weather Type from the CAPITOUL campaign. S26 corresponds to LWT 0 (up) and 1 (bottom); S27 corresponds to LWT 2 (up) and 3 (bottom); S28 corresponds to LWT 4 (up) and 5 (bottom); S29 corresponds to LWT 6 (up) and 7 (bottom); S30 corresponds to LWT 8 (up) and 9 (bottom). S31 to S43 Figs Radio-soundings per Local Weather Type from the CAPITOUL campaign. S31 and S32 corresponds to LWT 0 in winter and autumn; S22 corresponds to LWT 2 in autumn; S34 to S36 corresponds to LWT3 in winter, spring and summer; S37 and S38 corresponds to LWT5 in winter and autumn; S39 corresponds to LWT 7 in summer; S40 corresponds to LWT 8 in summer, S41 to S43 corresponds to LWT 9 in winter, spring and summer respectively. (ZIP) [file pone.0208138.s003.zip › Supporting_Information/S18.tiff]

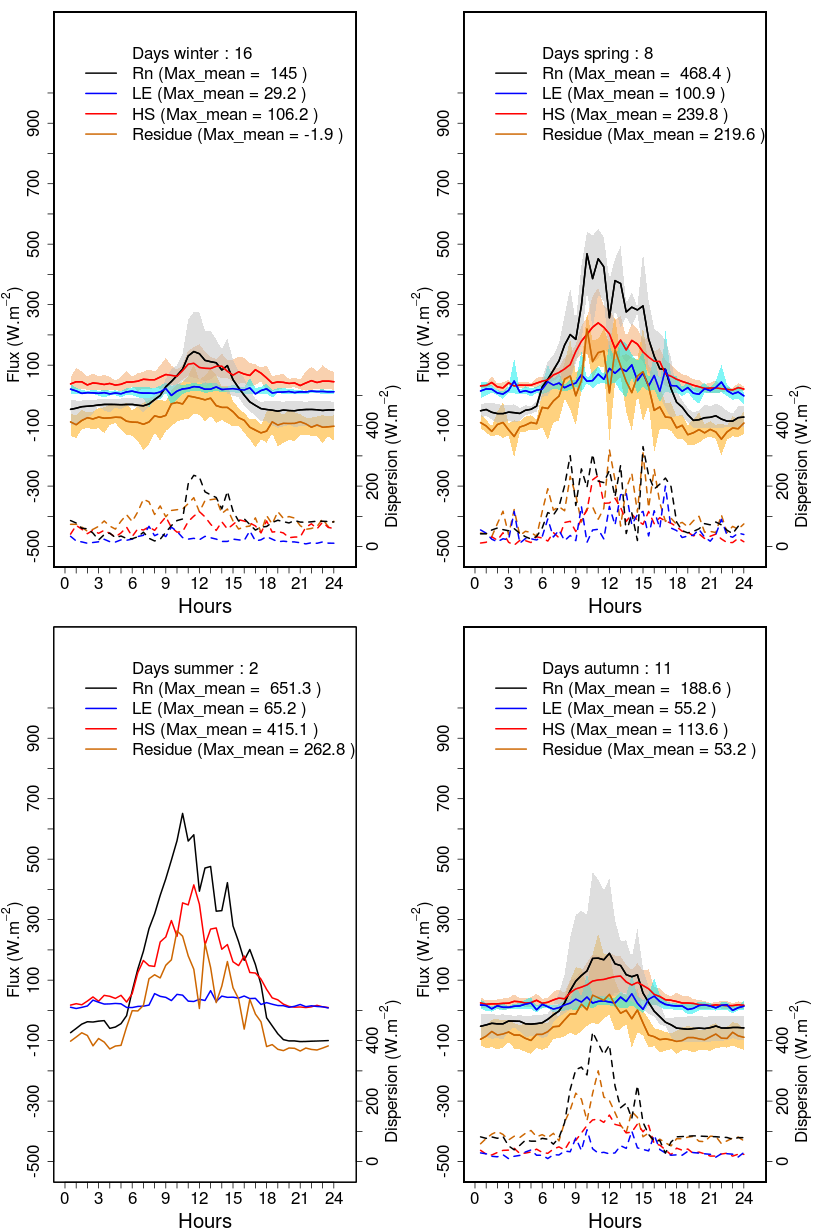

Supplement: S2 File — S2 to S12 Figs LWT from 0 to 10 (a) Mean diurnal cycles of temperature, precipitation, relative humidity, wind force, wind-rose. (b) Mean diurnal cycles of global solar radiation, downward atmospheric radiation and air pressure for Toulouse. Seasonal intra-clusters differences for winter, spring, summer and autumn are presented in grey, light pink, dark pink and brown respectively. S13 and S14 Figs Cluster frequency occurrence in total percentage (left) and per season (right) for the future projections presented in section 3.3. Spring, summer, autumn and winter seasons are in brown, pink, violet and grey respectively. S15 to S25 Figs Surface energy balance per season and per Local Weather Type from the CAPITOUL campaign. S15 corresponds to the seasonal mean. S16 to S25 corresponds to the LWT 0 to the LWT 10 respectively. S26 to S30 Figs Mean Urban Heat Island intensity per season and per Local Weather Type from the CAPITOUL campaign. S26 corresponds to LWT 0 (up) and 1 (bottom); S27 corresponds to LWT 2 (up) and 3 (bottom); S28 corresponds to LWT 4 (up) and 5 (bottom); S29 corresponds to LWT 6 (up) and 7 (bottom); S30 corresponds to LWT 8 (up) and 9 (bottom). S31 to S43 Figs Radio-soundings per Local Weather Type from the CAPITOUL campaign. S31 and S32 corresponds to LWT 0 in winter and autumn; S22 corresponds to LWT 2 in autumn; S34 to S36 corresponds to LWT3 in winter, spring and summer; S37 and S38 corresponds to LWT5 in winter and autumn; S39 corresponds to LWT 7 in summer; S40 corresponds to LWT 8 in summer, S41 to S43 corresponds to LWT 9 in winter, spring and summer respectively. (ZIP) [file pone.0208138.s003.zip › Supporting_Information/S19.tiff]

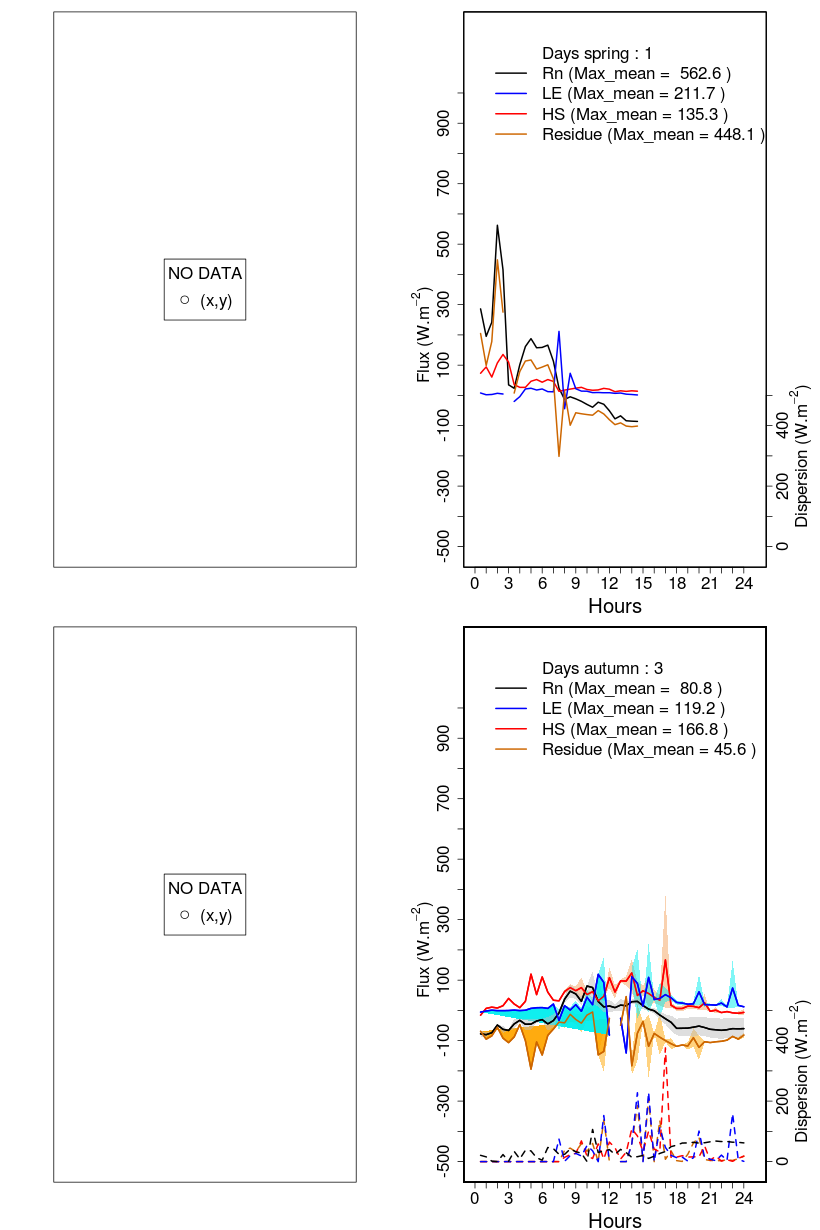

Supplement: S2 File — S2 to S12 Figs LWT from 0 to 10 (a) Mean diurnal cycles of temperature, precipitation, relative humidity, wind force, wind-rose. (b) Mean diurnal cycles of global solar radiation, downward atmospheric radiation and air pressure for Toulouse. Seasonal intra-clusters differences for winter, spring, summer and autumn are presented in grey, light pink, dark pink and brown respectively. S13 and S14 Figs Cluster frequency occurrence in total percentage (left) and per season (right) for the future projections presented in section 3.3. Spring, summer, autumn and winter seasons are in brown, pink, violet and grey respectively. S15 to S25 Figs Surface energy balance per season and per Local Weather Type from the CAPITOUL campaign. S15 corresponds to the seasonal mean. S16 to S25 corresponds to the LWT 0 to the LWT 10 respectively. S26 to S30 Figs Mean Urban Heat Island intensity per season and per Local Weather Type from the CAPITOUL campaign. S26 corresponds to LWT 0 (up) and 1 (bottom); S27 corresponds to LWT 2 (up) and 3 (bottom); S28 corresponds to LWT 4 (up) and 5 (bottom); S29 corresponds to LWT 6 (up) and 7 (bottom); S30 corresponds to LWT 8 (up) and 9 (bottom). S31 to S43 Figs Radio-soundings per Local Weather Type from the CAPITOUL campaign. S31 and S32 corresponds to LWT 0 in winter and autumn; S22 corresponds to LWT 2 in autumn; S34 to S36 corresponds to LWT3 in winter, spring and summer; S37 and S38 corresponds to LWT5 in winter and autumn; S39 corresponds to LWT 7 in summer; S40 corresponds to LWT 8 in summer, S41 to S43 corresponds to LWT 9 in winter, spring and summer respectively. (ZIP) [file pone.0208138.s003.zip › Supporting_Information/S20.tiff]

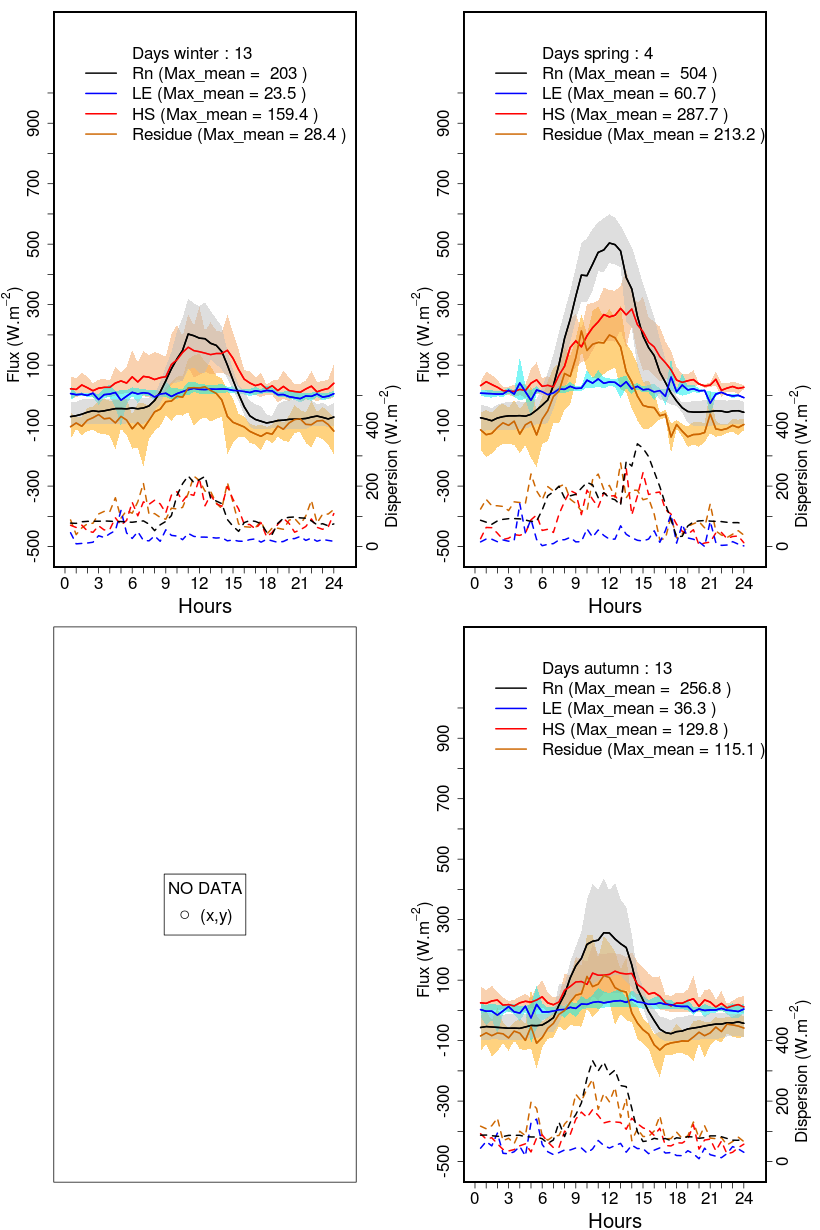

Supplement: S2 File — S2 to S12 Figs LWT from 0 to 10 (a) Mean diurnal cycles of temperature, precipitation, relative humidity, wind force, wind-rose. (b) Mean diurnal cycles of global solar radiation, downward atmospheric radiation and air pressure for Toulouse. Seasonal intra-clusters differences for winter, spring, summer and autumn are presented in grey, light pink, dark pink and brown respectively. S13 and S14 Figs Cluster frequency occurrence in total percentage (left) and per season (right) for the future projections presented in section 3.3. Spring, summer, autumn and winter seasons are in brown, pink, violet and grey respectively. S15 to S25 Figs Surface energy balance per season and per Local Weather Type from the CAPITOUL campaign. S15 corresponds to the seasonal mean. S16 to S25 corresponds to the LWT 0 to the LWT 10 respectively. S26 to S30 Figs Mean Urban Heat Island intensity per season and per Local Weather Type from the CAPITOUL campaign. S26 corresponds to LWT 0 (up) and 1 (bottom); S27 corresponds to LWT 2 (up) and 3 (bottom); S28 corresponds to LWT 4 (up) and 5 (bottom); S29 corresponds to LWT 6 (up) and 7 (bottom); S30 corresponds to LWT 8 (up) and 9 (bottom). S31 to S43 Figs Radio-soundings per Local Weather Type from the CAPITOUL campaign. S31 and S32 corresponds to LWT 0 in winter and autumn; S22 corresponds to LWT 2 in autumn; S34 to S36 corresponds to LWT3 in winter, spring and summer; S37 and S38 corresponds to LWT5 in winter and autumn; S39 corresponds to LWT 7 in summer; S40 corresponds to LWT 8 in summer, S41 to S43 corresponds to LWT 9 in winter, spring and summer respectively. (ZIP) [file pone.0208138.s003.zip › Supporting_Information/S21.tiff]

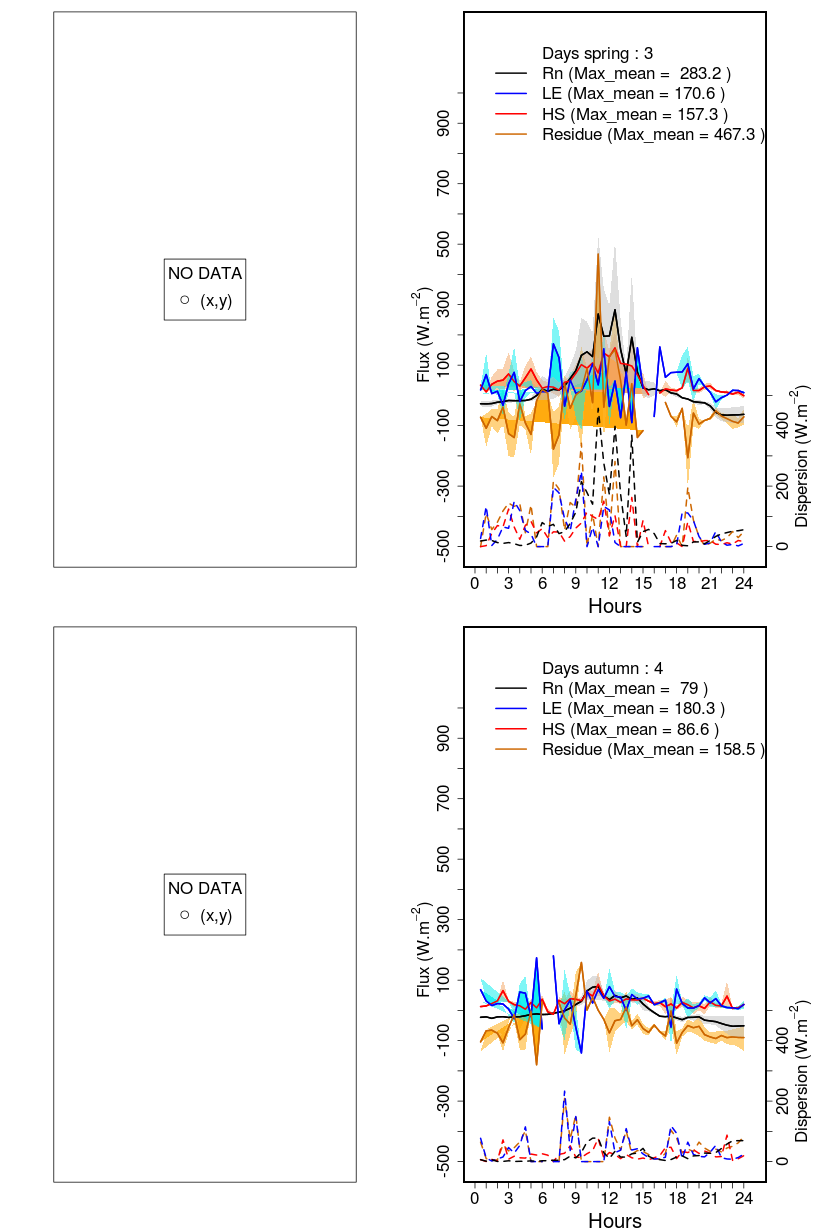

Supplement: S2 File — S2 to S12 Figs LWT from 0 to 10 (a) Mean diurnal cycles of temperature, precipitation, relative humidity, wind force, wind-rose. (b) Mean diurnal cycles of global solar radiation, downward atmospheric radiation and air pressure for Toulouse. Seasonal intra-clusters differences for winter, spring, summer and autumn are presented in grey, light pink, dark pink and brown respectively. S13 and S14 Figs Cluster frequency occurrence in total percentage (left) and per season (right) for the future projections presented in section 3.3. Spring, summer, autumn and winter seasons are in brown, pink, violet and grey respectively. S15 to S25 Figs Surface energy balance per season and per Local Weather Type from the CAPITOUL campaign. S15 corresponds to the seasonal mean. S16 to S25 corresponds to the LWT 0 to the LWT 10 respectively. S26 to S30 Figs Mean Urban Heat Island intensity per season and per Local Weather Type from the CAPITOUL campaign. S26 corresponds to LWT 0 (up) and 1 (bottom); S27 corresponds to LWT 2 (up) and 3 (bottom); S28 corresponds to LWT 4 (up) and 5 (bottom); S29 corresponds to LWT 6 (up) and 7 (bottom); S30 corresponds to LWT 8 (up) and 9 (bottom). S31 to S43 Figs Radio-soundings per Local Weather Type from the CAPITOUL campaign. S31 and S32 corresponds to LWT 0 in winter and autumn; S22 corresponds to LWT 2 in autumn; S34 to S36 corresponds to LWT3 in winter, spring and summer; S37 and S38 corresponds to LWT5 in winter and autumn; S39 corresponds to LWT 7 in summer; S40 corresponds to LWT 8 in summer, S41 to S43 corresponds to LWT 9 in winter, spring and summer respectively. (ZIP) [file pone.0208138.s003.zip › Supporting_Information/S22.tiff]

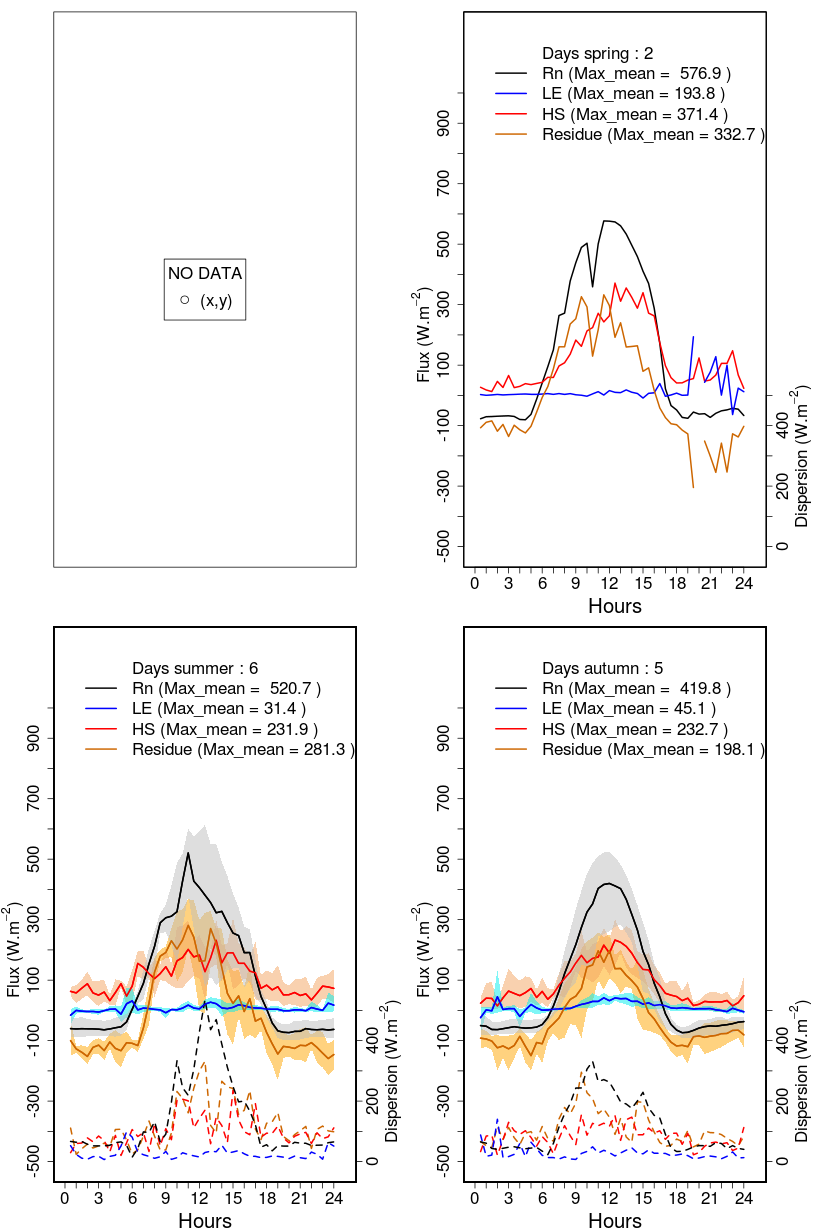

Supplement: S2 File — S2 to S12 Figs LWT from 0 to 10 (a) Mean diurnal cycles of temperature, precipitation, relative humidity, wind force, wind-rose. (b) Mean diurnal cycles of global solar radiation, downward atmospheric radiation and air pressure for Toulouse. Seasonal intra-clusters differences for winter, spring, summer and autumn are presented in grey, light pink, dark pink and brown respectively. S13 and S14 Figs Cluster frequency occurrence in total percentage (left) and per season (right) for the future projections presented in section 3.3. Spring, summer, autumn and winter seasons are in brown, pink, violet and grey respectively. S15 to S25 Figs Surface energy balance per season and per Local Weather Type from the CAPITOUL campaign. S15 corresponds to the seasonal mean. S16 to S25 corresponds to the LWT 0 to the LWT 10 respectively. S26 to S30 Figs Mean Urban Heat Island intensity per season and per Local Weather Type from the CAPITOUL campaign. S26 corresponds to LWT 0 (up) and 1 (bottom); S27 corresponds to LWT 2 (up) and 3 (bottom); S28 corresponds to LWT 4 (up) and 5 (bottom); S29 corresponds to LWT 6 (up) and 7 (bottom); S30 corresponds to LWT 8 (up) and 9 (bottom). S31 to S43 Figs Radio-soundings per Local Weather Type from the CAPITOUL campaign. S31 and S32 corresponds to LWT 0 in winter and autumn; S22 corresponds to LWT 2 in autumn; S34 to S36 corresponds to LWT3 in winter, spring and summer; S37 and S38 corresponds to LWT5 in winter and autumn; S39 corresponds to LWT 7 in summer; S40 corresponds to LWT 8 in summer, S41 to S43 corresponds to LWT 9 in winter, spring and summer respectively. (ZIP) [file pone.0208138.s003.zip › Supporting_Information/S23.tiff]

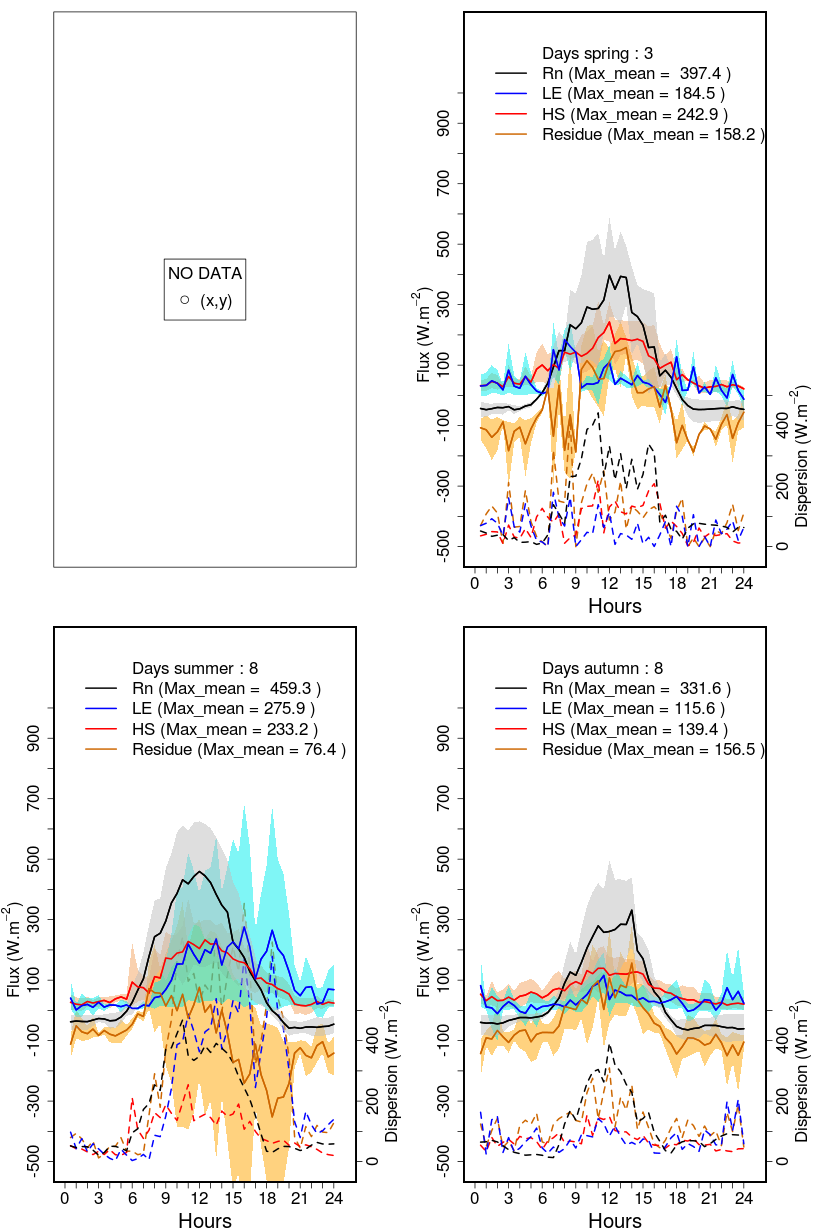

Supplement: S2 File — S2 to S12 Figs LWT from 0 to 10 (a) Mean diurnal cycles of temperature, precipitation, relative humidity, wind force, wind-rose. (b) Mean diurnal cycles of global solar radiation, downward atmospheric radiation and air pressure for Toulouse. Seasonal intra-clusters differences for winter, spring, summer and autumn are presented in grey, light pink, dark pink and brown respectively. S13 and S14 Figs Cluster frequency occurrence in total percentage (left) and per season (right) for the future projections presented in section 3.3. Spring, summer, autumn and winter seasons are in brown, pink, violet and grey respectively. S15 to S25 Figs Surface energy balance per season and per Local Weather Type from the CAPITOUL campaign. S15 corresponds to the seasonal mean. S16 to S25 corresponds to the LWT 0 to the LWT 10 respectively. S26 to S30 Figs Mean Urban Heat Island intensity per season and per Local Weather Type from the CAPITOUL campaign. S26 corresponds to LWT 0 (up) and 1 (bottom); S27 corresponds to LWT 2 (up) and 3 (bottom); S28 corresponds to LWT 4 (up) and 5 (bottom); S29 corresponds to LWT 6 (up) and 7 (bottom); S30 corresponds to LWT 8 (up) and 9 (bottom). S31 to S43 Figs Radio-soundings per Local Weather Type from the CAPITOUL campaign. S31 and S32 corresponds to LWT 0 in winter and autumn; S22 corresponds to LWT 2 in autumn; S34 to S36 corresponds to LWT3 in winter, spring and summer; S37 and S38 corresponds to LWT5 in winter and autumn; S39 corresponds to LWT 7 in summer; S40 corresponds to LWT 8 in summer, S41 to S43 corresponds to LWT 9 in winter, spring and summer respectively. (ZIP) [file pone.0208138.s003.zip › Supporting_Information/S24.tiff]

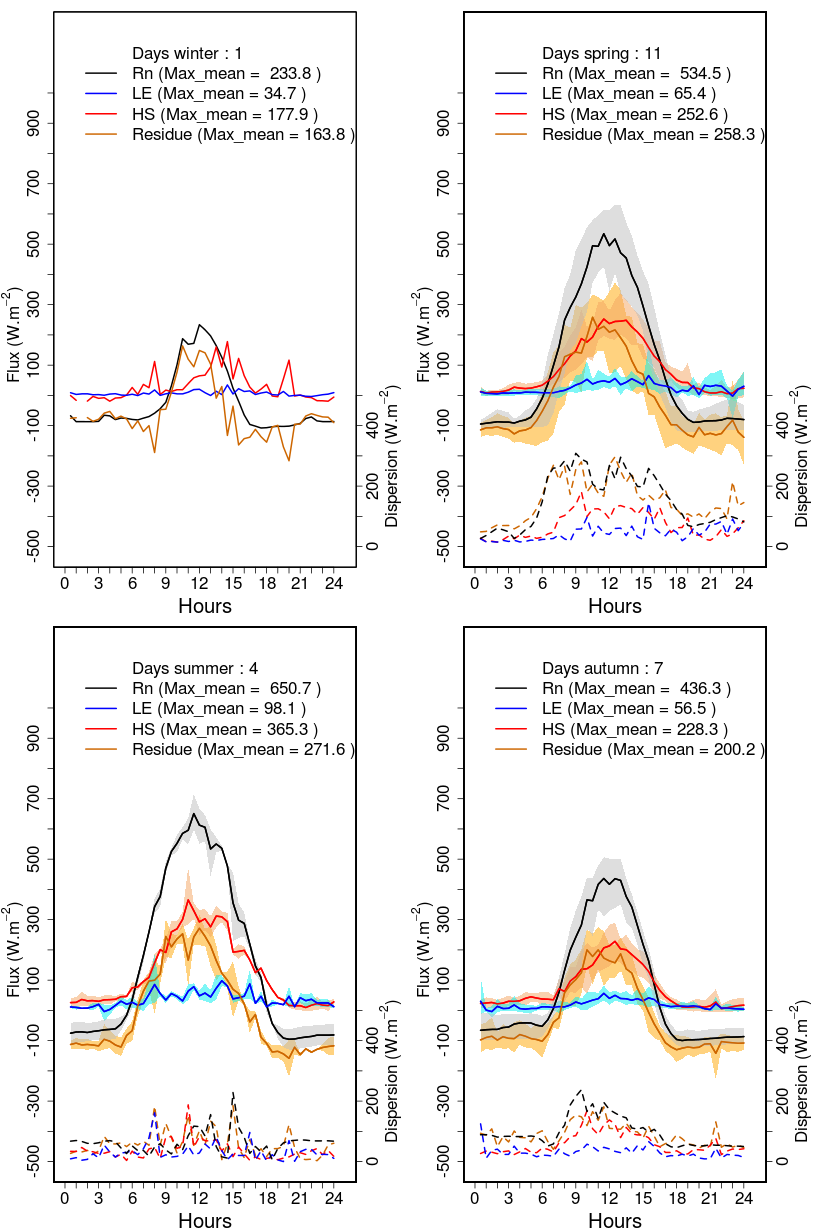

Supplement: S2 File — S2 to S12 Figs LWT from 0 to 10 (a) Mean diurnal cycles of temperature, precipitation, relative humidity, wind force, wind-rose. (b) Mean diurnal cycles of global solar radiation, downward atmospheric radiation and air pressure for Toulouse. Seasonal intra-clusters differences for winter, spring, summer and autumn are presented in grey, light pink, dark pink and brown respectively. S13 and S14 Figs Cluster frequency occurrence in total percentage (left) and per season (right) for the future projections presented in section 3.3. Spring, summer, autumn and winter seasons are in brown, pink, violet and grey respectively. S15 to S25 Figs Surface energy balance per season and per Local Weather Type from the CAPITOUL campaign. S15 corresponds to the seasonal mean. S16 to S25 corresponds to the LWT 0 to the LWT 10 respectively. S26 to S30 Figs Mean Urban Heat Island intensity per season and per Local Weather Type from the CAPITOUL campaign. S26 corresponds to LWT 0 (up) and 1 (bottom); S27 corresponds to LWT 2 (up) and 3 (bottom); S28 corresponds to LWT 4 (up) and 5 (bottom); S29 corresponds to LWT 6 (up) and 7 (bottom); S30 corresponds to LWT 8 (up) and 9 (bottom). S31 to S43 Figs Radio-soundings per Local Weather Type from the CAPITOUL campaign. S31 and S32 corresponds to LWT 0 in winter and autumn; S22 corresponds to LWT 2 in autumn; S34 to S36 corresponds to LWT3 in winter, spring and summer; S37 and S38 corresponds to LWT5 in winter and autumn; S39 corresponds to LWT 7 in summer; S40 corresponds to LWT 8 in summer, S41 to S43 corresponds to LWT 9 in winter, spring and summer respectively. (ZIP) [file pone.0208138.s003.zip › Supporting_Information/S25.tiff]

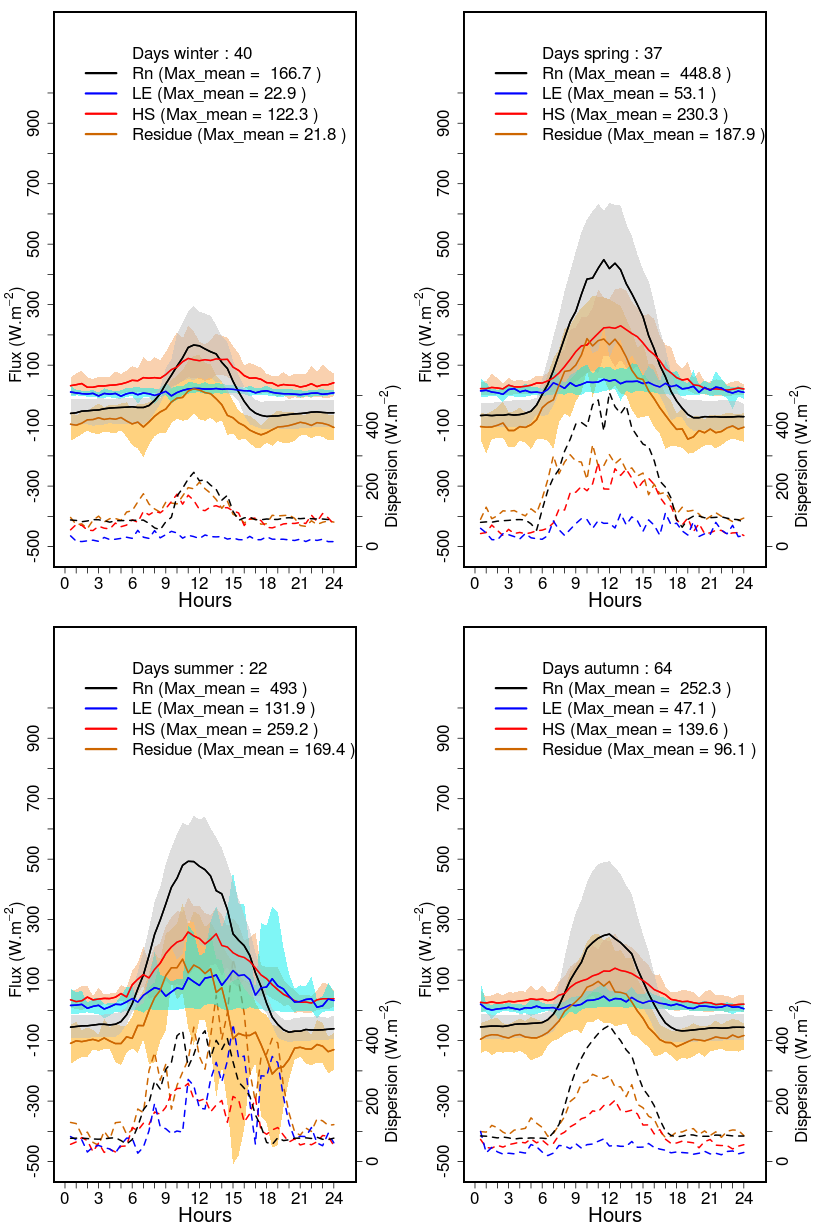

Supplement: S2 File — S2 to S12 Figs LWT from 0 to 10 (a) Mean diurnal cycles of temperature, precipitation, relative humidity, wind force, wind-rose. (b) Mean diurnal cycles of global solar radiation, downward atmospheric radiation and air pressure for Toulouse. Seasonal intra-clusters differences for winter, spring, summer and autumn are presented in grey, light pink, dark pink and brown respectively. S13 and S14 Figs Cluster frequency occurrence in total percentage (left) and per season (right) for the future projections presented in section 3.3. Spring, summer, autumn and winter seasons are in brown, pink, violet and grey respectively. S15 to S25 Figs Surface energy balance per season and per Local Weather Type from the CAPITOUL campaign. S15 corresponds to the seasonal mean. S16 to S25 corresponds to the LWT 0 to the LWT 10 respectively. S26 to S30 Figs Mean Urban Heat Island intensity per season and per Local Weather Type from the CAPITOUL campaign. S26 corresponds to LWT 0 (up) and 1 (bottom); S27 corresponds to LWT 2 (up) and 3 (bottom); S28 corresponds to LWT 4 (up) and 5 (bottom); S29 corresponds to LWT 6 (up) and 7 (bottom); S30 corresponds to LWT 8 (up) and 9 (bottom). S31 to S43 Figs Radio-soundings per Local Weather Type from the CAPITOUL campaign. S31 and S32 corresponds to LWT 0 in winter and autumn; S22 corresponds to LWT 2 in autumn; S34 to S36 corresponds to LWT3 in winter, spring and summer; S37 and S38 corresponds to LWT5 in winter and autumn; S39 corresponds to LWT 7 in summer; S40 corresponds to LWT 8 in summer, S41 to S43 corresponds to LWT 9 in winter, spring and summer respectively. (ZIP) [file pone.0208138.s003.zip › Supporting_Information/S15.tiff]

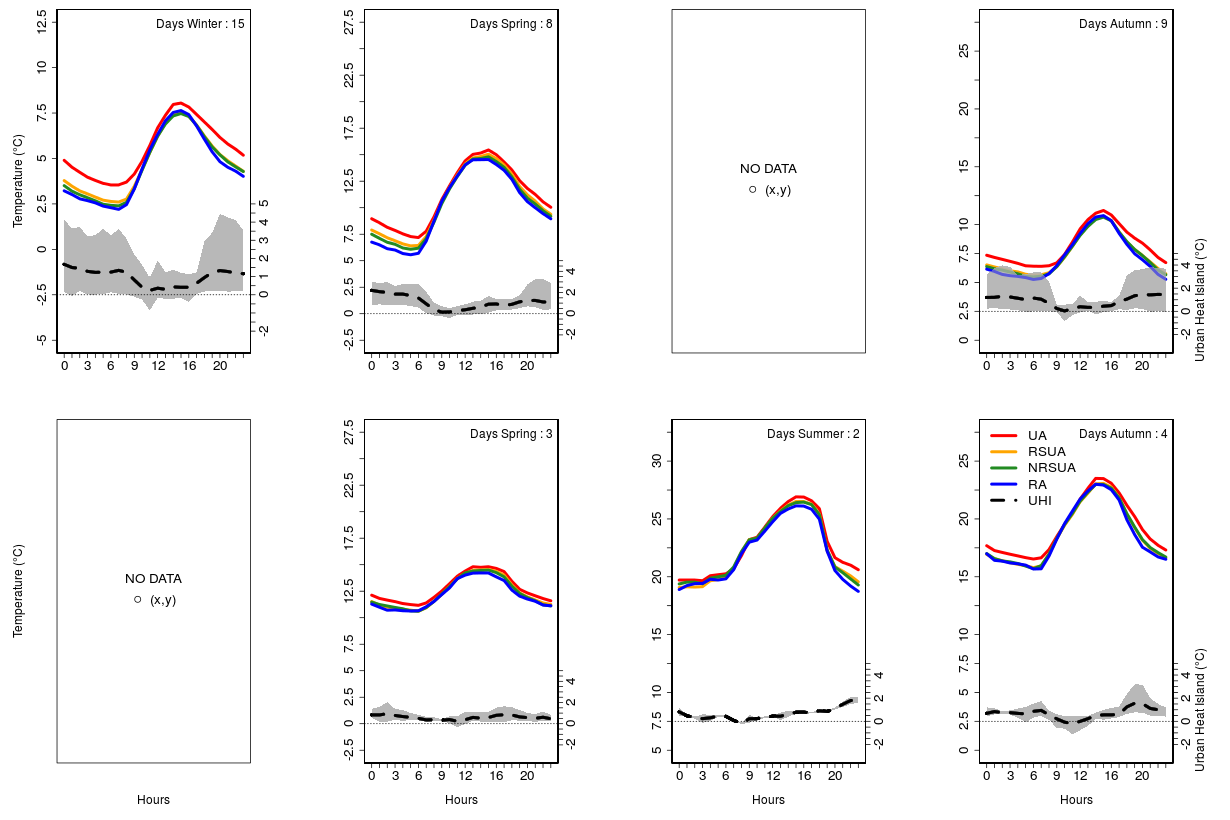

Supplement: S2 File — S2 to S12 Figs LWT from 0 to 10 (a) Mean diurnal cycles of temperature, precipitation, relative humidity, wind force, wind-rose. (b) Mean diurnal cycles of global solar radiation, downward atmospheric radiation and air pressure for Toulouse. Seasonal intra-clusters differences for winter, spring, summer and autumn are presented in grey, light pink, dark pink and brown respectively. S13 and S14 Figs Cluster frequency occurrence in total percentage (left) and per season (right) for the future projections presented in section 3.3. Spring, summer, autumn and winter seasons are in brown, pink, violet and grey respectively. S15 to S25 Figs Surface energy balance per season and per Local Weather Type from the CAPITOUL campaign. S15 corresponds to the seasonal mean. S16 to S25 corresponds to the LWT 0 to the LWT 10 respectively. S26 to S30 Figs Mean Urban Heat Island intensity per season and per Local Weather Type from the CAPITOUL campaign. S26 corresponds to LWT 0 (up) and 1 (bottom); S27 corresponds to LWT 2 (up) and 3 (bottom); S28 corresponds to LWT 4 (up) and 5 (bottom); S29 corresponds to LWT 6 (up) and 7 (bottom); S30 corresponds to LWT 8 (up) and 9 (bottom). S31 to S43 Figs Radio-soundings per Local Weather Type from the CAPITOUL campaign. S31 and S32 corresponds to LWT 0 in winter and autumn; S22 corresponds to LWT 2 in autumn; S34 to S36 corresponds to LWT3 in winter, spring and summer; S37 and S38 corresponds to LWT5 in winter and autumn; S39 corresponds to LWT 7 in summer; S40 corresponds to LWT 8 in summer, S41 to S43 corresponds to LWT 9 in winter, spring and summer respectively. (ZIP) [file pone.0208138.s003.zip › Supporting_Information/S26.tiff]

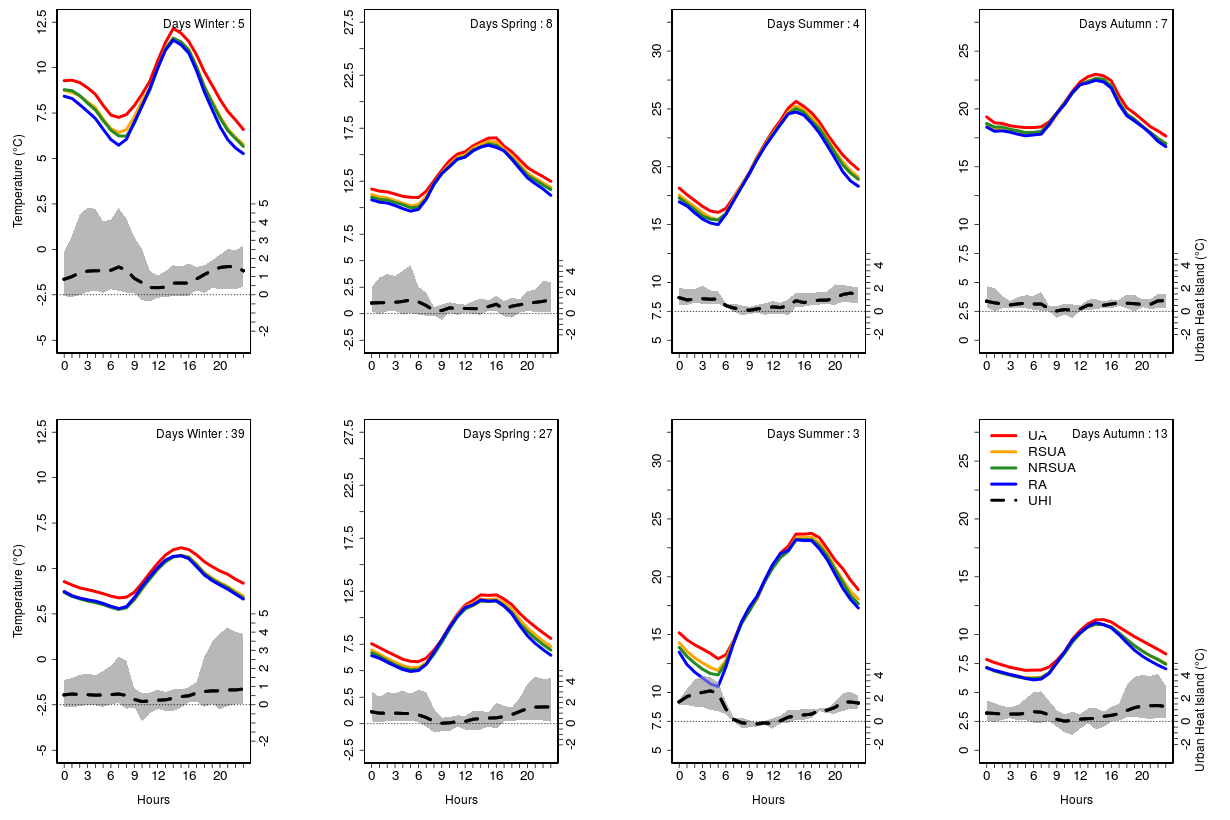

Supplement: S2 File — S2 to S12 Figs LWT from 0 to 10 (a) Mean diurnal cycles of temperature, precipitation, relative humidity, wind force, wind-rose. (b) Mean diurnal cycles of global solar radiation, downward atmospheric radiation and air pressure for Toulouse. Seasonal intra-clusters differences for winter, spring, summer and autumn are presented in grey, light pink, dark pink and brown respectively. S13 and S14 Figs Cluster frequency occurrence in total percentage (left) and per season (right) for the future projections presented in section 3.3. Spring, summer, autumn and winter seasons are in brown, pink, violet and grey respectively. S15 to S25 Figs Surface energy balance per season and per Local Weather Type from the CAPITOUL campaign. S15 corresponds to the seasonal mean. S16 to S25 corresponds to the LWT 0 to the LWT 10 respectively. S26 to S30 Figs Mean Urban Heat Island intensity per season and per Local Weather Type from the CAPITOUL campaign. S26 corresponds to LWT 0 (up) and 1 (bottom); S27 corresponds to LWT 2 (up) and 3 (bottom); S28 corresponds to LWT 4 (up) and 5 (bottom); S29 corresponds to LWT 6 (up) and 7 (bottom); S30 corresponds to LWT 8 (up) and 9 (bottom). S31 to S43 Figs Radio-soundings per Local Weather Type from the CAPITOUL campaign. S31 and S32 corresponds to LWT 0 in winter and autumn; S22 corresponds to LWT 2 in autumn; S34 to S36 corresponds to LWT3 in winter, spring and summer; S37 and S38 corresponds to LWT5 in winter and autumn; S39 corresponds to LWT 7 in summer; S40 corresponds to LWT 8 in summer, S41 to S43 corresponds to LWT 9 in winter, spring and summer respectively. (ZIP) [file pone.0208138.s003.zip › Supporting_Information/S27.tiff]

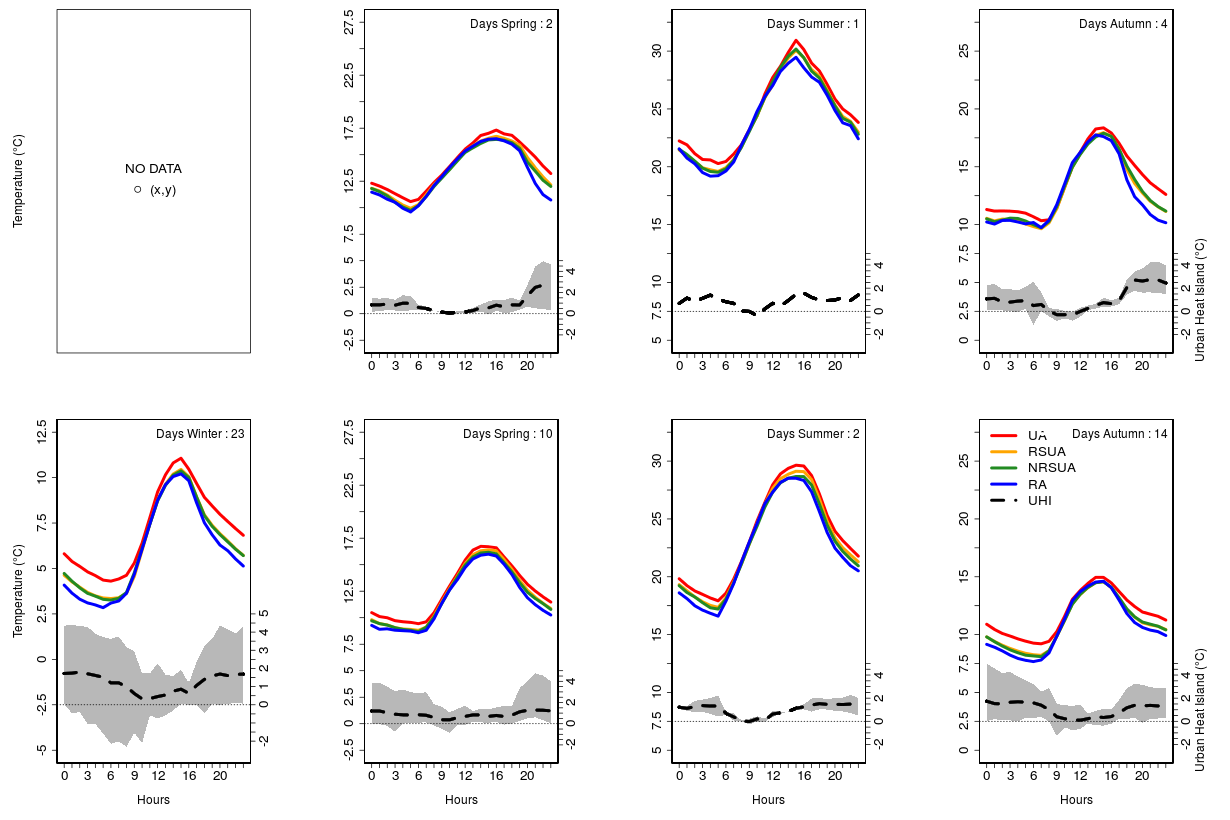

Supplement: S2 File — S2 to S12 Figs LWT from 0 to 10 (a) Mean diurnal cycles of temperature, precipitation, relative humidity, wind force, wind-rose. (b) Mean diurnal cycles of global solar radiation, downward atmospheric radiation and air pressure for Toulouse. Seasonal intra-clusters differences for winter, spring, summer and autumn are presented in grey, light pink, dark pink and brown respectively. S13 and S14 Figs Cluster frequency occurrence in total percentage (left) and per season (right) for the future projections presented in section 3.3. Spring, summer, autumn and winter seasons are in brown, pink, violet and grey respectively. S15 to S25 Figs Surface energy balance per season and per Local Weather Type from the CAPITOUL campaign. S15 corresponds to the seasonal mean. S16 to S25 corresponds to the LWT 0 to the LWT 10 respectively. S26 to S30 Figs Mean Urban Heat Island intensity per season and per Local Weather Type from the CAPITOUL campaign. S26 corresponds to LWT 0 (up) and 1 (bottom); S27 corresponds to LWT 2 (up) and 3 (bottom); S28 corresponds to LWT 4 (up) and 5 (bottom); S29 corresponds to LWT 6 (up) and 7 (bottom); S30 corresponds to LWT 8 (up) and 9 (bottom). S31 to S43 Figs Radio-soundings per Local Weather Type from the CAPITOUL campaign. S31 and S32 corresponds to LWT 0 in winter and autumn; S22 corresponds to LWT 2 in autumn; S34 to S36 corresponds to LWT3 in winter, spring and summer; S37 and S38 corresponds to LWT5 in winter and autumn; S39 corresponds to LWT 7 in summer; S40 corresponds to LWT 8 in summer, S41 to S43 corresponds to LWT 9 in winter, spring and summer respectively. (ZIP) [file pone.0208138.s003.zip › Supporting_Information/S28.tiff]

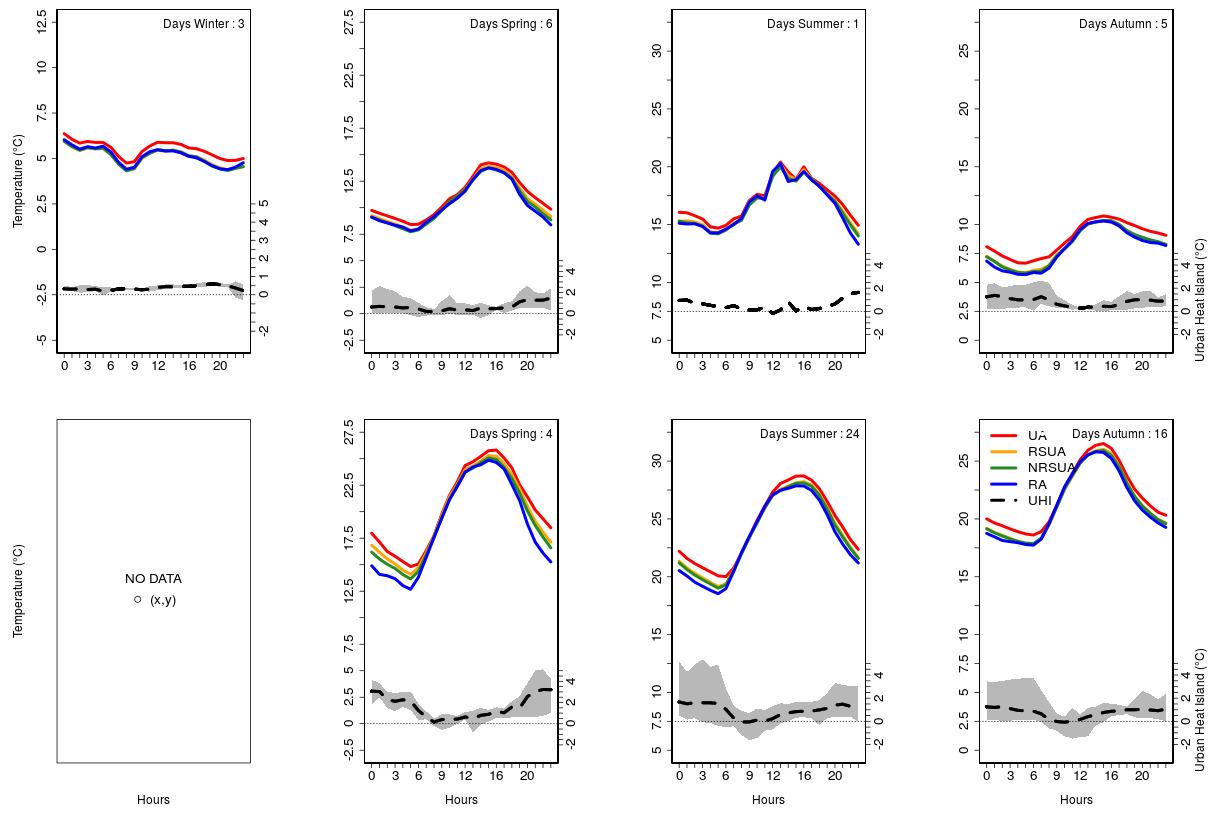

Supplement: S2 File — S2 to S12 Figs LWT from 0 to 10 (a) Mean diurnal cycles of temperature, precipitation, relative humidity, wind force, wind-rose. (b) Mean diurnal cycles of global solar radiation, downward atmospheric radiation and air pressure for Toulouse. Seasonal intra-clusters differences for winter, spring, summer and autumn are presented in grey, light pink, dark pink and brown respectively. S13 and S14 Figs Cluster frequency occurrence in total percentage (left) and per season (right) for the future projections presented in section 3.3. Spring, summer, autumn and winter seasons are in brown, pink, violet and grey respectively. S15 to S25 Figs Surface energy balance per season and per Local Weather Type from the CAPITOUL campaign. S15 corresponds to the seasonal mean. S16 to S25 corresponds to the LWT 0 to the LWT 10 respectively. S26 to S30 Figs Mean Urban Heat Island intensity per season and per Local Weather Type from the CAPITOUL campaign. S26 corresponds to LWT 0 (up) and 1 (bottom); S27 corresponds to LWT 2 (up) and 3 (bottom); S28 corresponds to LWT 4 (up) and 5 (bottom); S29 corresponds to LWT 6 (up) and 7 (bottom); S30 corresponds to LWT 8 (up) and 9 (bottom). S31 to S43 Figs Radio-soundings per Local Weather Type from the CAPITOUL campaign. S31 and S32 corresponds to LWT 0 in winter and autumn; S22 corresponds to LWT 2 in autumn; S34 to S36 corresponds to LWT3 in winter, spring and summer; S37 and S38 corresponds to LWT5 in winter and autumn; S39 corresponds to LWT 7 in summer; S40 corresponds to LWT 8 in summer, S41 to S43 corresponds to LWT 9 in winter, spring and summer respectively. (ZIP) [file pone.0208138.s003.zip › Supporting_Information/S29.tiff]

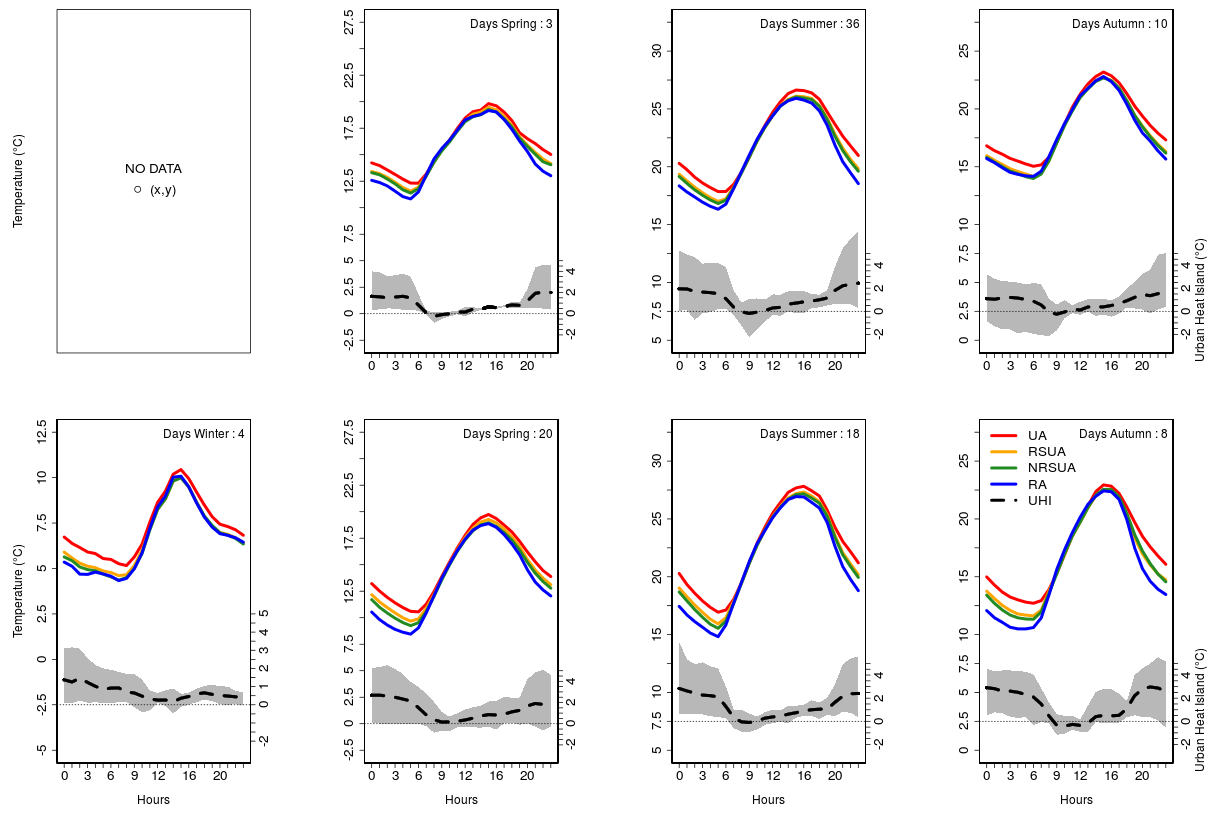

Supplement: S2 File — S2 to S12 Figs LWT from 0 to 10 (a) Mean diurnal cycles of temperature, precipitation, relative humidity, wind force, wind-rose. (b) Mean diurnal cycles of global solar radiation, downward atmospheric radiation and air pressure for Toulouse. Seasonal intra-clusters differences for winter, spring, summer and autumn are presented in grey, light pink, dark pink and brown respectively. S13 and S14 Figs Cluster frequency occurrence in total percentage (left) and per season (right) for the future projections presented in section 3.3. Spring, summer, autumn and winter seasons are in brown, pink, violet and grey respectively. S15 to S25 Figs Surface energy balance per season and per Local Weather Type from the CAPITOUL campaign. S15 corresponds to the seasonal mean. S16 to S25 corresponds to the LWT 0 to the LWT 10 respectively. S26 to S30 Figs Mean Urban Heat Island intensity per season and per Local Weather Type from the CAPITOUL campaign. S26 corresponds to LWT 0 (up) and 1 (bottom); S27 corresponds to LWT 2 (up) and 3 (bottom); S28 corresponds to LWT 4 (up) and 5 (bottom); S29 corresponds to LWT 6 (up) and 7 (bottom); S30 corresponds to LWT 8 (up) and 9 (bottom). S31 to S43 Figs Radio-soundings per Local Weather Type from the CAPITOUL campaign. S31 and S32 corresponds to LWT 0 in winter and autumn; S22 corresponds to LWT 2 in autumn; S34 to S36 corresponds to LWT3 in winter, spring and summer; S37 and S38 corresponds to LWT5 in winter and autumn; S39 corresponds to LWT 7 in summer; S40 corresponds to LWT 8 in summer, S41 to S43 corresponds to LWT 9 in winter, spring and summer respectively. (ZIP) [file pone.0208138.s003.zip › Supporting_Information/S30.tiff]

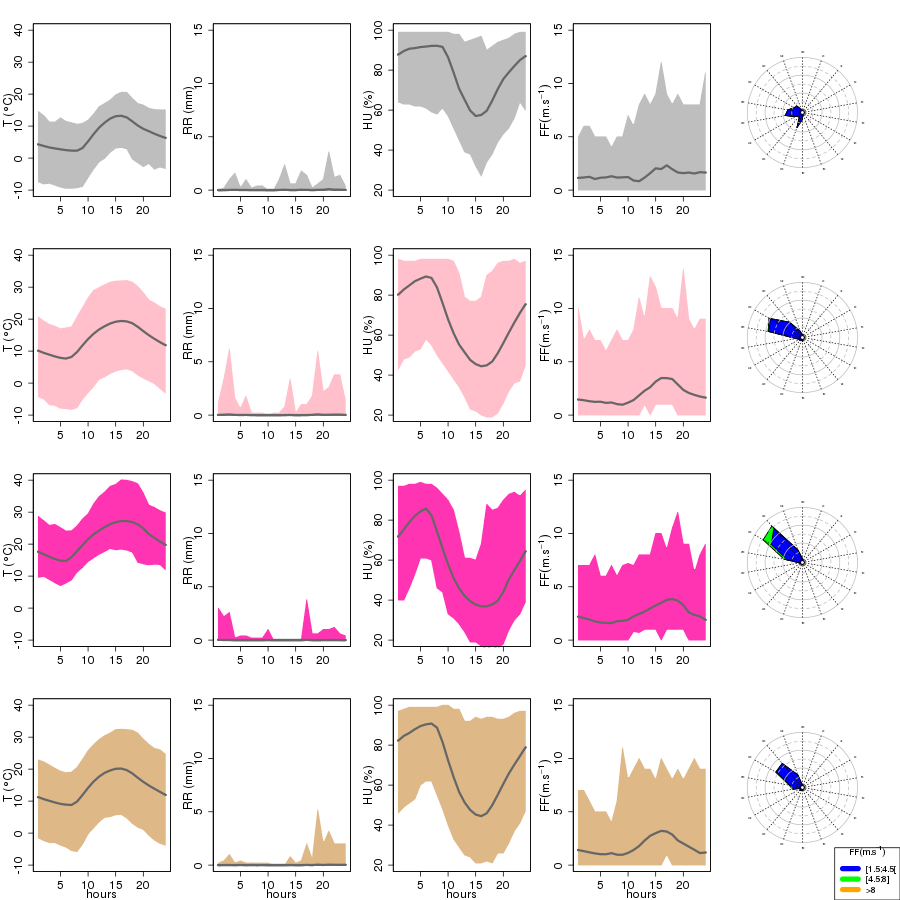

Supplement: S2 File — S2 to S12 Figs LWT from 0 to 10 (a) Mean diurnal cycles of temperature, precipitation, relative humidity, wind force, wind-rose. (b) Mean diurnal cycles of global solar radiation, downward atmospheric radiation and air pressure for Toulouse. Seasonal intra-clusters differences for winter, spring, summer and autumn are presented in grey, light pink, dark pink and brown respectively. S13 and S14 Figs Cluster frequency occurrence in total percentage (left) and per season (right) for the future projections presented in section 3.3. Spring, summer, autumn and winter seasons are in brown, pink, violet and grey respectively. S15 to S25 Figs Surface energy balance per season and per Local Weather Type from the CAPITOUL campaign. S15 corresponds to the seasonal mean. S16 to S25 corresponds to the LWT 0 to the LWT 10 respectively. S26 to S30 Figs Mean Urban Heat Island intensity per season and per Local Weather Type from the CAPITOUL campaign. S26 corresponds to LWT 0 (up) and 1 (bottom); S27 corresponds to LWT 2 (up) and 3 (bottom); S28 corresponds to LWT 4 (up) and 5 (bottom); S29 corresponds to LWT 6 (up) and 7 (bottom); S30 corresponds to LWT 8 (up) and 9 (bottom). S31 to S43 Figs Radio-soundings per Local Weather Type from the CAPITOUL campaign. S31 and S32 corresponds to LWT 0 in winter and autumn; S22 corresponds to LWT 2 in autumn; S34 to S36 corresponds to LWT3 in winter, spring and summer; S37 and S38 corresponds to LWT5 in winter and autumn; S39 corresponds to LWT 7 in summer; S40 corresponds to LWT 8 in summer, S41 to S43 corresponds to LWT 9 in winter, spring and summer respectively. (ZIP) [file pone.0208138.s003.zip › Supporting_Information/S11_Fig_a.tiff]

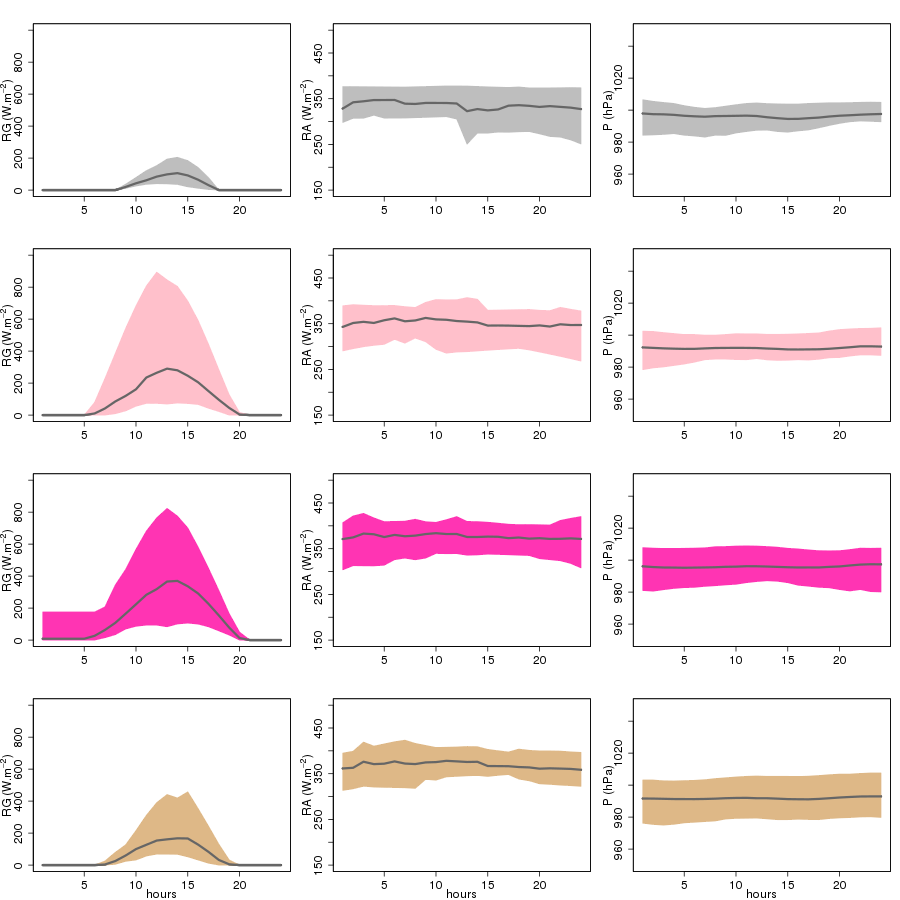

Supplement: S2 File — S2 to S12 Figs LWT from 0 to 10 (a) Mean diurnal cycles of temperature, precipitation, relative humidity, wind force, wind-rose. (b) Mean diurnal cycles of global solar radiation, downward atmospheric radiation and air pressure for Toulouse. Seasonal intra-clusters differences for winter, spring, summer and autumn are presented in grey, light pink, dark pink and brown respectively. S13 and S14 Figs Cluster frequency occurrence in total percentage (left) and per season (right) for the future projections presented in section 3.3. Spring, summer, autumn and winter seasons are in brown, pink, violet and grey respectively. S15 to S25 Figs Surface energy balance per season and per Local Weather Type from the CAPITOUL campaign. S15 corresponds to the seasonal mean. S16 to S25 corresponds to the LWT 0 to the LWT 10 respectively. S26 to S30 Figs Mean Urban Heat Island intensity per season and per Local Weather Type from the CAPITOUL campaign. S26 corresponds to LWT 0 (up) and 1 (bottom); S27 corresponds to LWT 2 (up) and 3 (bottom); S28 corresponds to LWT 4 (up) and 5 (bottom); S29 corresponds to LWT 6 (up) and 7 (bottom); S30 corresponds to LWT 8 (up) and 9 (bottom). S31 to S43 Figs Radio-soundings per Local Weather Type from the CAPITOUL campaign. S31 and S32 corresponds to LWT 0 in winter and autumn; S22 corresponds to LWT 2 in autumn; S34 to S36 corresponds to LWT3 in winter, spring and summer; S37 and S38 corresponds to LWT5 in winter and autumn; S39 corresponds to LWT 7 in summer; S40 corresponds to LWT 8 in summer, S41 to S43 corresponds to LWT 9 in winter, spring and summer respectively. (ZIP) [file pone.0208138.s003.zip › Supporting_Information/S6_Fig_b.tiff]

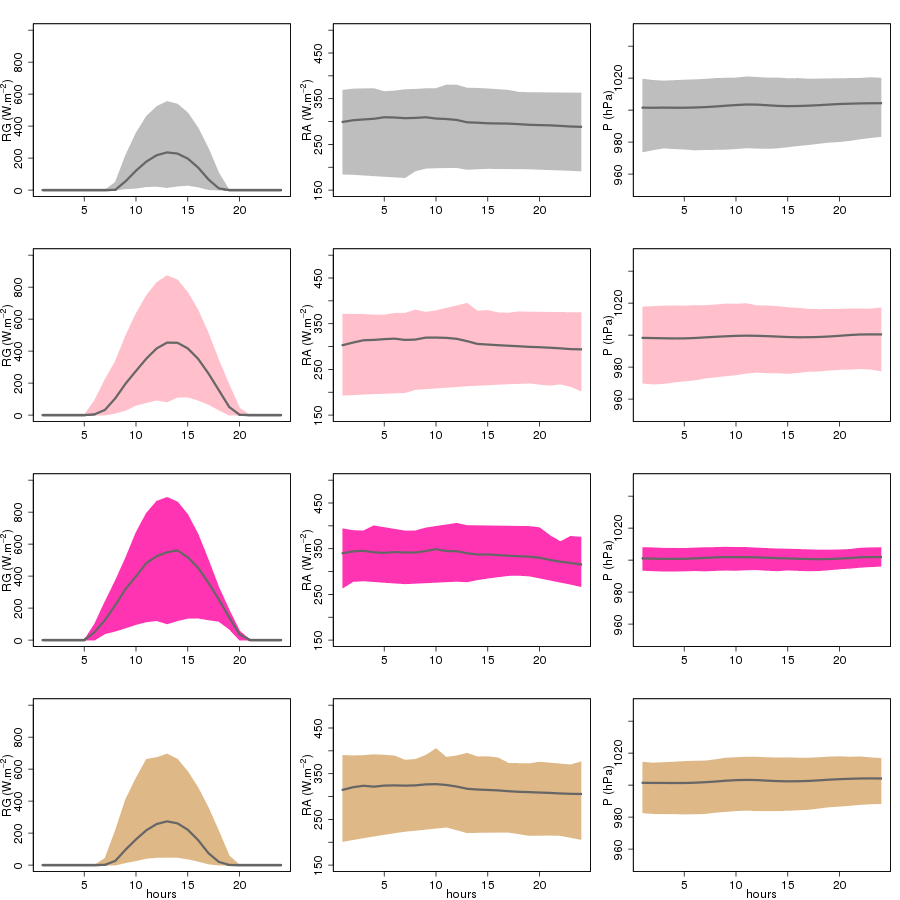

Supplement: S2 File — S2 to S12 Figs LWT from 0 to 10 (a) Mean diurnal cycles of temperature, precipitation, relative humidity, wind force, wind-rose. (b) Mean diurnal cycles of global solar radiation, downward atmospheric radiation and air pressure for Toulouse. Seasonal intra-clusters differences for winter, spring, summer and autumn are presented in grey, light pink, dark pink and brown respectively. S13 and S14 Figs Cluster frequency occurrence in total percentage (left) and per season (right) for the future projections presented in section 3.3. Spring, summer, autumn and winter seasons are in brown, pink, violet and grey respectively. S15 to S25 Figs Surface energy balance per season and per Local Weather Type from the CAPITOUL campaign. S15 corresponds to the seasonal mean. S16 to S25 corresponds to the LWT 0 to the LWT 10 respectively. S26 to S30 Figs Mean Urban Heat Island intensity per season and per Local Weather Type from the CAPITOUL campaign. S26 corresponds to LWT 0 (up) and 1 (bottom); S27 corresponds to LWT 2 (up) and 3 (bottom); S28 corresponds to LWT 4 (up) and 5 (bottom); S29 corresponds to LWT 6 (up) and 7 (bottom); S30 corresponds to LWT 8 (up) and 9 (bottom). S31 to S43 Figs Radio-soundings per Local Weather Type from the CAPITOUL campaign. S31 and S32 corresponds to LWT 0 in winter and autumn; S22 corresponds to LWT 2 in autumn; S34 to S36 corresponds to LWT3 in winter, spring and summer; S37 and S38 corresponds to LWT5 in winter and autumn; S39 corresponds to LWT 7 in summer; S40 corresponds to LWT 8 in summer, S41 to S43 corresponds to LWT 9 in winter, spring and summer respectively. (ZIP) [file pone.0208138.s003.zip › Supporting_Information/S5_Fig_b.tiff]

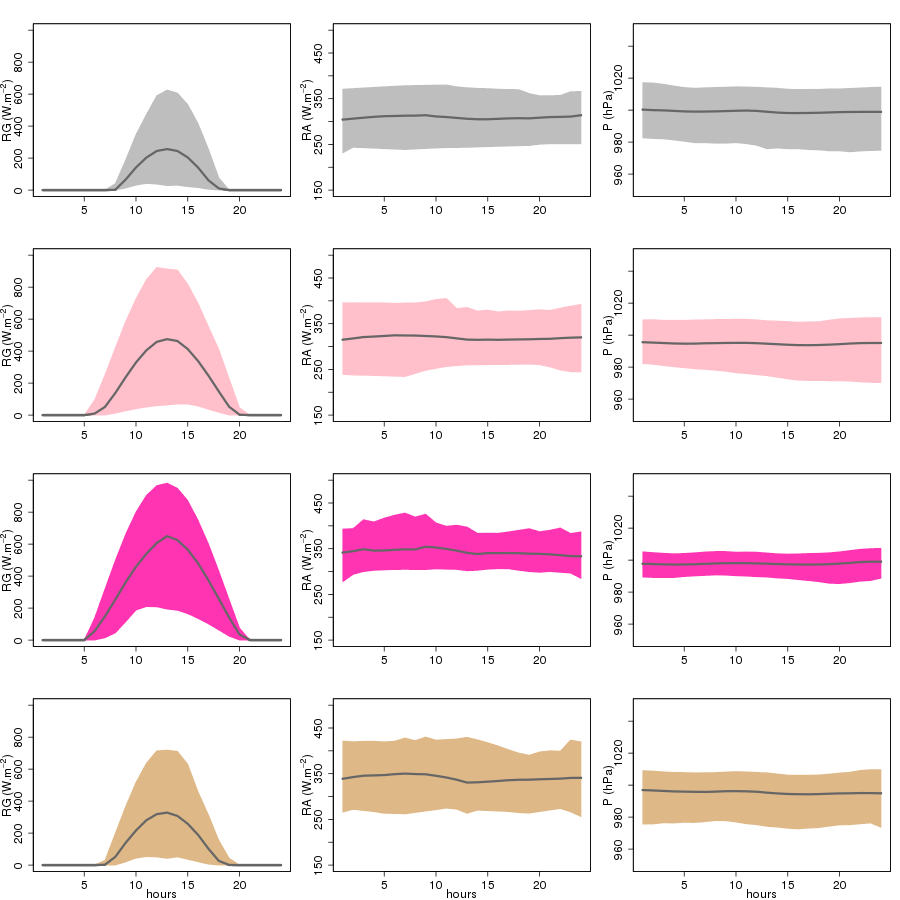

Supplement: S2 File — S2 to S12 Figs LWT from 0 to 10 (a) Mean diurnal cycles of temperature, precipitation, relative humidity, wind force, wind-rose. (b) Mean diurnal cycles of global solar radiation, downward atmospheric radiation and air pressure for Toulouse. Seasonal intra-clusters differences for winter, spring, summer and autumn are presented in grey, light pink, dark pink and brown respectively. S13 and S14 Figs Cluster frequency occurrence in total percentage (left) and per season (right) for the future projections presented in section 3.3. Spring, summer, autumn and winter seasons are in brown, pink, violet and grey respectively. S15 to S25 Figs Surface energy balance per season and per Local Weather Type from the CAPITOUL campaign. S15 corresponds to the seasonal mean. S16 to S25 corresponds to the LWT 0 to the LWT 10 respectively. S26 to S30 Figs Mean Urban Heat Island intensity per season and per Local Weather Type from the CAPITOUL campaign. S26 corresponds to LWT 0 (up) and 1 (bottom); S27 corresponds to LWT 2 (up) and 3 (bottom); S28 corresponds to LWT 4 (up) and 5 (bottom); S29 corresponds to LWT 6 (up) and 7 (bottom); S30 corresponds to LWT 8 (up) and 9 (bottom). S31 to S43 Figs Radio-soundings per Local Weather Type from the CAPITOUL campaign. S31 and S32 corresponds to LWT 0 in winter and autumn; S22 corresponds to LWT 2 in autumn; S34 to S36 corresponds to LWT3 in winter, spring and summer; S37 and S38 corresponds to LWT5 in winter and autumn; S39 corresponds to LWT 7 in summer; S40 corresponds to LWT 8 in summer, S41 to S43 corresponds to LWT 9 in winter, spring and summer respectively. (ZIP) [file pone.0208138.s003.zip › Supporting_Information/S4_Fig_b.tiff]

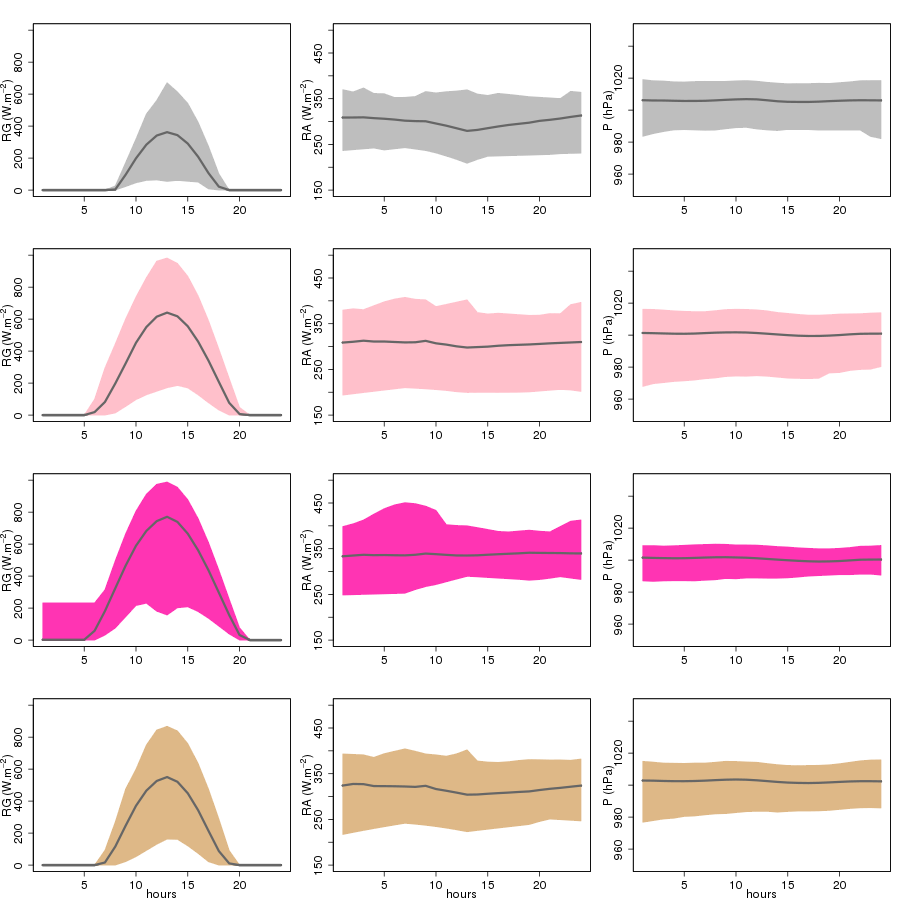

Supplement: S2 File — S2 to S12 Figs LWT from 0 to 10 (a) Mean diurnal cycles of temperature, precipitation, relative humidity, wind force, wind-rose. (b) Mean diurnal cycles of global solar radiation, downward atmospheric radiation and air pressure for Toulouse. Seasonal intra-clusters differences for winter, spring, summer and autumn are presented in grey, light pink, dark pink and brown respectively. S13 and S14 Figs Cluster frequency occurrence in total percentage (left) and per season (right) for the future projections presented in section 3.3. Spring, summer, autumn and winter seasons are in brown, pink, violet and grey respectively. S15 to S25 Figs Surface energy balance per season and per Local Weather Type from the CAPITOUL campaign. S15 corresponds to the seasonal mean. S16 to S25 corresponds to the LWT 0 to the LWT 10 respectively. S26 to S30 Figs Mean Urban Heat Island intensity per season and per Local Weather Type from the CAPITOUL campaign. S26 corresponds to LWT 0 (up) and 1 (bottom); S27 corresponds to LWT 2 (up) and 3 (bottom); S28 corresponds to LWT 4 (up) and 5 (bottom); S29 corresponds to LWT 6 (up) and 7 (bottom); S30 corresponds to LWT 8 (up) and 9 (bottom). S31 to S43 Figs Radio-soundings per Local Weather Type from the CAPITOUL campaign. S31 and S32 corresponds to LWT 0 in winter and autumn; S22 corresponds to LWT 2 in autumn; S34 to S36 corresponds to LWT3 in winter, spring and summer; S37 and S38 corresponds to LWT5 in winter and autumn; S39 corresponds to LWT 7 in summer; S40 corresponds to LWT 8 in summer, S41 to S43 corresponds to LWT 9 in winter, spring and summer respectively. (ZIP) [file pone.0208138.s003.zip › Supporting_Information/S11_Fig_b.tiff]

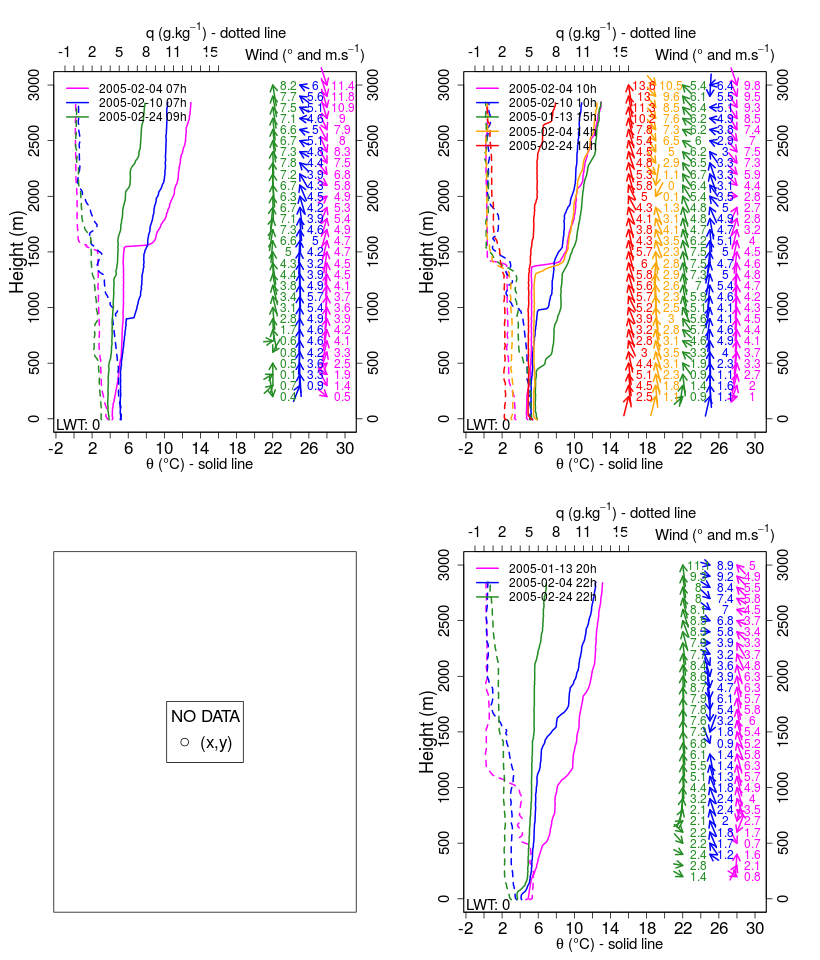

Supplement: S2 File — S2 to S12 Figs LWT from 0 to 10 (a) Mean diurnal cycles of temperature, precipitation, relative humidity, wind force, wind-rose. (b) Mean diurnal cycles of global solar radiation, downward atmospheric radiation and air pressure for Toulouse. Seasonal intra-clusters differences for winter, spring, summer and autumn are presented in grey, light pink, dark pink and brown respectively. S13 and S14 Figs Cluster frequency occurrence in total percentage (left) and per season (right) for the future projections presented in section 3.3. Spring, summer, autumn and winter seasons are in brown, pink, violet and grey respectively. S15 to S25 Figs Surface energy balance per season and per Local Weather Type from the CAPITOUL campaign. S15 corresponds to the seasonal mean. S16 to S25 corresponds to the LWT 0 to the LWT 10 respectively. S26 to S30 Figs Mean Urban Heat Island intensity per season and per Local Weather Type from the CAPITOUL campaign. S26 corresponds to LWT 0 (up) and 1 (bottom); S27 corresponds to LWT 2 (up) and 3 (bottom); S28 corresponds to LWT 4 (up) and 5 (bottom); S29 corresponds to LWT 6 (up) and 7 (bottom); S30 corresponds to LWT 8 (up) and 9 (bottom). S31 to S43 Figs Radio-soundings per Local Weather Type from the CAPITOUL campaign. S31 and S32 corresponds to LWT 0 in winter and autumn; S22 corresponds to LWT 2 in autumn; S34 to S36 corresponds to LWT3 in winter, spring and summer; S37 and S38 corresponds to LWT5 in winter and autumn; S39 corresponds to LWT 7 in summer; S40 corresponds to LWT 8 in summer, S41 to S43 corresponds to LWT 9 in winter, spring and summer respectively. (ZIP) [file pone.0208138.s003.zip › Supporting_Information/S31.tiff]

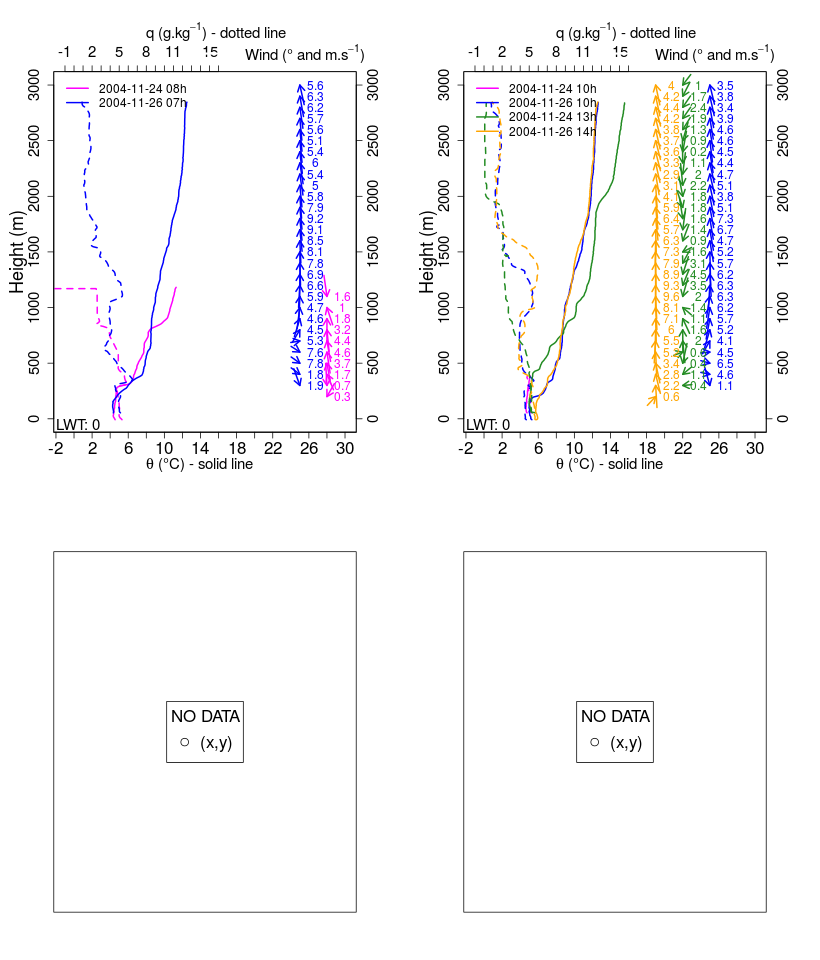

Supplement: S2 File — S2 to S12 Figs LWT from 0 to 10 (a) Mean diurnal cycles of temperature, precipitation, relative humidity, wind force, wind-rose. (b) Mean diurnal cycles of global solar radiation, downward atmospheric radiation and air pressure for Toulouse. Seasonal intra-clusters differences for winter, spring, summer and autumn are presented in grey, light pink, dark pink and brown respectively. S13 and S14 Figs Cluster frequency occurrence in total percentage (left) and per season (right) for the future projections presented in section 3.3. Spring, summer, autumn and winter seasons are in brown, pink, violet and grey respectively. S15 to S25 Figs Surface energy balance per season and per Local Weather Type from the CAPITOUL campaign. S15 corresponds to the seasonal mean. S16 to S25 corresponds to the LWT 0 to the LWT 10 respectively. S26 to S30 Figs Mean Urban Heat Island intensity per season and per Local Weather Type from the CAPITOUL campaign. S26 corresponds to LWT 0 (up) and 1 (bottom); S27 corresponds to LWT 2 (up) and 3 (bottom); S28 corresponds to LWT 4 (up) and 5 (bottom); S29 corresponds to LWT 6 (up) and 7 (bottom); S30 corresponds to LWT 8 (up) and 9 (bottom). S31 to S43 Figs Radio-soundings per Local Weather Type from the CAPITOUL campaign. S31 and S32 corresponds to LWT 0 in winter and autumn; S22 corresponds to LWT 2 in autumn; S34 to S36 corresponds to LWT3 in winter, spring and summer; S37 and S38 corresponds to LWT5 in winter and autumn; S39 corresponds to LWT 7 in summer; S40 corresponds to LWT 8 in summer, S41 to S43 corresponds to LWT 9 in winter, spring and summer respectively. (ZIP) [file pone.0208138.s003.zip › Supporting_Information/S32.tiff]

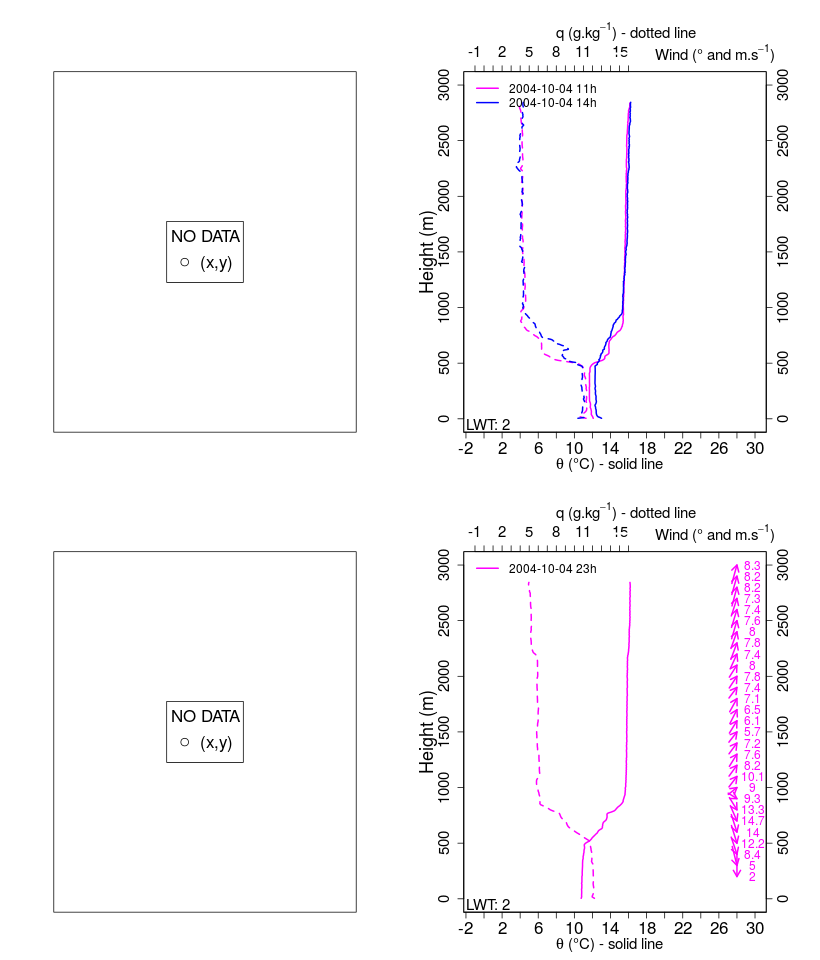

Supplement: S2 File — S2 to S12 Figs LWT from 0 to 10 (a) Mean diurnal cycles of temperature, precipitation, relative humidity, wind force, wind-rose. (b) Mean diurnal cycles of global solar radiation, downward atmospheric radiation and air pressure for Toulouse. Seasonal intra-clusters differences for winter, spring, summer and autumn are presented in grey, light pink, dark pink and brown respectively. S13 and S14 Figs Cluster frequency occurrence in total percentage (left) and per season (right) for the future projections presented in section 3.3. Spring, summer, autumn and winter seasons are in brown, pink, violet and grey respectively. S15 to S25 Figs Surface energy balance per season and per Local Weather Type from the CAPITOUL campaign. S15 corresponds to the seasonal mean. S16 to S25 corresponds to the LWT 0 to the LWT 10 respectively. S26 to S30 Figs Mean Urban Heat Island intensity per season and per Local Weather Type from the CAPITOUL campaign. S26 corresponds to LWT 0 (up) and 1 (bottom); S27 corresponds to LWT 2 (up) and 3 (bottom); S28 corresponds to LWT 4 (up) and 5 (bottom); S29 corresponds to LWT 6 (up) and 7 (bottom); S30 corresponds to LWT 8 (up) and 9 (bottom). S31 to S43 Figs Radio-soundings per Local Weather Type from the CAPITOUL campaign. S31 and S32 corresponds to LWT 0 in winter and autumn; S22 corresponds to LWT 2 in autumn; S34 to S36 corresponds to LWT3 in winter, spring and summer; S37 and S38 corresponds to LWT5 in winter and autumn; S39 corresponds to LWT 7 in summer; S40 corresponds to LWT 8 in summer, S41 to S43 corresponds to LWT 9 in winter, spring and summer respectively. (ZIP) [file pone.0208138.s003.zip › Supporting_Information/S33.tiff]

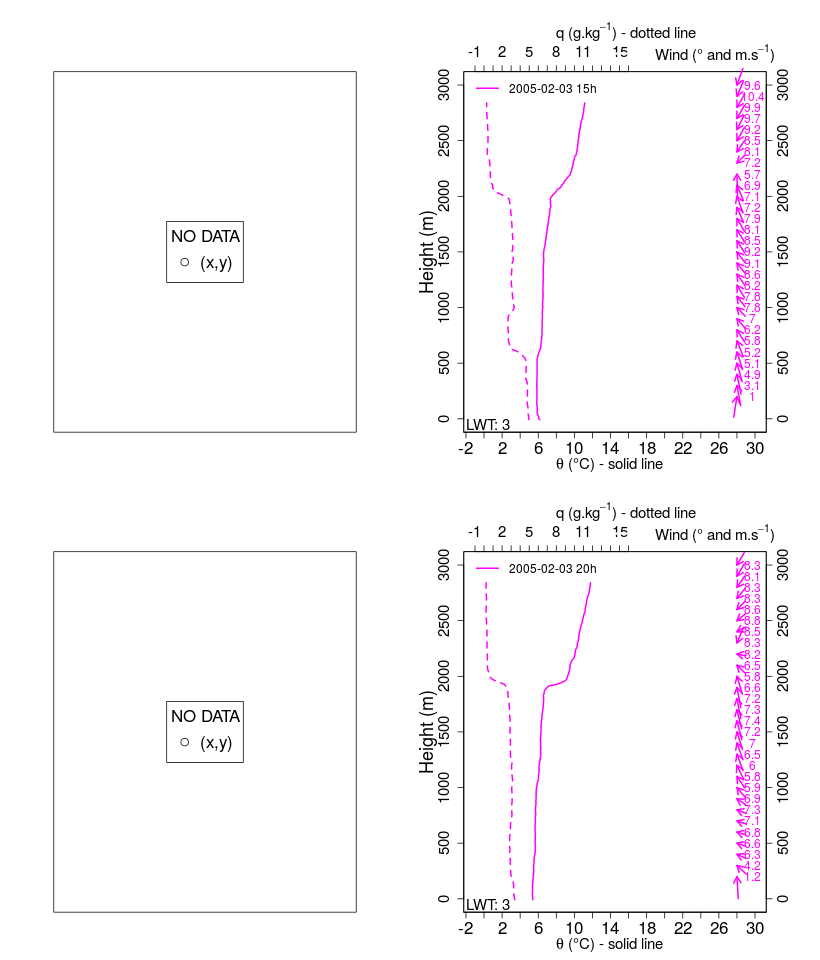

Supplement: S2 File — S2 to S12 Figs LWT from 0 to 10 (a) Mean diurnal cycles of temperature, precipitation, relative humidity, wind force, wind-rose. (b) Mean diurnal cycles of global solar radiation, downward atmospheric radiation and air pressure for Toulouse. Seasonal intra-clusters differences for winter, spring, summer and autumn are presented in grey, light pink, dark pink and brown respectively. S13 and S14 Figs Cluster frequency occurrence in total percentage (left) and per season (right) for the future projections presented in section 3.3. Spring, summer, autumn and winter seasons are in brown, pink, violet and grey respectively. S15 to S25 Figs Surface energy balance per season and per Local Weather Type from the CAPITOUL campaign. S15 corresponds to the seasonal mean. S16 to S25 corresponds to the LWT 0 to the LWT 10 respectively. S26 to S30 Figs Mean Urban Heat Island intensity per season and per Local Weather Type from the CAPITOUL campaign. S26 corresponds to LWT 0 (up) and 1 (bottom); S27 corresponds to LWT 2 (up) and 3 (bottom); S28 corresponds to LWT 4 (up) and 5 (bottom); S29 corresponds to LWT 6 (up) and 7 (bottom); S30 corresponds to LWT 8 (up) and 9 (bottom). S31 to S43 Figs Radio-soundings per Local Weather Type from the CAPITOUL campaign. S31 and S32 corresponds to LWT 0 in winter and autumn; S22 corresponds to LWT 2 in autumn; S34 to S36 corresponds to LWT3 in winter, spring and summer; S37 and S38 corresponds to LWT5 in winter and autumn; S39 corresponds to LWT 7 in summer; S40 corresponds to LWT 8 in summer, S41 to S43 corresponds to LWT 9 in winter, spring and summer respectively. (ZIP) [file pone.0208138.s003.zip › Supporting_Information/S34.tiff]

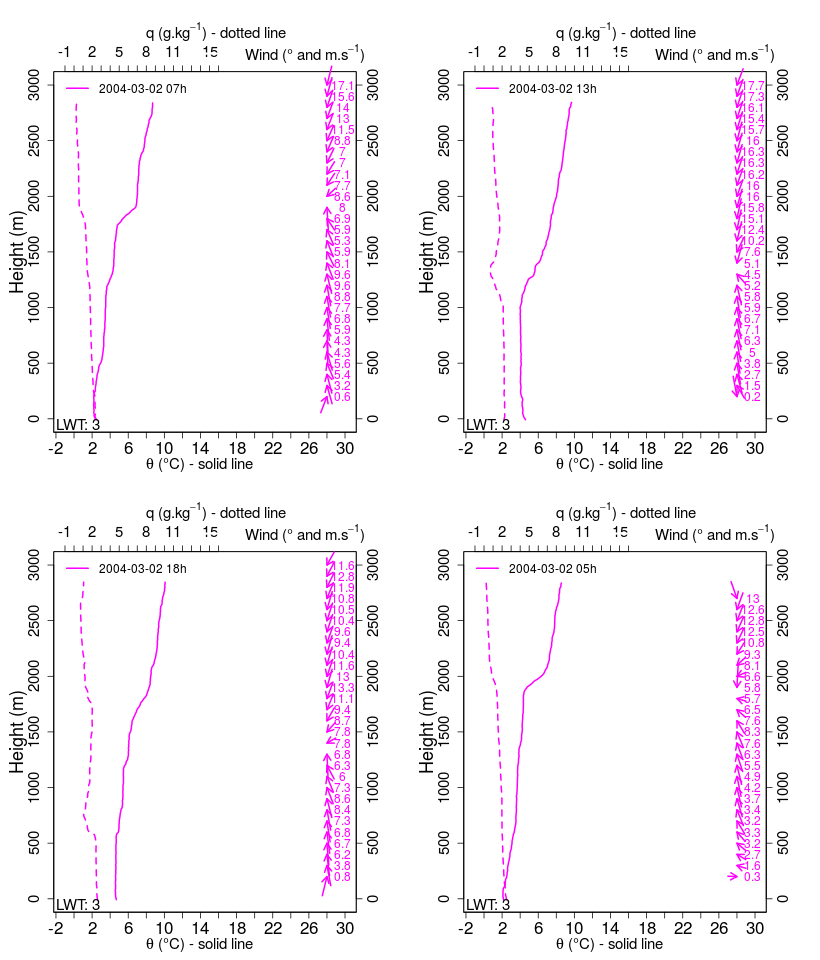

Supplement: S2 File — S2 to S12 Figs LWT from 0 to 10 (a) Mean diurnal cycles of temperature, precipitation, relative humidity, wind force, wind-rose. (b) Mean diurnal cycles of global solar radiation, downward atmospheric radiation and air pressure for Toulouse. Seasonal intra-clusters differences for winter, spring, summer and autumn are presented in grey, light pink, dark pink and brown respectively. S13 and S14 Figs Cluster frequency occurrence in total percentage (left) and per season (right) for the future projections presented in section 3.3. Spring, summer, autumn and winter seasons are in brown, pink, violet and grey respectively. S15 to S25 Figs Surface energy balance per season and per Local Weather Type from the CAPITOUL campaign. S15 corresponds to the seasonal mean. S16 to S25 corresponds to the LWT 0 to the LWT 10 respectively. S26 to S30 Figs Mean Urban Heat Island intensity per season and per Local Weather Type from the CAPITOUL campaign. S26 corresponds to LWT 0 (up) and 1 (bottom); S27 corresponds to LWT 2 (up) and 3 (bottom); S28 corresponds to LWT 4 (up) and 5 (bottom); S29 corresponds to LWT 6 (up) and 7 (bottom); S30 corresponds to LWT 8 (up) and 9 (bottom). S31 to S43 Figs Radio-soundings per Local Weather Type from the CAPITOUL campaign. S31 and S32 corresponds to LWT 0 in winter and autumn; S22 corresponds to LWT 2 in autumn; S34 to S36 corresponds to LWT3 in winter, spring and summer; S37 and S38 corresponds to LWT5 in winter and autumn; S39 corresponds to LWT 7 in summer; S40 corresponds to LWT 8 in summer, S41 to S43 corresponds to LWT 9 in winter, spring and summer respectively. (ZIP) [file pone.0208138.s003.zip › Supporting_Information/S35.tiff]

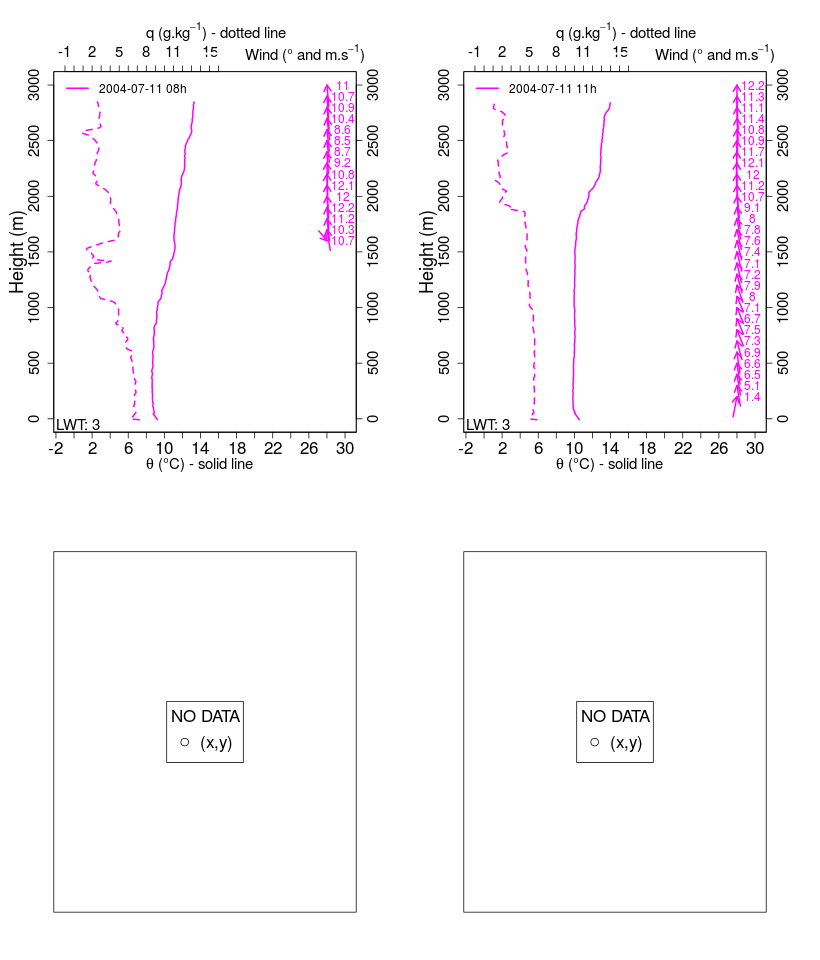

Supplement: S2 File — S2 to S12 Figs LWT from 0 to 10 (a) Mean diurnal cycles of temperature, precipitation, relative humidity, wind force, wind-rose. (b) Mean diurnal cycles of global solar radiation, downward atmospheric radiation and air pressure for Toulouse. Seasonal intra-clusters differences for winter, spring, summer and autumn are presented in grey, light pink, dark pink and brown respectively. S13 and S14 Figs Cluster frequency occurrence in total percentage (left) and per season (right) for the future projections presented in section 3.3. Spring, summer, autumn and winter seasons are in brown, pink, violet and grey respectively. S15 to S25 Figs Surface energy balance per season and per Local Weather Type from the CAPITOUL campaign. S15 corresponds to the seasonal mean. S16 to S25 corresponds to the LWT 0 to the LWT 10 respectively. S26 to S30 Figs Mean Urban Heat Island intensity per season and per Local Weather Type from the CAPITOUL campaign. S26 corresponds to LWT 0 (up) and 1 (bottom); S27 corresponds to LWT 2 (up) and 3 (bottom); S28 corresponds to LWT 4 (up) and 5 (bottom); S29 corresponds to LWT 6 (up) and 7 (bottom); S30 corresponds to LWT 8 (up) and 9 (bottom). S31 to S43 Figs Radio-soundings per Local Weather Type from the CAPITOUL campaign. S31 and S32 corresponds to LWT 0 in winter and autumn; S22 corresponds to LWT 2 in autumn; S34 to S36 corresponds to LWT3 in winter, spring and summer; S37 and S38 corresponds to LWT5 in winter and autumn; S39 corresponds to LWT 7 in summer; S40 corresponds to LWT 8 in summer, S41 to S43 corresponds to LWT 9 in winter, spring and summer respectively. (ZIP) [file pone.0208138.s003.zip › Supporting_Information/S36.tiff]

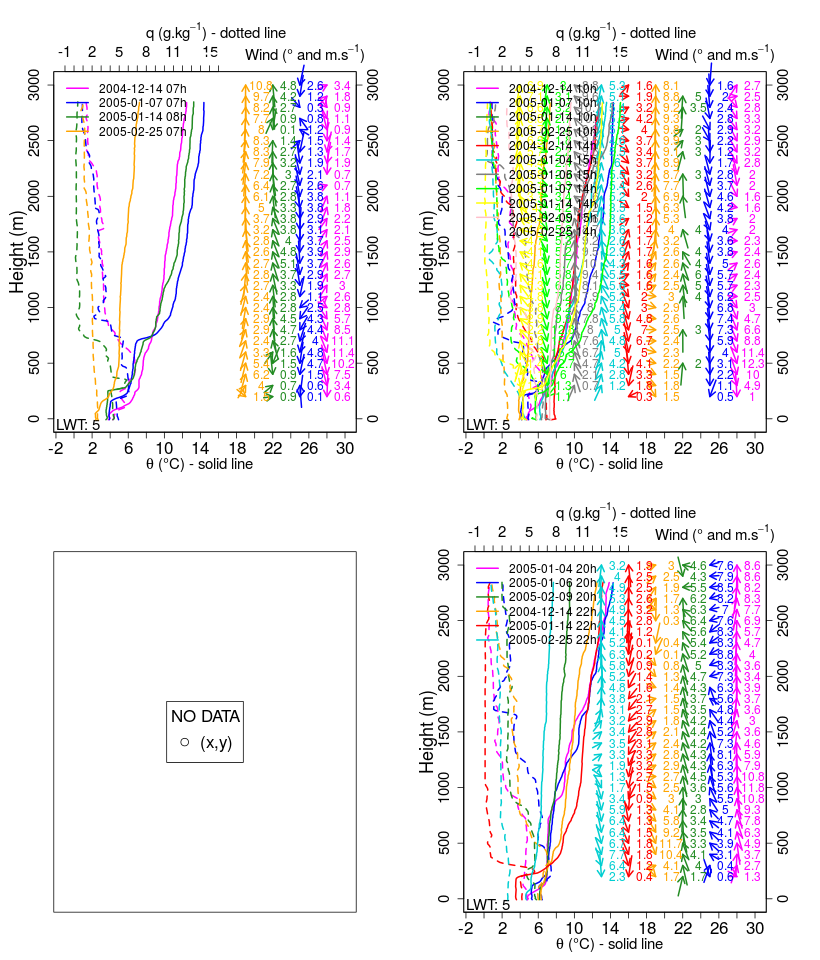

Supplement: S2 File — S2 to S12 Figs LWT from 0 to 10 (a) Mean diurnal cycles of temperature, precipitation, relative humidity, wind force, wind-rose. (b) Mean diurnal cycles of global solar radiation, downward atmospheric radiation and air pressure for Toulouse. Seasonal intra-clusters differences for winter, spring, summer and autumn are presented in grey, light pink, dark pink and brown respectively. S13 and S14 Figs Cluster frequency occurrence in total percentage (left) and per season (right) for the future projections presented in section 3.3. Spring, summer, autumn and winter seasons are in brown, pink, violet and grey respectively. S15 to S25 Figs Surface energy balance per season and per Local Weather Type from the CAPITOUL campaign. S15 corresponds to the seasonal mean. S16 to S25 corresponds to the LWT 0 to the LWT 10 respectively. S26 to S30 Figs Mean Urban Heat Island intensity per season and per Local Weather Type from the CAPITOUL campaign. S26 corresponds to LWT 0 (up) and 1 (bottom); S27 corresponds to LWT 2 (up) and 3 (bottom); S28 corresponds to LWT 4 (up) and 5 (bottom); S29 corresponds to LWT 6 (up) and 7 (bottom); S30 corresponds to LWT 8 (up) and 9 (bottom). S31 to S43 Figs Radio-soundings per Local Weather Type from the CAPITOUL campaign. S31 and S32 corresponds to LWT 0 in winter and autumn; S22 corresponds to LWT 2 in autumn; S34 to S36 corresponds to LWT3 in winter, spring and summer; S37 and S38 corresponds to LWT5 in winter and autumn; S39 corresponds to LWT 7 in summer; S40 corresponds to LWT 8 in summer, S41 to S43 corresponds to LWT 9 in winter, spring and summer respectively. (ZIP) [file pone.0208138.s003.zip › Supporting_Information/S37.tiff]

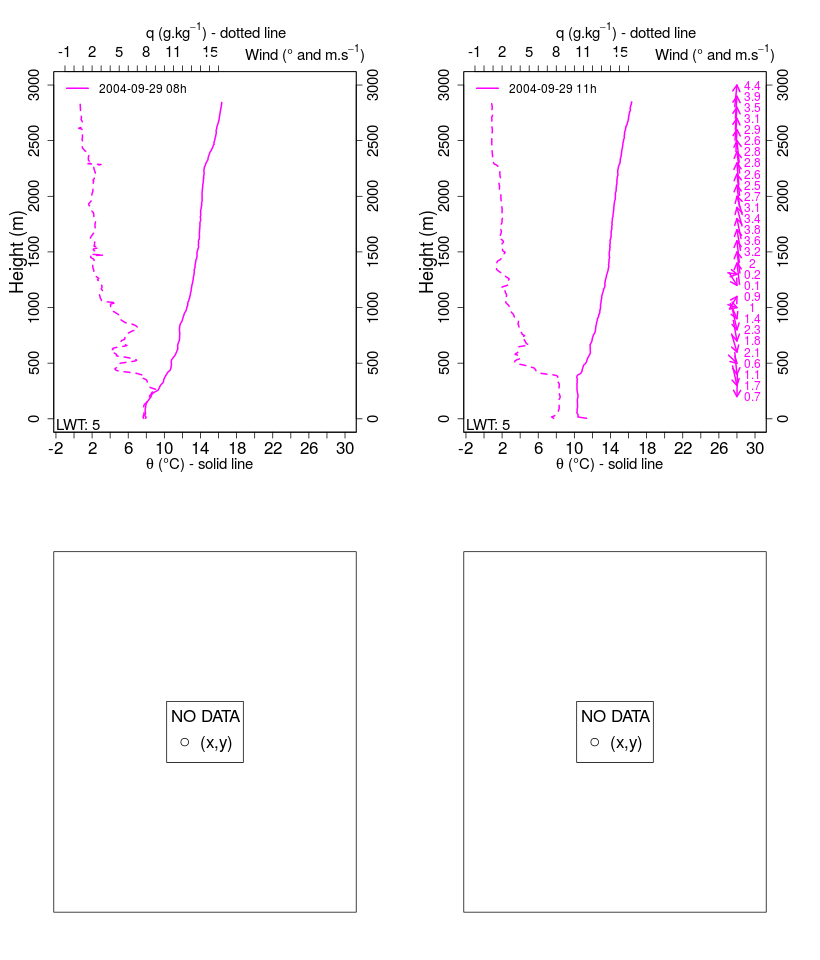

Supplement: S2 File — S2 to S12 Figs LWT from 0 to 10 (a) Mean diurnal cycles of temperature, precipitation, relative humidity, wind force, wind-rose. (b) Mean diurnal cycles of global solar radiation, downward atmospheric radiation and air pressure for Toulouse. Seasonal intra-clusters differences for winter, spring, summer and autumn are presented in grey, light pink, dark pink and brown respectively. S13 and S14 Figs Cluster frequency occurrence in total percentage (left) and per season (right) for the future projections presented in section 3.3. Spring, summer, autumn and winter seasons are in brown, pink, violet and grey respectively. S15 to S25 Figs Surface energy balance per season and per Local Weather Type from the CAPITOUL campaign. S15 corresponds to the seasonal mean. S16 to S25 corresponds to the LWT 0 to the LWT 10 respectively. S26 to S30 Figs Mean Urban Heat Island intensity per season and per Local Weather Type from the CAPITOUL campaign. S26 corresponds to LWT 0 (up) and 1 (bottom); S27 corresponds to LWT 2 (up) and 3 (bottom); S28 corresponds to LWT 4 (up) and 5 (bottom); S29 corresponds to LWT 6 (up) and 7 (bottom); S30 corresponds to LWT 8 (up) and 9 (bottom). S31 to S43 Figs Radio-soundings per Local Weather Type from the CAPITOUL campaign. S31 and S32 corresponds to LWT 0 in winter and autumn; S22 corresponds to LWT 2 in autumn; S34 to S36 corresponds to LWT3 in winter, spring and summer; S37 and S38 corresponds to LWT5 in winter and autumn; S39 corresponds to LWT 7 in summer; S40 corresponds to LWT 8 in summer, S41 to S43 corresponds to LWT 9 in winter, spring and summer respectively. (ZIP) [file pone.0208138.s003.zip › Supporting_Information/S38.tiff]

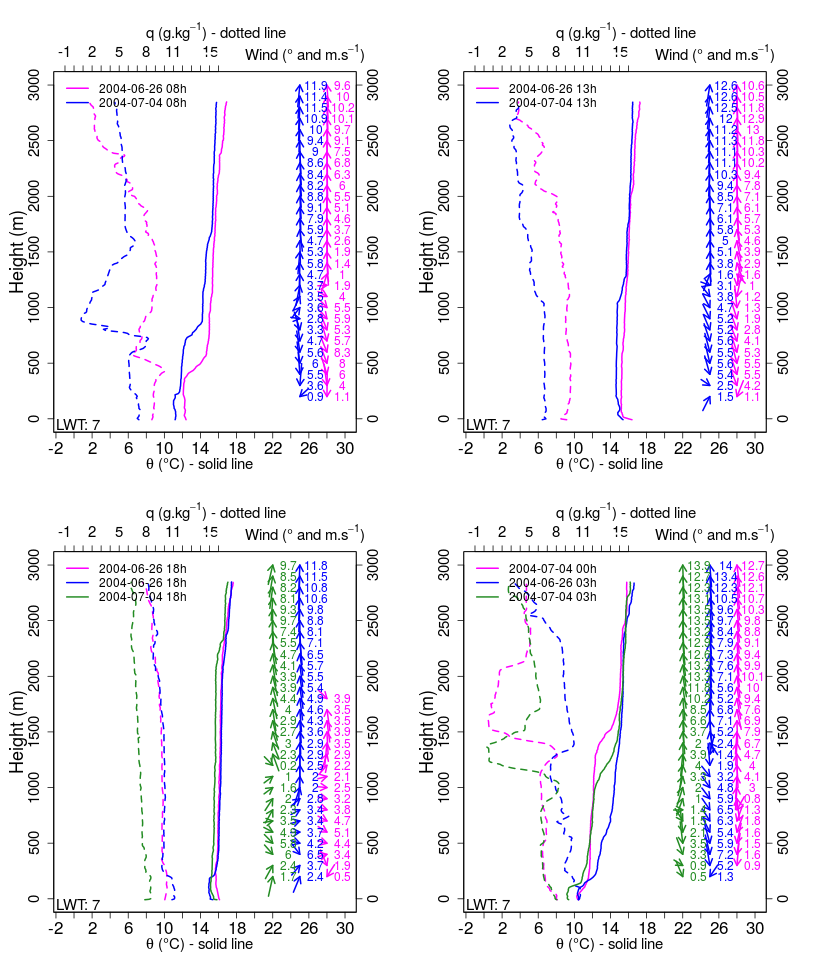

Supplement: S2 File — S2 to S12 Figs LWT from 0 to 10 (a) Mean diurnal cycles of temperature, precipitation, relative humidity, wind force, wind-rose. (b) Mean diurnal cycles of global solar radiation, downward atmospheric radiation and air pressure for Toulouse. Seasonal intra-clusters differences for winter, spring, summer and autumn are presented in grey, light pink, dark pink and brown respectively. S13 and S14 Figs Cluster frequency occurrence in total percentage (left) and per season (right) for the future projections presented in section 3.3. Spring, summer, autumn and winter seasons are in brown, pink, violet and grey respectively. S15 to S25 Figs Surface energy balance per season and per Local Weather Type from the CAPITOUL campaign. S15 corresponds to the seasonal mean. S16 to S25 corresponds to the LWT 0 to the LWT 10 respectively. S26 to S30 Figs Mean Urban Heat Island intensity per season and per Local Weather Type from the CAPITOUL campaign. S26 corresponds to LWT 0 (up) and 1 (bottom); S27 corresponds to LWT 2 (up) and 3 (bottom); S28 corresponds to LWT 4 (up) and 5 (bottom); S29 corresponds to LWT 6 (up) and 7 (bottom); S30 corresponds to LWT 8 (up) and 9 (bottom). S31 to S43 Figs Radio-soundings per Local Weather Type from the CAPITOUL campaign. S31 and S32 corresponds to LWT 0 in winter and autumn; S22 corresponds to LWT 2 in autumn; S34 to S36 corresponds to LWT3 in winter, spring and summer; S37 and S38 corresponds to LWT5 in winter and autumn; S39 corresponds to LWT 7 in summer; S40 corresponds to LWT 8 in summer, S41 to S43 corresponds to LWT 9 in winter, spring and summer respectively. (ZIP) [file pone.0208138.s003.zip › Supporting_Information/S39.tiff]

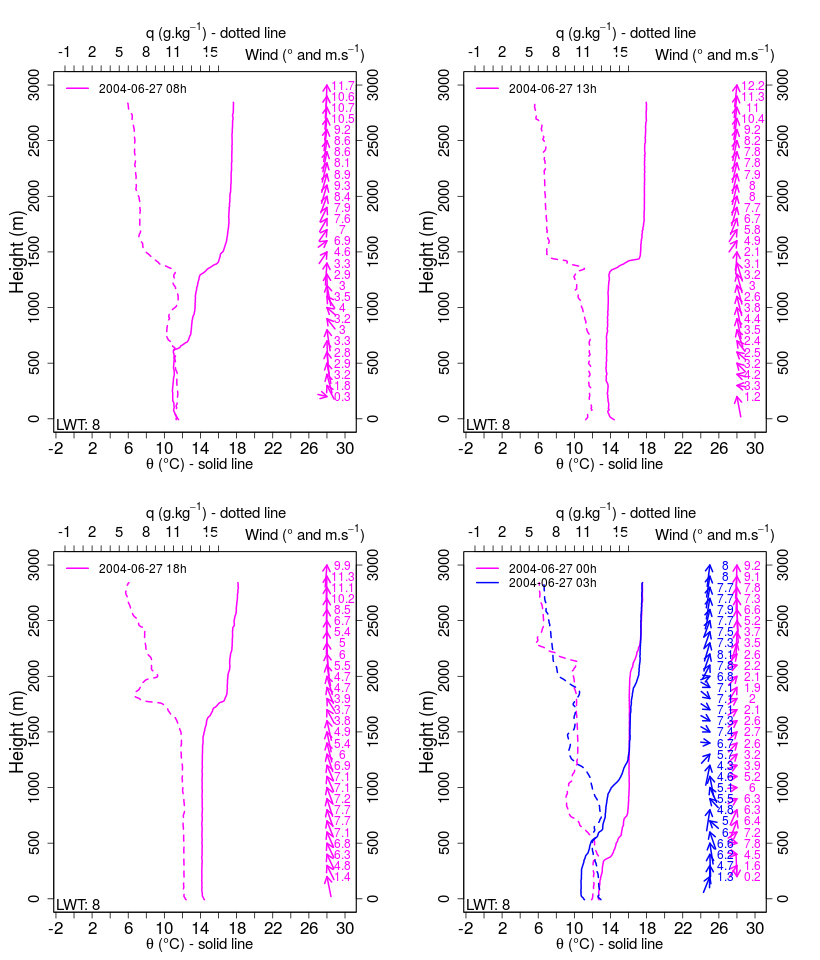

Supplement: S2 File — S2 to S12 Figs LWT from 0 to 10 (a) Mean diurnal cycles of temperature, precipitation, relative humidity, wind force, wind-rose. (b) Mean diurnal cycles of global solar radiation, downward atmospheric radiation and air pressure for Toulouse. Seasonal intra-clusters differences for winter, spring, summer and autumn are presented in grey, light pink, dark pink and brown respectively. S13 and S14 Figs Cluster frequency occurrence in total percentage (left) and per season (right) for the future projections presented in section 3.3. Spring, summer, autumn and winter seasons are in brown, pink, violet and grey respectively. S15 to S25 Figs Surface energy balance per season and per Local Weather Type from the CAPITOUL campaign. S15 corresponds to the seasonal mean. S16 to S25 corresponds to the LWT 0 to the LWT 10 respectively. S26 to S30 Figs Mean Urban Heat Island intensity per season and per Local Weather Type from the CAPITOUL campaign. S26 corresponds to LWT 0 (up) and 1 (bottom); S27 corresponds to LWT 2 (up) and 3 (bottom); S28 corresponds to LWT 4 (up) and 5 (bottom); S29 corresponds to LWT 6 (up) and 7 (bottom); S30 corresponds to LWT 8 (up) and 9 (bottom). S31 to S43 Figs Radio-soundings per Local Weather Type from the CAPITOUL campaign. S31 and S32 corresponds to LWT 0 in winter and autumn; S22 corresponds to LWT 2 in autumn; S34 to S36 corresponds to LWT3 in winter, spring and summer; S37 and S38 corresponds to LWT5 in winter and autumn; S39 corresponds to LWT 7 in summer; S40 corresponds to LWT 8 in summer, S41 to S43 corresponds to LWT 9 in winter, spring and summer respectively. (ZIP) [file pone.0208138.s003.zip › Supporting_Information/S40.tiff]

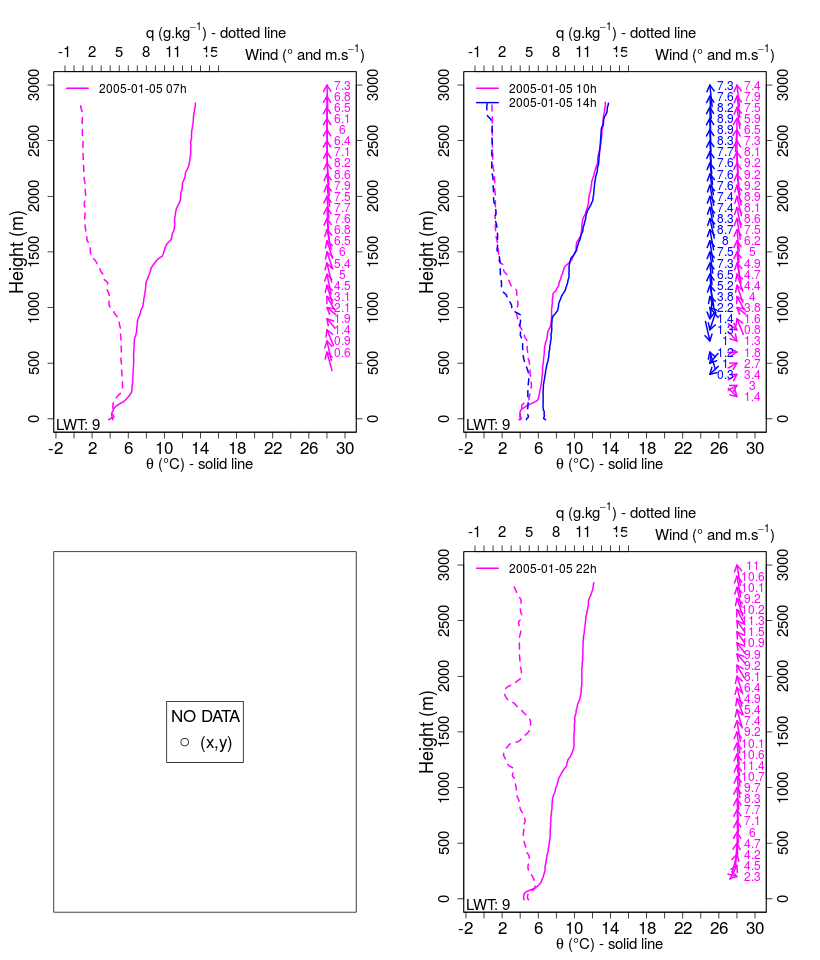

Supplement: S2 File — S2 to S12 Figs LWT from 0 to 10 (a) Mean diurnal cycles of temperature, precipitation, relative humidity, wind force, wind-rose. (b) Mean diurnal cycles of global solar radiation, downward atmospheric radiation and air pressure for Toulouse. Seasonal intra-clusters differences for winter, spring, summer and autumn are presented in grey, light pink, dark pink and brown respectively. S13 and S14 Figs Cluster frequency occurrence in total percentage (left) and per season (right) for the future projections presented in section 3.3. Spring, summer, autumn and winter seasons are in brown, pink, violet and grey respectively. S15 to S25 Figs Surface energy balance per season and per Local Weather Type from the CAPITOUL campaign. S15 corresponds to the seasonal mean. S16 to S25 corresponds to the LWT 0 to the LWT 10 respectively. S26 to S30 Figs Mean Urban Heat Island intensity per season and per Local Weather Type from the CAPITOUL campaign. S26 corresponds to LWT 0 (up) and 1 (bottom); S27 corresponds to LWT 2 (up) and 3 (bottom); S28 corresponds to LWT 4 (up) and 5 (bottom); S29 corresponds to LWT 6 (up) and 7 (bottom); S30 corresponds to LWT 8 (up) and 9 (bottom). S31 to S43 Figs Radio-soundings per Local Weather Type from the CAPITOUL campaign. S31 and S32 corresponds to LWT 0 in winter and autumn; S22 corresponds to LWT 2 in autumn; S34 to S36 corresponds to LWT3 in winter, spring and summer; S37 and S38 corresponds to LWT5 in winter and autumn; S39 corresponds to LWT 7 in summer; S40 corresponds to LWT 8 in summer, S41 to S43 corresponds to LWT 9 in winter, spring and summer respectively. (ZIP) [file pone.0208138.s003.zip › Supporting_Information/S41.tiff]

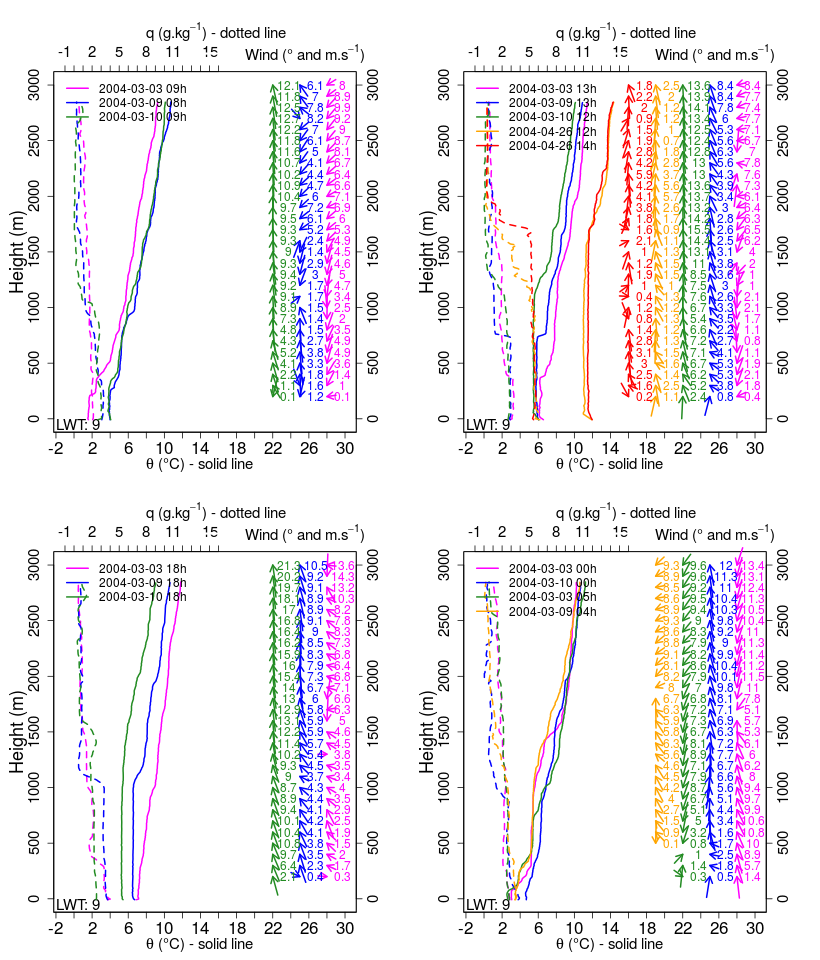

Supplement: S2 File — S2 to S12 Figs LWT from 0 to 10 (a) Mean diurnal cycles of temperature, precipitation, relative humidity, wind force, wind-rose. (b) Mean diurnal cycles of global solar radiation, downward atmospheric radiation and air pressure for Toulouse. Seasonal intra-clusters differences for winter, spring, summer and autumn are presented in grey, light pink, dark pink and brown respectively. S13 and S14 Figs Cluster frequency occurrence in total percentage (left) and per season (right) for the future projections presented in section 3.3. Spring, summer, autumn and winter seasons are in brown, pink, violet and grey respectively. S15 to S25 Figs Surface energy balance per season and per Local Weather Type from the CAPITOUL campaign. S15 corresponds to the seasonal mean. S16 to S25 corresponds to the LWT 0 to the LWT 10 respectively. S26 to S30 Figs Mean Urban Heat Island intensity per season and per Local Weather Type from the CAPITOUL campaign. S26 corresponds to LWT 0 (up) and 1 (bottom); S27 corresponds to LWT 2 (up) and 3 (bottom); S28 corresponds to LWT 4 (up) and 5 (bottom); S29 corresponds to LWT 6 (up) and 7 (bottom); S30 corresponds to LWT 8 (up) and 9 (bottom). S31 to S43 Figs Radio-soundings per Local Weather Type from the CAPITOUL campaign. S31 and S32 corresponds to LWT 0 in winter and autumn; S22 corresponds to LWT 2 in autumn; S34 to S36 corresponds to LWT3 in winter, spring and summer; S37 and S38 corresponds to LWT5 in winter and autumn; S39 corresponds to LWT 7 in summer; S40 corresponds to LWT 8 in summer, S41 to S43 corresponds to LWT 9 in winter, spring and summer respectively. (ZIP) [file pone.0208138.s003.zip › Supporting_Information/S42.tiff]

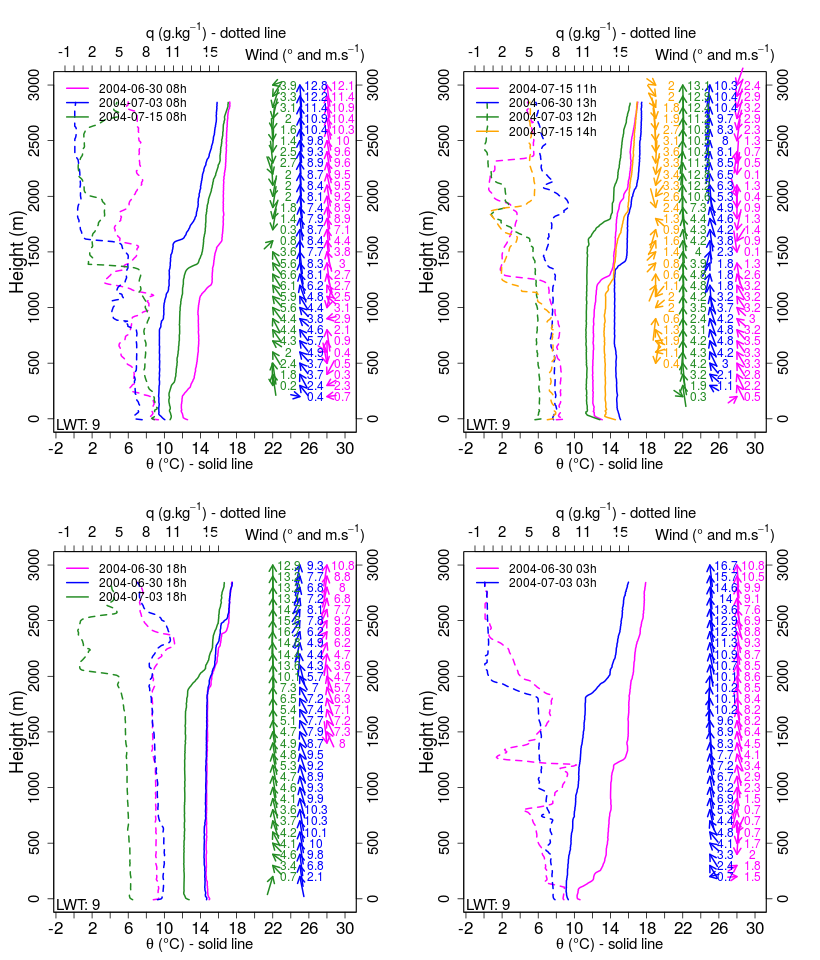

Supplement: S2 File — S2 to S12 Figs LWT from 0 to 10 (a) Mean diurnal cycles of temperature, precipitation, relative humidity, wind force, wind-rose. (b) Mean diurnal cycles of global solar radiation, downward atmospheric radiation and air pressure for Toulouse. Seasonal intra-clusters differences for winter, spring, summer and autumn are presented in grey, light pink, dark pink and brown respectively. S13 and S14 Figs Cluster frequency occurrence in total percentage (left) and per season (right) for the future projections presented in section 3.3. Spring, summer, autumn and winter seasons are in brown, pink, violet and grey respectively. S15 to S25 Figs Surface energy balance per season and per Local Weather Type from the CAPITOUL campaign. S15 corresponds to the seasonal mean. S16 to S25 corresponds to the LWT 0 to the LWT 10 respectively. S26 to S30 Figs Mean Urban Heat Island intensity per season and per Local Weather Type from the CAPITOUL campaign. S26 corresponds to LWT 0 (up) and 1 (bottom); S27 corresponds to LWT 2 (up) and 3 (bottom); S28 corresponds to LWT 4 (up) and 5 (bottom); S29 corresponds to LWT 6 (up) and 7 (bottom); S30 corresponds to LWT 8 (up) and 9 (bottom). S31 to S43 Figs Radio-soundings per Local Weather Type from the CAPITOUL campaign. S31 and S32 corresponds to LWT 0 in winter and autumn; S22 corresponds to LWT 2 in autumn; S34 to S36 corresponds to LWT3 in winter, spring and summer; S37 and S38 corresponds to LWT5 in winter and autumn; S39 corresponds to LWT 7 in summer; S40 corresponds to LWT 8 in summer, S41 to S43 corresponds to LWT 9 in winter, spring and summer respectively. (ZIP) [file pone.0208138.s003.zip › Supporting_Information/S43.tiff]
